# A revision of the genus *Planinasus* Cresson (Diptera, Periscelididae)

**DOI:** 10.3897/zookeys.225.3721

**Published:** 2012-10-02

**Authors:** Wayne N. Mathis, Alessandra Rung, Marion Kotrba

**Affiliations:** 1 Department of Entomology, NHB 169, PO BOX 37012; Smithsonian Institution, Washington, D.C. 20013-7012, USA; 2 Plant Pest Diagnositics Branch, California Department of Food and Agriculture, Sacramento, CA 95832, USA; 3 Zoologische Staatssammlung München, Münchhausenstrasse 21, 81247 München, Germany

**Keywords:** Diptera, Periscelididae, Stenomicrinae, *Planinasus*, New World, new species

## Abstract

The genus *Planinasus* Cresson is revised and includes 18 extant and one fossil species. We clarify the status of the three previously described species and describe 15 new species as follows (type locality in parenthesis): *Planinasus aenigmaticus* (Colombia. Bogota: Bogota (04°35.8'N, 74°08.8'W)), *Planinasus neotropicus* (Panama. Canal Zone: Barro Colorado Island (09°09.1'N, 79°50.8'W)), *Planinasus kotrbae* (Ecuador. Orellana: Rio Tiputini Biodiversity Station (0°38.2'S, 76°08.9'W)), *Planinasus miradorus* (Brazil. Maranhão: Parque Estadual Mirador, Base da Geraldina (06°22.2'S, 44°21.8'W)), *Planinasus tobagoensis* (Trinidad and Tobago. Tobago. St. John: Parlatuvier (11°17.9'N, 60°39'W)), *Planinasus xanthops* (Ecuador. Orellana: Rio Tiputini Biodiversity Station (0°38.2'S, 76°8.9'W)), *Planinasus argentifacies* (Peru. Madre de Dios: Río Manu, Pakitza (11°56.6'S, 71°16.9'W; 250 m)), *Planinasus insulanus* (Dominican Republic. La Vega: near Jarabacoa, Salto Guasara (19°04.4'N, 70°42.1'W, 680 m)), *Planinasus nigritarsus* (Guyana. Conservation of Ecological Interactions and Biotic Associations (CEIBA; ca. 40 km S Georgetown; 06°29.9'N, 58°13.1'W)), *Planinasus atriclypeus* (Brazil. Rio de Janeiro: Rio de Janeiro, Floresta da Tijuca (22°57.6'S, 43°16.4'W)), *Planinasus atrifrons* (Bolivia. Santa Cruz: Ichilo, Buena Vista (4-6 km SSE; Hotel Flora y Fauna; 17°29.95'S, 63°33.15'W; 4-500 m)), P. *flavicoxalis* (West Indies. Dominica. St. David: 1.6 km N of junction of roads to Rosalie and Castle Bruce (15°23.8'N, 61°18.6'W)), *Planinasus mcalpineorum* (Mexico. Chiapas: Cacahoatan (7 km N; 15°04.1'N, 92°07.4'W)), *Planinasus nigrifacies* (Brazil. São Paulo: Mogi das Cruzes, Serra do Itapeti (23°31.5'S, 46°11.2'W)), *Planinasus obscuripennis* (Peru. Madre de Dios: Río Manu, Erika (near Salvación; 12°50.7'S, 71°23.3'W; 550 m)). In addition to external characters, we also describe and illustrate structures of the male terminalia and for *Planinasus kotrbae* **sp. n.**, the internal female reproductive organs. Detailed locality data and distribution maps for all species are provided. For perspective and to facilitate genus-group and species-group recognition, the family Periscelididae and subfamily Stenomicrinae are diagnosed and for the latter, a key to included genera is provided.

## Introduction

Flies of the genus *Planinasus* Cresson are uncommon in most collections and perhaps in nature. Their scarcity, coupled with their unusually wide heads (hypercephaly)--a feature that is more pronounced in males of some species--makes them even more of a curiosity and inviting subjects for study. As would be expected for a group that is not typically well represented in collections, the species are poorly known, and thus far, only three extant species and one fossil have been described (Prado 1975, Grimaldi and Mathis 1993, Mathis and Rung 2011).

*Planinasus* is known only from the New World tropics, and recent collecting there, especially in Bolivia, Brazil, Ecuador, Guyana, Mexico, Peru, and on islands of the Caribbean, has resulted in discovery of several additional species, all undescribed. Description of these species within the context of a species revision is the purpose of this paper.

The phylogenetic position of *Planinasus* relative to other Diptera has been confused, and while these relationships are somewhat clearer now (Winkler et al. 2010, Mathis and Rung 2011), definitive resolution continues to elude us. Cresson (1914) described *Planinasus* in the family Ephydridae, although he later suggested (1918) that the genus was probably not an ephydrid. Malloch (1934) first recognized *Planinasus* as a genus in the family Periscelididae, but that precedent was not accepted by all his successors. Curran (1934), for example, placed the genus in the family Drosophilidae, and Hennig (1969) preferred the family Aulacigastridae. More recently, McAlpine (1983) again assigned the genus to Periscelididae, and we also subscribe to that precedent (Baptista and Mathis 1994, Mathis and Rung 2011) with qualification and herein present additional evidence and discussion that explains that phylogenetic placement.

The nomenclatural history of the genus and its included species is uncomplicated, as would be expected for a little-known genus that comprises few species. When Cresson (1914) first proposed the genus, it was monotypic, with *Planinasus ambiguus* Cresson as its type species. Malloch (1934) described a second genus, *Schizochaeta*, which was also monotypic, being based on *Schizochaeta shannoni*, a new species that Malloch described in the same paper. Hennig (1969), who reviewed the group, suggested that *Planinasus ambiguus* and *Schizochaeta shannoni* were congeneric, synonymized *Schizochaeta* with *Planinasus*, and described a third species, *Planinasus venezuelensis*. Grimaldi and Mathis (1993) described the fourth species, *Planinasus electrus*, which is a fossil preserved in Dominican Amber. Except for McAlpine’s (1978, 1983), Winkler et al. (2010) and Mathis and Rung’s (2011) discussion of placement of *Planinasus* in the family Periscelididae and phylogenetic relationships, the genus has not been treated substantively by others.

## Methods and materials

Most of the species considered herein are newly described, and in their individual treatments, we have included all specimens that were available and that could be reliably identified. Specimens included in type series are more restricted, particularly those for which identifications are based primarily on characters of the male terminalia. Other specimens are listed with the appropriate species under the section “Other specimens examined.” Type specimens of all nominate taxa were studied. Label data accompanying each holotype are quoted verbatim with a slash mark to separate data of one label from another. Clarifying or interpretive comments are included within brackets.

Descriptions of species are composite if specimens other than the holotype were available. For the most part, information given in the generic description is not repeated in the species descriptions. The descriptive terminology follows that published in the recent *Manual of Nearctic Diptera* (McAlpine 1981) with the modification noted below. We follow Sabrosky (1983) in using the term “microtomentum” rather than pruinosity. We use the term basal flagellomere for the large antennomere beyond the pedicel. We prefer this term over “first flagellomere” as there may be more than one flagellomere involved, and basal does not imply a number or numbers. We likewise do not use “postpedicel” (Stuckenberg 1999) for this antennomere because at least the multisegmented arista is beyond the pedicel in addition to the large antennomere, and postpedicel is thus ambiguous and lacking in precision.

We have adopted the nomenclature of Cumming et al. (1995) for describing structures of the male terminalia with the following exception: we use the term pregonite with reference to the basal plate closely associated with the arms of the hypandrium and the bacilliform sclerites; and the term postgonite with reference to the complex clasper-like structure closely associated with the phallapodeme basally, the phallus medially, and the pregonites laterally. The usage of these terms follows Griffiths (1972) in that they are provisional and do not indicate any firm opinion on homology. One of these structures, probably the postgonite, seems to correspond to the “gonostyles” of Cumming et al. (1995). We described the diagnostic features of the male terminalia from three views: lateral view of the epandrium (including the hypandrium when attached); ventral view of the epandrium and internal structures; and lateral view of the internal structures. We also provide an illustration and description of the ejaculatory apodeme. When the epandrium and associated structures were illustrated in ventral view, some parts of the image, including the epandrium, were not done in the same exact position, and to avoid ambiguities of interpretation that could arise when observing structures from this view, we concentrate on the shape of the hypandrium, pregonite and lobe and setae of the postgonite.

Because specimens are small, usually less than 3.50 mm in length, study and illustration of structures of the male terminalia and female reproductive organs required use of a compound microscope. Observation of detailed structures of the male terminalia was sometimes needed to confirm a species’ identification.

Dissections of male terminalia were performed following Clausen and Cook (1971) and Grimaldi (1987). Abdomens were removed with microforceps and macerated in a sodium hydroxide solution. Cleared genitalia were then transferred to glycerin for observation. The dissected abdomen was placed in a plastic microvial filled with glycerin and attached to the pin supporting the remainder of the insect from which it was removed. The female reproductive organs were dissected from freshly collected specimens and embedded directly in polyvinyllactophenol with an admixture of chlorazol black E, which stains the unsclerotized cuticle blue.

Distribution maps were made using ESRI ArcView7 GIS 3.2. Longitude and latitude coordinates were obtained for the locality where each specimen was collected and entered into a Microsoft Excel^©^ spreadsheet. If unavailable directly from specimen labels, longitude and latitude were estimated using gazetteers and maps, usually available electronically, to determine the geographical coordinates.

Three head ratios are used commonly in the descriptions and are defined here for the convenience of the user (ratios are averages of three specimens).

1. Head ratio: head height (distance from peristomal margin to vertex)/head width. The measurements were taken from the head from an anterior view.

2. Facial ratio: interantennal width (shortest distance between antennal bases)/facial height (distance in the middle of the face between the ptilinal suture and the ventralmost margin of the face). The measurements were taken from the head from an anterior view.

3. Frontal ratio: frontal width (straight line distance between compound eyes at the level of the anterior ocellus)/frontal height (distance between the ptilinal suture (anterior margin of the frons) and the vertex. The measurements were taken from the head with an orientation to make the frons flat in the field of view.

The author’s contributions generally adhered to the following division of labor: most of the descriptive work was done by the first two authors, and the species names are attributed to them; the third author contributed information on the female reproductive system and observations on mating behavior. All three participated in field work together in Ecuador, where many of the specimens were collected and where observations and preliminary dissections were made.

Although this study was based primarily on specimens in the National Museum of Natural History, Smithsonian Institution (USNM), numerous others were borrowed, particularly primary type specimens of the species described previously. To our colleagues and their institutions listed below who lent specimens, we express our sincere thanks. Without their cooperation this study could not have been completed.

AMNH American Museum of Natural History, New York, New York (David A. Grimaldi)

ANSP Academy of Natural Sciences of Philadelphia, Pennsylvania (Jon K. Gelhaus, and Jason D. Weintraub)

CAS California Academy of Sciences, San Francisco, California (Paul H. Arnaud, Jr.)

CNC Canadian National Collection, Ottawa, Canada (Owen Lonsdale, J. Frank McAlpine)

DEBU University of Guelph, Guelph, Ontario, Canada (Stephen A. Marshall)

INBIO Insituto Nacional de Biodiversidad, Santo Domingo, Costa Rica (Manuel Zumbado)

INPA Instituto Nacional de Pesquisas da Amazônia, Manaus, Amazonas, Brazil (José Albertino Rafael, Rosaly Ale-Rocha)

ZSMC Zoologische Staatssammlung München, München, Germany (Marion Kotrba)

## Systematics

To give perspective to this revision, we are providing diagnoses for the family Periscelididae and the subfamily Stenomicrinae. Also included is a key to subfamilies and the included genera of Stenomicrinae. The keys provide convenient characters in a succinct format for identifying the genera without necessarily implying any relationships among them. Indeed, the reader is cautioned not to infer generic relationships from the included keys, especially regarding *Planinasus*.

### Family Periscelididae

Oldenberg

http://species-id.net/wiki/Periscelididae

Periscelidinae Oldenberg 1914: 41 [as a subfamily of Drosophilidae]. Type genus: *Periscelis* Loew, 1858.
Periscelidae . Hendel 1916: 297 [family status].
Periscelididae . Stackelberg 1933: 4 [correct orthography]. Prado 1975: 1–3 [Neotropical catalog]. Mathis and Rung 2011: 359–369 [world catalog].

#### Diagnosis.

*Head*: Frons with 1-2 fronto-orbital setae; postvertical setae present and divergent or absent. Pedicel cap-like and with a dorsal cleft, bearing 1 or more dorsoapical setae; basal flagellomere frequently sharply deflexed, arising from ventral surface of pedicel; arista bipectinate. Face uniformly sclerotized and arched, setose laterally.

*Thorax*: Dorsocentral setae usually 2 (0+2), sometimes 1 (0+1), none presutural; posterior intra-alar seta reduced; scutellum with 1-2 pairs of marginal setae; scutellar disc bare; anepisternal seta usually lacking (present in *Planinasus* and new genus of Stenomicrinae). Wing: subcosta rudimentary, not reaching costa, but not fused apically with vein R_1_; no costal breaks (a weakness in the costa just apicad of the humeral crossvein in *Planinasus*); costa extended to vein R_4+5_ or M; cell dm with a fold running entire length; cell *cup* present, although vein CuA_2_ either well developed or extremely reduced. Midtibia bearing a prominent, apicoventral seta.

#### Discussion.

The concept of Periscelididae, as adopted here, follows D. K. McAlpine (1978, 1983) and includes a few genera previously assigned to Aulacigastridae (*Cyamops* Melander, *Planinasus* Cresson, and *Stenomicra* Coquillett). McAlpine characterized Periscelididae primarily by the caplike pedicel, which has a dorsal cleft, and its relationship to the basal flagellomere. Although these characters are common to all Periscelididae, they also occur in Neurochaetidae (D. K. McAlpine 1978, Woodley 1982) and other Acalyptrate genera. In a recent phylogenetic study of the Opomyzoidea, using 28S ribosomal DNA and CAD (rudimentary) genes (Winkler et al. 2010), *Stenomicra*, *Cyamops* and *Planinasus* grouped consistently with moderate support with the genus *Aulacigaster* and not with Periscelidinae. Moreover, the same analysis failed to find any support for a sister-group relationship between Periscelididae and Neurochaetidae. In an unpublished and comprehensive analysis of the Opomyzoidea, however, the second author found evidence corroborating the proposal of Grimaldi and Mathis (1993), i.e., that the Periscelididae *sensu* D. K. McAlpine (1983) is the sister-group of the Neurochaetidae. The only non-homoplasious synapomorphy supporting this arrangement is the type of articulation between the pedicel and the basal flagellomere. These results highlight the need to study the phylogeny of these groups further and in greater detail.

Papp (1984) proposed Stenomicrinae for the genus *Stenomicra* after D. K. McAlpine (1978, 1983) had transferred that genus, together with *Planinasus* and *Cyamops*, from the Aulacigastridae to the Periscelididae. Grimaldi and Mathis (1993), Baptista and Mathis (1994), Mathis and Papp (1998), and Mathis and Rung (2011) recognized two subfamilies (Periscelidinae and Stenomicrinae) in the Periscelididae, although only the monophyly of Periscelidinae is well corroborated as follows: (1) occiput with a silvery white, microtomentose area immediately adjacent to the posterior margin of the compound eye (secondarily absent in several species); (2) only one fronto-orbital seta, reclinate; (3) mouth opening large (this may be secondarily reduced in *Diopsosoma*, although this is difficult to determine, given the extreme lateral distentions of the head); (4) costa short, extended only to vein R_4+5_; (5) vein CuA_2_ reduced or absent; (6) cell dm with a fold running entire length (also in Stenomicrinae); (7) postpronotal seta well developed; (8) spiracle 7 (“stigma”) not free in female postabdomen; (9) several characters of the male terminalia (see Griffiths 1972). The genera comprising Periscelidinae are those that Hennig (1969) included in his more restricted concept of the family, viz: *Periscelis* Loew, *Marbenia* Malloch, *Neoscutops* Malloch, *Scutops* Coquillett, and *Diopsosoma* Malloch. Baptista and Mathis (1994) questioned the monophyly of Stenomicrinae and presented evidence that *Planinasus* might be more closely related to Periscelidinae. Griffiths (1972) considered *Diopsosoma* Malloch (see also Mathis and Rung 2004) and the small Neotropical genus *Somatia* Schiner (tentatively in a separate subfamily, the Somatiinae) to belong in the Periscelididae. Although Mathis and Papp (1992) and Grimaldi and Mathis (1993) questioned the placement of *Diopsosoma* in the Periscelididae, Mathis and Rung (2004) presented five synapomorphies that confirm its inclusion. Mathis (1993) considered *Somatia* as closely related to the Psilidae (Diopsoinea), while D. K. McAlpine (1997) treated Somatiidae (monotypic) as *incertae sedis*.

#### Key to subfamilies of Periscelididae

##### 

**Table:** 

1	Fronto-orbital seta 1; ocellar setae present. Costa short, extended to vein R_4+5_; vein CuA_2_ weak or lacking, lacking a cell *cup*; postpronotum bearing a well-developed seta	Periscelidinae
–	Fronto-orbital setae 2; ocellar setae absent. Costa long, extended to vein M; vein CuA_2_ usually well developed, usually with a distinct cell *cup* (weak or lacking in *Stenomicra*); postpronotum lacking a well-developed seta	Stenomicrinae

### Subfamily Stenomicrinae

Papp

Stenomicridae Papp 1984: 61 [as the family Stenomicridae]. Type genus: *Stenomicra* Coquillett, 1900. Mathis and Rung 2011: 359–369 [world catalog].

#### Diagnosis. 

*Head*: Frons with 2 fronto-orbital setae, 1 reclinate, usually 1 proclinate; at least 1 vertical seta (apparently the lateral) present; postvertical setae usually lacking (present in some *Stenomicra*, where they are slightly divergent); ocellar setae lacking (a synapomorphy, although present in fossil *Procyamops*). Pedicel bearing 1 or more dorsoapical setae.

*Thorax*: Postpronotum frequently polished, lacking a well-developed seta (a synapomorphy). Wing: No costal breaks, although with weak areas; C extended to vein M; cell *cup* present, CuA_2_ usually well developed (weakly developed or lacking in *Stenomicra*).

#### Discussion.

McAlpine (1978, 1983) transferred the genera of this subfamily (*Planinasus*, *Cyamops*, and *Stenomicra*) to Periscelididae from Aulacigastridae, leaving only *Aulacigaster* Macquart (*Schizochroa* Hennig is considered a junior synonym of *Aulacigaster*) in the subfamily Aulacigastrinae (Rung and Mathis 2011).

The subfamily Stenomicrinae was first proposed as a monogeneric family with *Stenomicra* Coquillett as its type genus (Papp 1984). As we accept McAlpine’s proposal that *Stenomicra* is related to *Planinasus* and *Cyamops* and that this assemblage of genera and those of the subfamily Periscelidinae are likewise related, we prefer recognition of an expanded concept of Periscelididae, with Periscelidinae and Stenomicrinae as included subfamilies.

Although Stenomicrinae are recognized as a subfamily, evidence confirming the monophyly of this subfamily is not compelling nor is its relationship to Periscelidinae (Winkler et al. 2010). Thus, the genera here included in Stenomicrinae may eventually be placed elsewhere in the suprafamily Asteioinea (superfamily Opomyzoidea), which is likewise not well characterized, and these genera may not be as closely related to each other as the classification adopted here would infer.

#### Key to genera of the subfamily Stenomicrinae

##### 

**Table:** 

1	Frons with 1 pair of interfrontal setae; eyes bare. Katepisternum with 2 subequal setae. Hindfemur bearing anterodorsal, preapical seta	*Planinasus* Cresson
–	Frons lacking interfrontal setae; eyes microsetulose, sometimes sparsely. Katepisternum bearing 1 prominent seta. Hindfemur lacking anterodorsal, preapical seta	2
2	Fronto-orbital setae reclinate or occasionally mesoclinate, lacking a proclinate seta; medial vertical seta present with proclinate orientation; face in profile angulate, dorsal surface flattened. Supra-alar seta lacking; lateral scutellar setae 1 pair, apical. Crossvein bm-cu lacking, cells bm and dm confluent; vein CuA_2_ weak or lacking; cell *cup* lacking	*Stenomicra* Coquillett
–	Fronto-orbital setae comprising 1 proclinate and 1 reclinate setae; medial vertical seta absent; face in profile shallowly and vertically arched, lacking a flattened, dorsal area. Supra-alar seta present, well developed; lateral scutellar setae variable but usually 2 pairs (only an apical pair in *Cyamops imitatus* Sturtevant). Crossvein bm-cu well developed, cell bm distinct from dm; vein CuA_2_ present, well developed; cell *cup* present	*Cyamops* Melander

### Genus Planinasus

Cresson

http://species-id.net/wiki/Planinasus

Planinasus Cresson 1914: 245 [in the family Ephydridae; type species: *Planinasus ambiguus* Cresson, by original designation]; Cresson 1918: 65 [discussion, genus probably not in Ephydridae]. Malloch 1934: 52 [generic key, in family Perisceli[di]dae]. Curran 1934: 327 [generic key, in family Drosophilidae]. Hennig 1969: 614–616 [revision, in family Aulacigastridae]. McAlpine 1983: 56 [discussion, assigned to family Periscelididae]. Mathis and Rung 2011: 363 [world catalog].
Schizochaeta Malloch 1934: 52 [type species: *Schizochaeta shannoni* Malloch, by original designation]. Hennig 1969: 614 [synonymy].

#### Diagnosis.

*Head*: Frons with 1 pair of interfrontal setae; reclinate fronto-orbital seta usually smaller than and inserted behind proclinate seta; both medial and lateral vertical setae well developed; postvertical setae absent. Interantennal space at least equal to antennal length, much greater in some species; basal flagellomere arising from anterior surface of pedicel; arista bipectinate. Face uniformly sclerotized and usually arched, bearing a prominent, dorsoclinate, sometimes convergent to cruciate pair of setae near or on transverse facial carina, usually with several other facial setae, these usually ventroclinate and sometimes arranged in a transverse row. Eye bare of interfacetal microsetulae. Genal height less than width of pedicel, lacking a genal seta.

*Thorax*: Dorsocentral setae 2, both postsutural (*Planinasus ambiguus* with a 3rd small, anterior, dorsocentral seta, less than 1/3 length of either posterior 2); supra-alar seta 1; postalar seta 1; postpronotum shiny, lacking a well-developed seta; notopleural setae 2; lateral scutellar setae 1 pair, apical, basal seta lacking; scutellar disc bare; anepisternal setae usually 2, inserted along posterior margin; katepisternal setae 2, anterior seta slightly weaker. Wing: no costal breaks (a weakness in the costa just apicad of the humeral crossvein); costa extended to vein M; subcosta rudimentary, neither reaching costal margin nor fused apically with vein R_1_; vein R_2+3_ minutely but densely trichose on ventral surface; crossvein bm-cu present, with distinct discal cell and cell *bm*; cell *cup* present; discal cell with a fold running entire length; vein CuA_2_ well developed. Legs: forefemur with 1-2 posteroventral setae at apical 1/3; midtibia with apicoventral spine-like seta; hindfemur with anterodorsal preapical seta; all tibiae with subapical dorsal seta.

*Abdomen*: Male: Segments 1–6 with tergites and sternites separate and spiracles 1-6 in membrane; tergite 6 well developed, sternite 6 short and slightly asymmetrical; pregenital segment (sternites 7, 8?) short, immediately adjacent to epandrium, with spiracle 7 within sclerotized portion. Male terminalia as follows: Largely symmetrical; epandrium well developed, bearing numerous setulae; cerci poorly developed, largely unsclerotized, bearing sparse setulae; surstylus usually a long process fused with epandrium, generally, surstyli generally separated from each other (fused medially in *Planinasus ambiguus*); gonostylus relatively simple to complex, often with elaborate processes; pregonite articulated with apex of lateral hypandrial arm; postgonite long, bearing processes and a ventral lobe with setae; subepandrial sclerite rod-like, connecting surstylus with apex of hypandrial arm; aedeagus short; phallapodeme long, narrow; ejaculatory apodeme varying, sometimes dramatically, very large to tiny; hypandrium U or V shaped, often widely so. Female: Spiracle 7 (“stigma”) not free in female postabdomen. Spermathecae 2; ventral receptacle one-chambered.

#### Distribution.

Known only from the New World tropics.

#### Natural History.

Specimens of *Planinasus* are generally rare in collections, and nothing is known about their immature stages, life cycle, or ecology. Their rarity in collections, however, is not necessarily a reflection of their diversity and/or abundance in nature. Specimens are comparatively small, obscure, and could easily be overlooked. We have collected hundreds of specimens of numerous species in the countries of Bolivia, Ecuador , Guyana, Mexico (Chiapas), Peru (Huánuco, Cuzco, Madre de Dios), and on some islands of the Caribbean (Cuba, Dominica, Dominican Republic, Jamaica, and Tobago). In all areas, specimens were collected by sweeping dense, understory vegetation--some bearing flowers--that was associated with shaded, damp, habitats. In Ecuador, we found specimens to be relatively common on the exposed sand or mud substrates in shaded, riparian habitats. Here we observed specimens exposed on the surface of stones or large fallen leaves, perhaps posturing to be seen by conspecifics. We captured numerous specimens alive by simply and carefully lowering a vial over them.

Our field work and sampling, regardless of the collecting technique, also indicates that two to four species frequently occur sympatrically at the same microhabitat. We observed that one species at these sites usually predominates in numbers of individuals. How the various species partition the habitat and what their population structure is are basic questions that remain unanswered.

Grimaldi and Fenster (1989) noted certain preconditions associated with male hypercephaly. Although their list primarily pertains to Drosophilidae, they may also apply elsewhere in Diptera, including species of *Planinasus* that demonstrate hypercephaly. These preconditions, which were manifested at least partially in the few observations we made (see “Mating behavior” under *Planinasus kotrbae*), are: territoriality, face-to-face confrontations, head butting and jousting. Perhaps, like Drosophilidae, there is more aggressiveness among species with hypercephaly than their unmodified relatives. Further observation and comparison are obviously needed, and we hope that this revision will foster such.

#### Discussion.

Several species exhibit considerable sexual dimorphism, especially in the width and coloration of the face. Males in these species tend to have wider faces (hypercephaly), i.e., larger facial ratios, and frequently there is a distinctive colorational pattern. The facial pattern usually also involves microtomentum or its absence in additional to color. These details are included in descriptions of appropriate species.

Within the subfamily Stenomicrinae the sister group of *Planinasus* is apparently either *Cyamops* or *Stenomicra* (Winkler et al. 2010, Mathis and Rung 2011) or perhaps both. The relationship with *Cyamops* is based on the following putative synapomorphies:

1. Midtibia with an apical, anteroventral spine-like seta.

2. Arista bipectinate (McAlpine 1983: 56).

3. Face bearing a dorsoclinate pair of setae, these usually inserted above other facial setae.

*Planinasus* is distinct from other genera of Periscelididae and its monophyly is established by the following putative synapomorphies:

1. Frons bearing a pair of interfrontal setae that are usually slightly reclinate to dorsoclinate. The interfrontal setae, as described, are unique to *Planinasus*.

2. Forefemur with 1-3 posteroventral setae on apical half.

3. Scutellum bearing a single pair of marginal setae, these apical (also in some species of *Cyamops*).

4. Reclinate fronto-orbital seta inserted behind proclinate fronto-orbital seta.

5. Each tibia with a dorsoapical seta.

6. Hindfemur with a subapical dorsal seta.

7. Anepisternum with 1–2 setae along posterior margin (relatively common in other taxa of Asteioinea).

8. Several characters of the male terminalia.

#### Key to extant species of *Planinasus* Cresson

##### 

**Table:** 

1	Large facial setae arranged in a single transverse row of about 8 setae; forefemur bearing 1 large, posteroventral seta at apical 1/3; antenna mostly yellowish orange, pedicel and basal flagellomere with some blackish coloration dorsally	2
–	Large facial setae arranged in 2-3 irregular, transverse rows; forefemur bearing 1-3 subequal, posteroventral setae at apical 1/3; antenna mostly black (base of basal flagellomere pale in males of *Planinasus obscuripennis*)	4
2	Face above transverse carina moderately microtomentose, appearing dull to at most subshiny; tarsomeres of foreleg mostly yellowish, apical tarsomere dark colored; forebasitarsus not compressed	11 *Planinasus insulanus* sp. n.
–	Face above transverse carina sparsely microtomentose to bare, appearing shiny; tarsomeres of foreleg dark colored, mostly blackish, forebasitarsus slightly compressed	3
3	Face with receded, ventral portion short, height less than width of antennal pedicel. Forecoxa mostly whitish yellow; mid- and hindfemora with basal 1/3–1/2 yellowish, contrasted with blackish brown apical portion	12 *Planinasus nigritarsus* sp. n.
–	Face with receded, ventral portion long, height about equal to width of antennal pedicel. Forecoxa black; mid- and hindfemora mostly to entirely blackish brown, at most with basal 1/6 becoming pale	10 *Planinasus argentifacies* sp. n.
4	Interfrontal seta long, at least 2/3 length of lateral vertical seta. Basal flagellomere variable, frequently about twice as long as high	5
–	Interfrontal seta short, about l/2 length of lateral vertical seta. Basal flagellomere short, about as long as high (the *ambiguus* group)	16
5	Ventral projection of pedicel long, length of projection subequal to length of pedicel without considering projection; face of female mostly black, at least dorsally, projected forward, bulbous, evenly arched horizontally, very shallowly arched vertically anterior to rounded, dorsal projection; wing generally strongly infumate (the *shannoni* group)	6
–	Ventral projection of pedicel short, length of projection conspicuously less than length of pedicel without considering projection; face of female generally angulate, dorsal 2/3 more or less evenly slopped anteroventrally to a transversely arched ridge, thereafter nearly vertical to oral margin; wing faintly infumate to completely hyaline	11
6	Antenna of male black (basal flagellomere yellowish basally); face of male with medial portion black, black coloration extended to ventral margin of face, microtomentum sparse or lacking; antennal groove lacking subshiny area extended obliquely medioventrally nearly to oral margin	7
–	At least basal flagellomere, apex of ventral extension of pedicel, and ventral margin of face of male pale colored, mediodorsal area of face densely microtomentose, silvery white; antennal groove with a subshiny, black area extended obliquely medioventrally nearly to oral margin	8
7	Midfacies of male with a conical prominence, shaped like an inverted V with a rounded vertex; face of female with extreme lateral margins yellowish; forefemur of male with basal 2/3 yellow, apical 1/3 black, lacking a preapical ring	4 *Planinasus kotrbae* sp. n.
–	Midfacies of male with a shallowly arched prominence; forefemur of male mostly black, with at most basal 1/4 pale	7 *Planinasus tobagoensis* sp. n.
8	Antenna of male yellow	9 *Planinasus xanthops* sp. n.
–	Scape and pedicel of male mostly brown to brownish black, only basal flagellomere partially to mostly yellowish	9
9	Basal flagellomere of male distinctly two toned, with basal half yellow, apical half black	5 *Planinasus miradorus* sp. n.
–	Basal flagellomere of male yellow, not two toned	10
10	Middle and hind femora mostly yellow, at most with apical 1/3 to 1/4 black	6 *Planinasus shannoni* (Malloch)
–	Middle and hind femora mostly black, at most with basal 1/4 to 1/3 yellowish	8 *Planinasus venezuelensis* Hennig
11	Forecoxa black	12
–	Forecoxa whitish yellow to yellow	14
12	Dorsal, transverse row of facial setae consisting of 1 pair of well-developed, dorsoclinate setae; basal flagellomere of male pale, yellowish on basal 1/2. Wing darkly infumate	18 *Planinasus obscuripennis* sp. n.
–	Dorsal, transverse row of facial setae with some ventroclinate setae in addition to large dorsoclinate setae; antenna of male entirely black. Wing either mostly hyaline or very faintly infumate	13
13	Face bicolored, ventral, receded portion yellow, dorsal portion black; distance between antennal bases of males nearly twice length of antenna (Mexico)	16 *Planinasus mcalpineorum* sp. n.
-	Face black, ventral, receded portion with some silver microtomentum that becomes denser toward oral margin; distance between antennal bases of males about equal to length of antenna (Brazil)	17 *Planinasus nigrifacies* sp. n.
14	Clypeus yellow (Dominica)	15 *Planinasus flavicoxalis* sp. n.
–	Clypeus black	15
15	Parafacial and ventral portion of face adjacent to parafacial yellow (Bolivia (La Paz: Guanay), Brazil (São Paulo: Araçatuba, Córrego Azul))	14 *Planinasus atrifrons* sp. n.
-	Parafacial and ventral portion of face adjacent to parafacial black (Brazil. Rio de Janeiro: Floresta da Tijuca)	13 *Planinasus atriclypeus* sp. n.
16	Surstyli fused medially	2 *Planinasus ambiguus* Cresson
–	Surstyli separate medially	17
17	Surstylus in lateral view straight, parallel sided (Fig. 1)	1 *Planinasus aenigmaticus* sp. n.
–	Surstylus in lateral view swollen basally, thereafter parallel sided (Fig. 15)	3 *Planinasus neotropicus* sp. n.

##### The *ambiguus* group

**Included species.** *Planinasus aenigmaticus* sp. n., *Planinasus ambiguus* Cresson, and *Planinasus neotropicus* sp. n.

**Diagnosis.** This species group is distinguished by the following combination of characters: *Head*: Interfrontal seta short, about half length of lateral vertical seta. Antennal coloration variable; pedicel with short ventral projection; basal flagellomere short, about as high as long. Large facial setae arranged in 2-3 transverse rows; face of males and females similar in shape and color. *Thorax*: Anepisternum with 1 large seta along posterior margin. Wing hyaline to faintly infumate. Forefemur of male lacking subapical, irregular, pale-colored annulus, bearing 2 large seta at apical 1/3 along posteroventral surface. *Abdomen*: Surstylus generally thumb-like, without posterior processes or lobes, in nearly vertical alignment with anterior margin of epandrium; postgonite with robustly developed lobe bearing numerous setulae apically; phallus mostly sclerotized, large, convoluted; ejaculatory apodeme generally well developed, at least as long as phallapodeme, with expanded apex.

**Discussion.** There is little if any dimensional or colorational sexual dimorphism in specimens of this species group. Dimensions and ratios of the heads of both males and females are essentially the same or with broad overlap. Species of this group share with species of the *nigritarsus* group, and only with them, a postgonite with a robustly developed lobe that bears numerous setulae. In other species of *Planinasus*, the lobe of the postgonite, which is generally less developed, bears fewer than four apical setulae, and fewer than six setulae overall. The *ambiguus* and *nigritarsus* groups also share a well-developed ejaculatory apodeme that is at least as long as the phallapodeme (this character state is also present in *Planinasus mcalpineorum* sp. n. of the *nigrifacies* group).

### 1. Planinasus aenigmaticus sp. n.

urn:lsid:zoobank.org:act:707380A1-90E5-417C-88E3-C30704091CF1

http://species-id.net/wiki/Planinasus_aenigmaticus

Figures 1 –5

#### Description.

Moderately small flies, body length 2.70 mm (holotype).

*Head*: Frons mostly bare, shiny, except for more densely microtomentose, anterolateral angles; frons wider than long, frontal ratio 0.50–0.58; interfrontal seta moderately short, about 2/3 length of lateral vertical seta. Antenna unicolorous, blackish brown; pedicel with ventral projection short, not extended anteriorly much beyond dorsal margin; basal flagellomere short, about as long as high, mostly yellow (faintly black around base of arista); arista bearing 8–9 dorsal rays, 3–4 ventral rays. Face comparatively narrow; facial ratio 0.24–0.39; dorsal half of face shield-like, mostly bare, roughly pentagonal, with yellowish to slightly bluish reflections; large facial setae arranged in 2–3 irregular, transverse rows, dorsal row with 4 setae, 2 dorsoclinate, 2 ventroclinate; ventral facial row with 4 ventroclinate setae. Clypeus and palpus blackish brown.

*Thorax*: Generally dark brown but with some paler, mostly yellowish areas along margins of sclerites; mesonotum moderately invested with whitish gray microtomentum, appearing dull medially, becoming subshiny, less microtomentose laterally; postpronotum yellowish; area from postpronotum and through notopleuron mostly bare, shiny; anepisternum moderately invested with very fine microtomentum, mostly appearing dull; other pleural areas less densely invested. Wing mostly hyaline to very faintly infumate, without pattern. Coxae whitish yellow; femora whitish yellow on basal 2/3, only distal 1/3 dark brown; forefemur bearing 1–3 subequal, posteroventral setae at apical 1/3; lacking a preapical annulus; tibiae with base and apex yellow, middle 2/3 brownish black; basitarsomere yellowish, apical 3–4 tarsomeres darker; forefemur bearing 2 setae at apical 1/3 along posteroventral surface.

**Figures 1–4:**
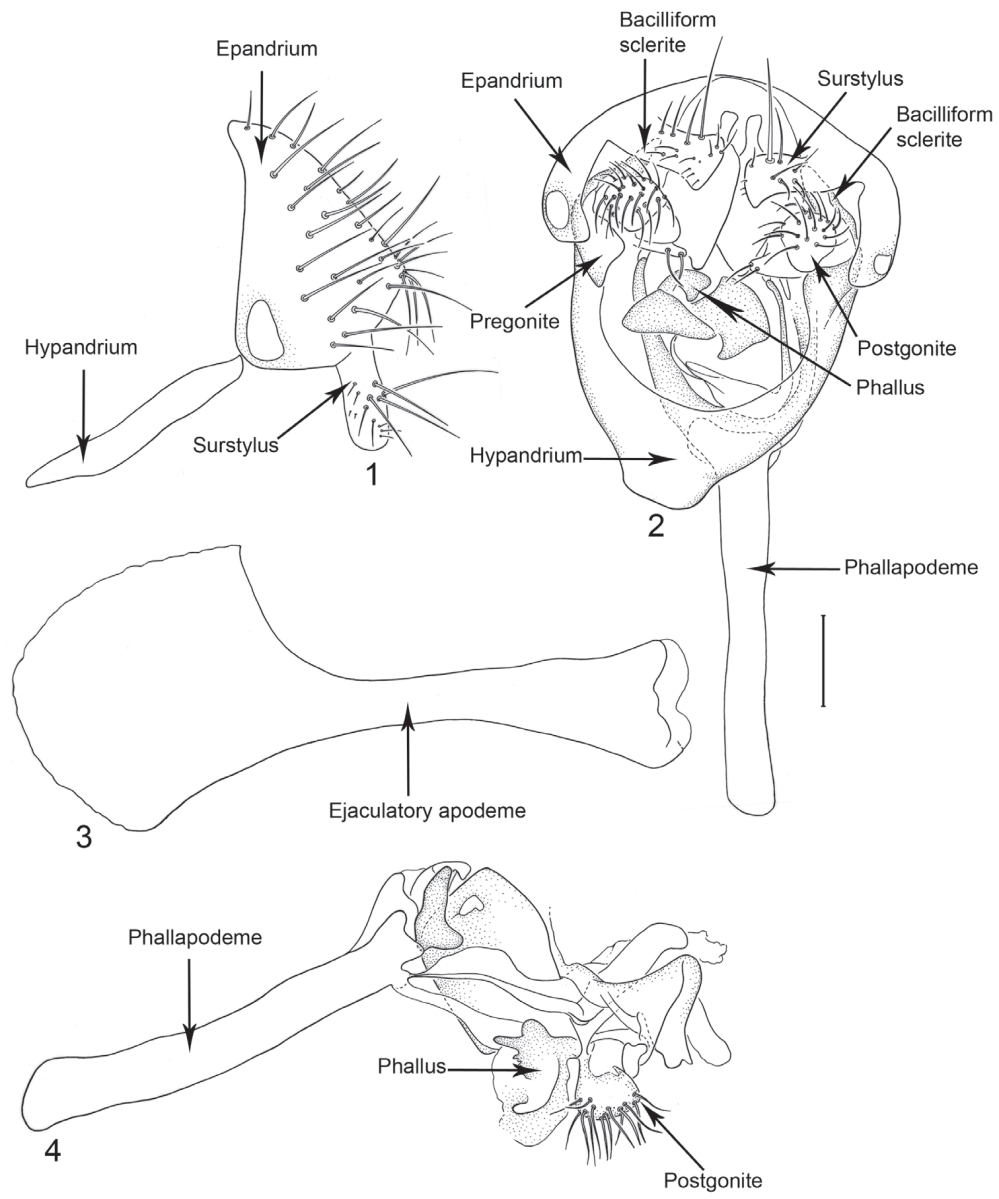
Illustrations of *Planinasus aenigmaticus* sp. n. (male). **1** epandrium, surstylus, hypandrium, lateral view **2** epandrium, hypandrium, and internal structures of male terminalia, ventral view **3** ejaculatory apodeme, lateral view **4** internal structures of male terminalia, lateral view. Scale bar = 0.1 mm.

*Abdomen*: Uniformly blackish brown, mostly subshiny, moderately invested with microtomentum. Male abdomen: Tergites 1+2-6 well developed, lengths of tergites 3–6 subequal; sternite 3 only slightly wider than long, posterior margin sinuous with a medial, broad, rounded projection; width of sternite 4 over twice length, sclerotized portion moderately deeply emarginate with broad, V-shaped membranous area posteriorly; width of sternite 5 over twice length, becoming slightly wider posteriorly, posterior margin shallowly emarginate, bearing 1–2 setulae at posterolateral corners; sternite 6 apparently absent; sternite 7 narrow, forming an annulus with tergite 7. Male terminalia (Figs 1–4): Epandrium in lateral view (Fig. 1) triangular to trapezoidal, dorsal margin narrowly angulate; surstylar length slightly less than half length of epandrium, surstylus extended from ventral margin of epandrium in nearly vertical alignment with anterior margin of it , in lateral and ventral views (Figs 1–2) digitiform, apex bluntly rounded to truncate, length approximately twice width, bearing 4 strong posteromedial setulae and a few smaller setulae along posterior and apical portions; hypandrium in ventral view (Fig. 2) U-shaped with anterior portion of U with a short, trapezoidal projection; pregonite in ventral view (Fig. 2) approximately triangular, with anterior margin receded; postgonite in ventral view convoluted (Fig. 2), apex bearing narrow subrectangular projection, with robustly developed, clavate lobe bearing more than 20 setulae, in lateral view (Fig. 4) with lobe moderately expanded, broadly rounded apically, bearing numerous setulae; phallus large, in ventral and lateral views (Figs 2, 4) complex, mostly sclerotized; phallapodeme in lateral and ventral views (Figs 2, 4) elongate, tubular, truncate apically; ejaculatory apodeme in lateral view (Fig. 3) large, about 1/3 longer than length of phallapodeme , apical half greatly expanded, fan-like, basal half narrow, stem-like but slightly expanded toward base.

**Figure 5.**
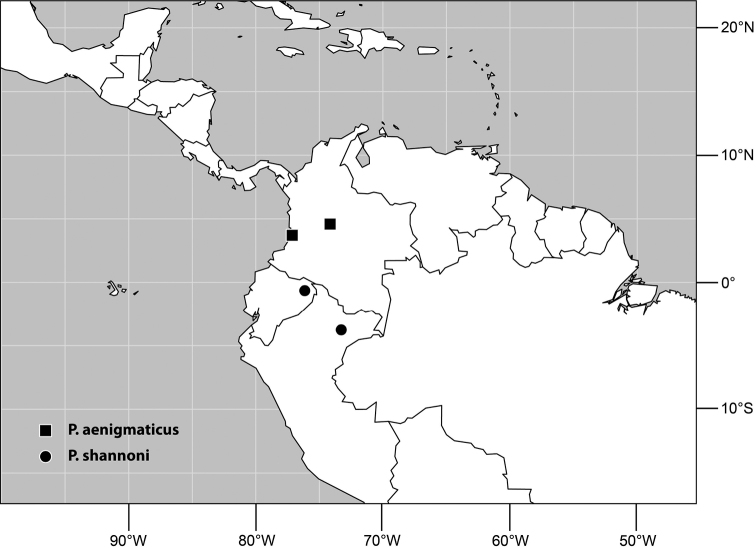
Distribution of *Planinasus aenigmaticus* sp. n. (squares) and *Planinasus shannoni* (Malloch) (dots).

#### Type material.

The holotype male is labeled “W of Bogota COLOMBIA/M R Wheeler collector/USNM ENT 00118284 [plastic bar code label]/**HOLOTYPE** ♂ *Planinasus aenigmaticus* Mathis & Rung USNM [red].” The holotype is double mounted (glued to a paper point), is in good condition (abdomen removed, dissected, stored in an attached microvial), and is deposited in the USNM. A male paratype (CAS) is as follows: COLOMBIA. Valle del Cauca: Río Raposo (03°43'N, 77°08'W), Oct 1964, V. H. Lee.

#### Type locality.

Colombia. Bogota: Bogota (west; 04°35.8'N, 74°08.8'W).

#### Distribution

(Fig. 5). *Neotropical*: Colombia (Bogota, Valle del Cauca).

#### Etymology.

The specific epithet, *aenigmaticus*, is of Greek derivation, meaning something baffling or puzzling, and refers to the puzzling nature of this species, as well as the genus.

#### Remarks.

This species, represented by two males only, is known only from Colombia and is the only species of *Planinasus* known to occur in that country. It is very similar to *Planinasus neotropicus* and *Planinasus ambiguous*, and analysis of the male genitalia is generally necessary to distinguish among the three species. Characters of the male terminalia of *Planinasus ambiguous* are very distinct, particularly the shape of the epandrium (Fig. 10), the length and shape of the surstylus (Fig. 9), and the uniform, U-shaped hypandrium. From *Planinasus neotropicus*, *Planinasus aenigmaticus* can be easily distinguished by the surstylus, digitiform over its entire length and with about four posteromedial setulae (Fig. 1), and the U-shaped hypandrium with the anterior margin bearing a short, trapezoidal projection (Fig. 2). In *Planinasus neotropicus*, the surstylus is bulged basally, there is only one medial seta on it (Fig. 15), and the hypandrium is V-shaped (Fig. 16).

### 2. Planinasus ambiguus

Cresson

http://species-id.net/wiki/Planinasus_ambiguus

**Figures 6–8.**
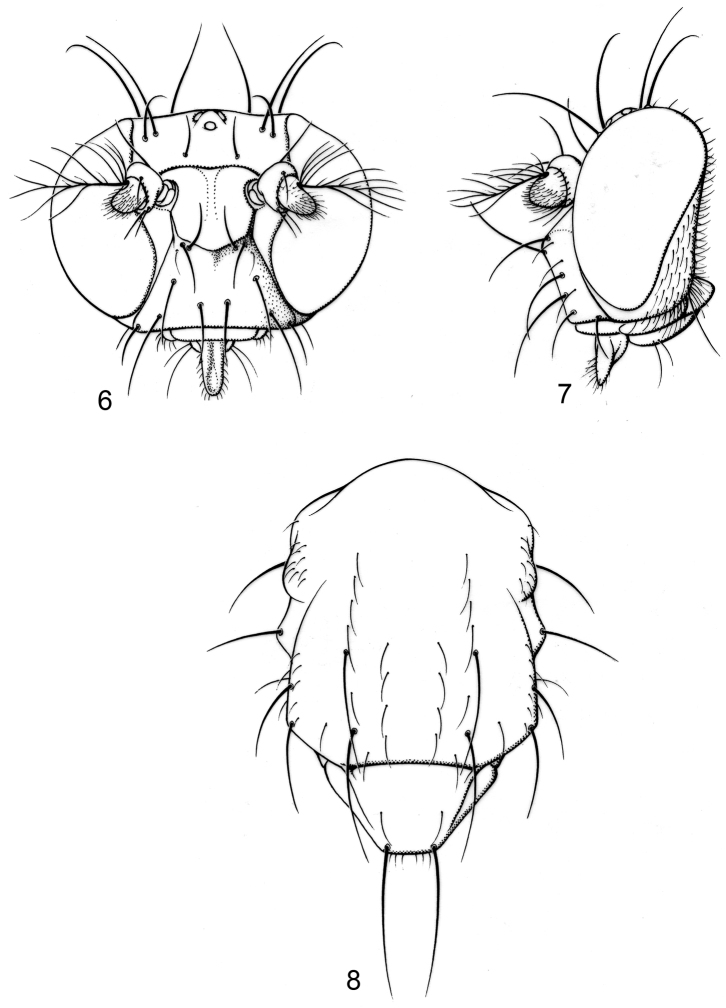
Illustrations of *Planinasus ambiguus* Cresson (male). **6** head, anterior view **7** same, lateral view **8** thorax, dorsal view. Scale bar = 0.1 mm.

Figures 6 [Fig F4] –14

Planinasus ambiguus Cresson 1914: 246. Hennig 1969: 615 [list, Peru]. Mathis and Rung 2011: 363 [world catalog].

#### Description.

Moderately small to medium-sized flies, body length 2.35–3.10 mm.

*Head* (Figs 6–7): Head ratio 0.66–0.73; frons mostly bare, shiny, except for densely microtomentose, velvety-appearing, anterolateral angles; frons wider than long, frontal ratio 0.47-0.51; interfrontal seta short, about 1/2 length of lateral vertical seta. Antenna unicolorous, blackish brown; pedicel with ventral projection short, not extended anteriorly much beyond dorsal margin; basal flagellomere short, height about 2/3 length; arista bearing 8-9 dorsal rays, 3-4 ventral rays. Face comparatively narrow; facial ratio 0.33-0.39; dorsal half of face shield-like, mostly bare, roughly pentagonal, with yellowish to slightly bluish reflections; facial setae more or less in 2-3 transverse rows (usually 2), dorsal row with 4 setae: a medial pair of setae, these approximate, dorsoclinate, sometimes cruciate toward apices; next seta of dorsal row porrect to ventroclinate; middle row with 2 setae (if present), porrect to ventroclinate; ventral facial row with 4 ventroclinate setae. Clypeus and palpus blackish brown.

*Thorax* (Fig. 8): Generally dark brown but with some paler, mostly yellowish areas along margins of sclerites; mesonotum moderately invested with whitish gray microtomentum, appearing dull medially, becoming subshiny, less microtomentose laterally; postpronotum yellowish; area from postpronotum and through notopleuron mostly bare, shiny; anepisternum moderately invested with very fine microtomentum, mostly appearing dull, bearing 1 large seta at posterior margin; other pleural areas less densely invested. Wing very faintly infumate, without pattern. Coxae whitish yellow; femora whitish yellow basally, distal 1/2–1/3 dark brown; forefemur lacking a preapical annulus; basal 1/3 of tibiae dark brown, thereafter gradually becoming yellowish; tarsi yellowish, apical 2–3 tarsomeres darker; forefemur bearing 2 setae at apical 1/3 along posteroventral surface.

**Figures 9–13. F4:**
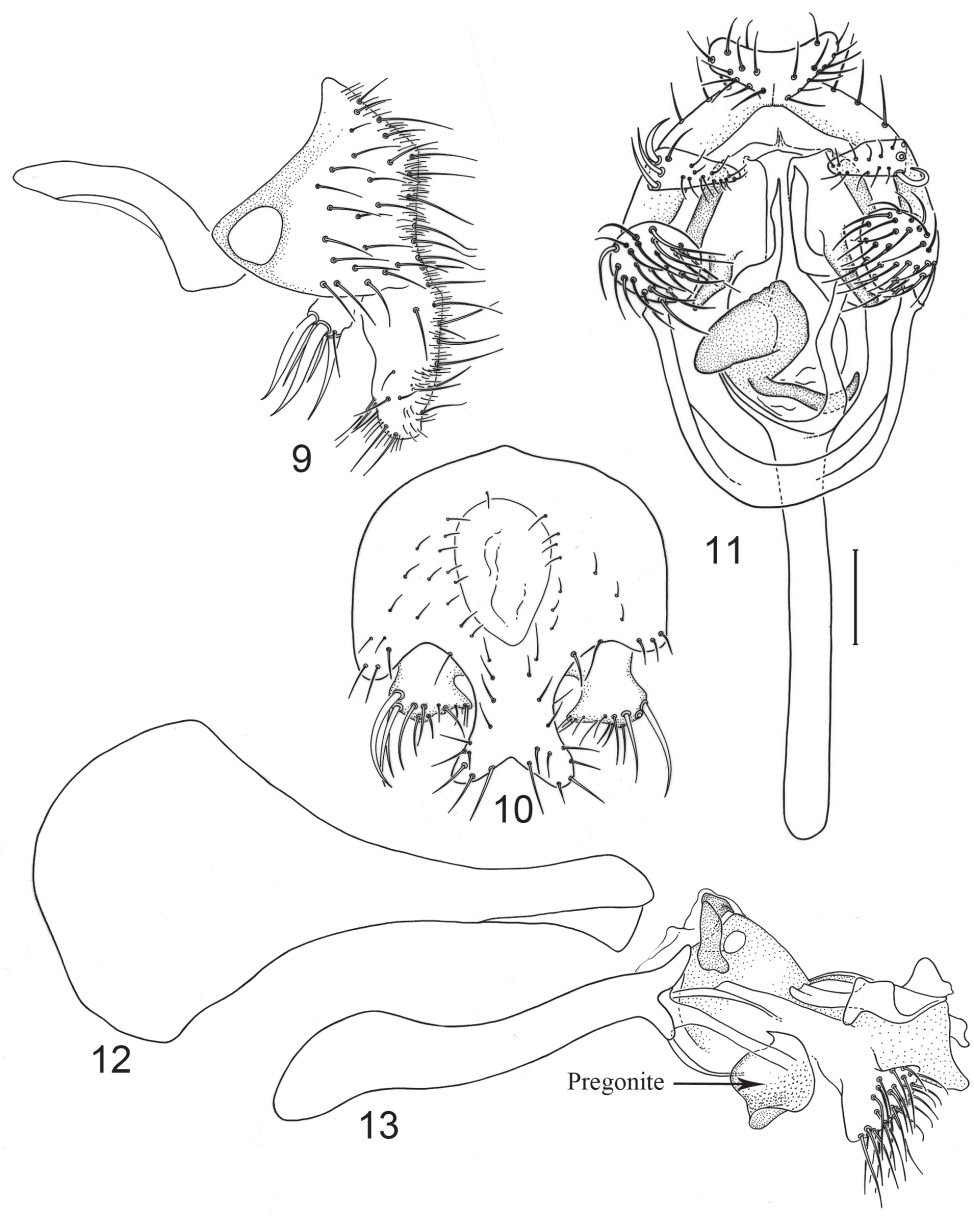
Illustrations of *Planinasus ambiguus* Cresson (male). **9** epandrium, surstylus, hypandrium, lateral view **10** same, posterior view **11** epandrium, hypandrium, and internal structures of male terminalia, ventral view **12** ejaculatory apodeme, lateral view **13** internal structures of male terminalia, lateral view. Scale bar = 0.1 mm.

*Abdomen*: Uniformly blackish brown, mostly subshiny, moderately invested with microtomentum. Male abdomen: Tergites 1+2-6 well developed, lengths of tergites 3-6 subequal; sternite 4 deeply emarginate along posterior margin; sternite 5 with posterior margin moderately deeply emarginate; sternite 6 well sclerotized and developed with width twice length, posterior margin with narrow notch medially, thereafter laterally forming 2 bluntly rounded, wide lobes; sternite 7 narrow, forming an annulus with tergite 7. Male terminalia (Figs 9–13): Epandrium in lateral view (Fig. 9) approximately trapezoidal, dorsal margin truncate, straight, slightly more than half length of shallowly arched ventral margin, in posterior view as a broad, inverted U, cercal cavity oval; surstylus as long as epandrium, extended from ventromedial margin of epandrium in nearly vertical alignment with it, in lateral view (Fig. 9) thumb-like, apex bluntly rounded to truncate, in posterior view (Figs 10) fused medially then bifurcate apically, forming more or less an X, each digitiform, divergent process rounded, length slightly more than width, bearing marginal setulae; hypandrium in ventral view (Fig. 11) U-shaped with anterior portion of mostly uniform width; postgonite in ventral view (Fig. 11) convoluted, with robustly developed lobe expanded apically, clavate, bearing numerous setulae, especially apically, in lateral view (Fig. 13) lobe with greatly expanded apical portion, apex somewhat truncate, bearing more than 20 setulae; phallus large, in ventral view (Fig. 11) complex, mostly sclerotized; phallapodeme in lateral and ventral views (Figs 11, 13) elongate, tubular, sinuous, rounded apically; ejaculatory apodeme in lateral view (Fig. 12) large, about 1/4 longer than length of phallapodeme, apical half greatly expanded, fan-like, basal half narrow, stem-like but slightly expanded toward base.

**Figure 14.**
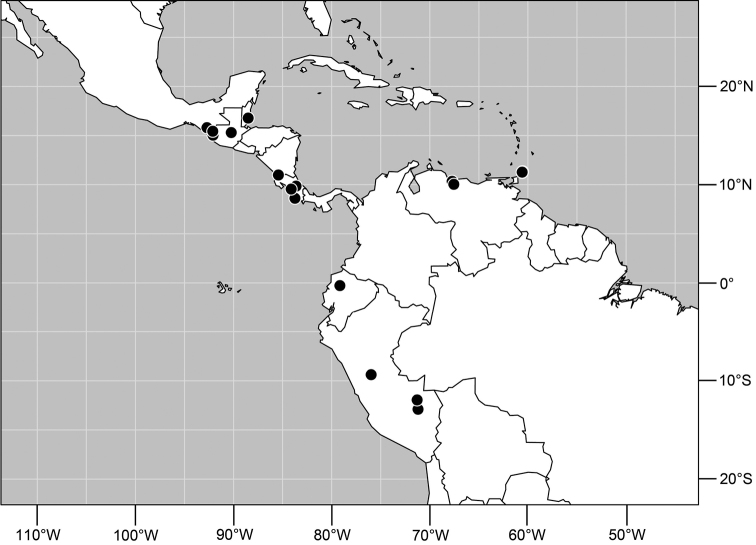
Distribution of *Planinasus ambiguus* Cresson.

#### Type material.

The holotype male is labeled “Cachi C[osta] R[ica][,] 9 III 1910 [9 Mar 1910][,] P P Calvert/Valley of Rio Naranjo/HoloTYPE 6069 [pink]/475 ♂/TYPE No. 6064 [number written across right end of label] Planinasus AMBIGUUS E.T.Cresson,Jr. [pink; species name and number handwritten].” The holotype is double mounted (minuten pin in rectangular card block), is in moderately poor condition (wings glued together), and is deposited in the ANSP (6069).

#### Type locality.

Costa Rica. Cartago: Cachí, Valley of Rio Naranjo (09°49.6'N, 83°48'W).

#### Other specimens examined.

*BELIZE. Stann Creek*: Cockscomb Basin Wildlife Sanctuary (16°47'N, 88°30'W), 5-6 Apr 1993, W. N. Mathis (1♂, 1♀; USNM).

*COSTA RICA. Cartago*: La Suiza (9°51.5'N, 83°37.5'W), 24 Mar, P. Schild (1♂; USNM). *Guanacaste*: Pitilla (9 km S Santa Cecilia; 10°59.5'N, 85°25.8'W; 700 m), Mar 1991, 1994, C. Moraga, P. Ríos (1♂, 2♀; INBIO). *Puntarenas*: San Pedrillo (8°37.2'N, 83°44.1'W), 12–14 Aug 2001, D. and W.N. Mathis (1♀; USNM). *San José*: RioSParaíso (09°33.8'N, 84°7.4'W), 15–17 Feb 2003, W. N. Mathis (21♂, 7♀; USNM).

*ECUADOR. Pichincha*: Santo Domingo de los Colorados, Tinalandia (16 km SE; 0°15.9'S, 79°9.8'W; 680 m), 15–30 May 1975, S. Peck (1♀; CNC).

*GUATEMALA. Alta Verapaz*: Tamahú (15°18.6'N, 90°14.2'W), 25 May 1926, J. M. Aldrich (3♂, 4♀; USNM).

*MEXICO. Chiapas*: Union Juarez (3 km S; 15°02.8'N, 92°05.3'W), 23 Apr 1983, W. N. Mathis (4♂; USNM); Finca Prusia (33 km S Jaltenango; 15°49'N, 92°42'W; 1000 m), 12 May 1985, W. N. Mathis (1♂, 4♀; USNM); Huixtla (32–40.5 km NE; 15°26.3'N, 92°06.8'W), 3 Jun 1969, H. J. Teskey (2♂; CNC).

*PERU. Huánuco*: Las Palmas (1 km N; 09°26.4'S, 75°58'W), 8 Feb 1984, W. N. Mathis (1♂; USNM); Tingo Maria (6 km S; 09°22.7'S, 75°58.5'W), 8 Feb 1984, W. N. Mathis (1♂; USNM). *Madre de Dios*: Río Manu, Erika (near Salvación; 12°50.7'S, 71°23.3'W; 550 m), 5–6 Sep 1988, W. N. Mathis (15♂, 8♀; USNM); Río Manu, Pakitza (11°56.6'S, 71°16.9'W; 250 m), 9–23 Sep 1988, A. Freidberg, W. N. Mathis (2♀; USNM).

*TRINIDAD and TOBAGO. Tobago. St. John*: Charlottville (2 km S, water treatment plant; 11°19'N, 60°33'W), 10 Jun 1993, W. N. Mathis (1♂, 1♀; USNM); Parlatuvier (creek; 11°17.9'N, 60°35'W), 20 Apr 1994, W. N. Mathis (12♂, 10♀; USNM). *St. Paul*: Argyle Falls (11°15'N, 60°35'W), 21 Apr 1994, W. N. Mathis (1♂; USNM); Roxborough (6.5 km N; 11°17'N, 60°35'W), 14 Jun 1993, W. N. Mathis (1♂; USNM).

*VENEZUELA. Aragua*: H. Pittier Nacional Parque (6 km N Rancho Grande Biological Station; 10°21.2'N, 67°42.9'W; along stream), 28 Feb 1995, S. A. Marshall (1♂; DEBU); Portachuelo Pass (10°02.7'N, 67°33'W), 16 Aug 1967, R. W. Poole (1♂; USNM).

#### Distribution

(Fig. 14). *Neotropical*: Belize, Costa Rica (Cartago, Guanacaste, Puntarenas, San José), Ecuador (Pichincha), Guatemala, Mexico (Chiapas), Peru (Huánuco, Madre de Dios), Trinidad and Tobago, and Venezuela (Aragua).

#### Remarks.

This widespread species was previously known only from Costa Rica and Peru (Hennig 1969) and is here recorded from as far north as Chiapas, Mexico, and to the south as far as Peru (Madre de Dios). This species can be easily confounded with the other two species in the *ambiguus* group based on external characters, but characters of the male terminalia of *Planinasus ambiguus* unambiguously diagnose this species. See “Remarks” under *Planinasus aenigmaticus* sp. n. for further discussion.

### 3. Planinasus neotropicus sp. n.

urn:lsid:zoobank.org:act:061DBE88-5E6E-4850-9D95-C22038BD31C6

http://species-id.net/wiki/Planinasus_neotropicus

Figures 15 –19

#### Description.

Small to moderately small flies, body length 1.80–2.55 mm.

*Head*: Frons mostly bare, shiny, except for densely microtomentose, velvety-appearing, anterolateral angles; frons slightly wider than long, frontal ratio averaging 0.65; interfrontal seta short, about 1/2 length of lateral vertical seta. Antenna unicolorous, blackish brown; pedicel with ventral projection short, not extended anteriorly much beyond dorsal margin; basal flagellomere short, width about 2/3 length; arista bearing 8-9 dorsal rays, 3-4 ventral rays. Face comparatively narrow; facial ratio averaging 0.25; dorsal half of face shield-like, mostly bare, roughly pentagonal, with yellowish to slightly bluish reflections; facial setae more or less in 2 transverse rows, dorsal row with 4 setae, 2 dorsoclinate, 2 ventroclinate; ventral facial row with 4 ventroclinate setae. Clypeus and palpus blackish brown.

*Thorax*: Generally dark brown but with some paler, mostly yellowish areas along margins of sclerites; mesonotum moderately invested with whitish gray microtomentum, appearing dull medially, becoming subshiny, less microtomentose laterally; postpronotum yellowish; area from postpronotum and through notopleuron mostly bare, shiny; anepisternum moderately invested with very fine microtomentum, mostly appearing dull; other pleural areas less densely invested. Wing very faintly infumate, without pattern. Coxae whitish yellow; femora whitish yellow basally, distal 1/2–1/3 dark brown; forefemur lacking a preapical annulus; basal 1/3 of tibiae dark brown, thereafter gradually becoming yellowish; tarsi yellowish, apical 2-3 tarsomeres darker; forefemur bearing 2 setae at apical 1/3 along posteroventral surface.

**Figures 15–18.**
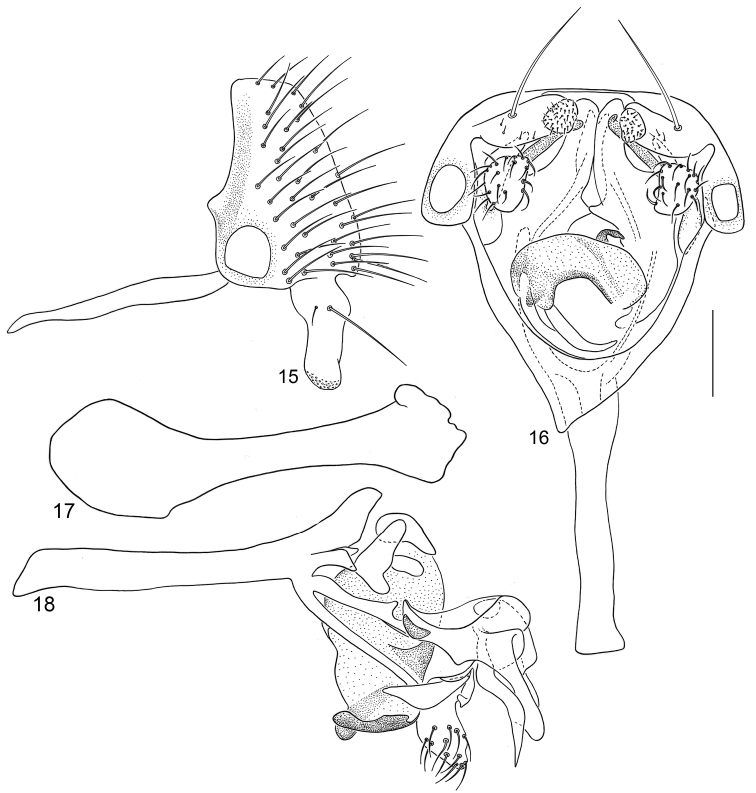
Illustrations of *Planinasus neotropicus* sp. n. (male). **15** epandrium, surstylus, hypandrium, lateral view **16** structures of internal male terminalia, ventral view **17** ejaculatory apodeme, lateral view **18** structures of internal male terminalia, lateral view. Scale bar = 0.1 mm.

*Abdomen*: Uniformly blackish brown, mostly subshiny, moderately invested with microtomentum. Male abdomen: Tergites 1+2-6 well developed, lengths of tergites 3-6 subequal; sternite 3 only slightly wider than long, posterior margin sinuous with a medial, moderately broad, rounded projection; sternite 4 with width over twice length, sclerotized portion deeply emarginate, widely W-shaped with broad portion membranous posteriorly; sternite 5 with width over twice length, anterior margin with shallow, medial depression, lateral margins becoming slighter wider posteriorly, posterior margin shallowly emarginate, posterolateral corners rounded; sternite 6 apparently absent; sternite 7 well developed but narrow, forming an annulus with tergite 7. Male terminalia (Figs 15–18): Epandrium in lateral view (Fig. 15) narrowly trapezoidal, dorsal margin truncate, straight; surstylar length slightly less than half length of epandrium, extended from ventral margin of epandrium, in nearly vertical alignment with epandrium, in lateral view (Figs 15–16) digitiform on apical 2/3, with anterior and posterior basal margins bulged, apex bluntly rounded to truncate, bearing 1 large, sub-basal setula; hypandrium in ventral view (Fig. 16) V-shaped with anterior, angulate portion of V thickened; postgonite in ventral view (Fig. 16) convoluted, with robustly developed lobe expanded apically, invested with numerous setulae, lobe in lateral view (Fig. 18) irregularly clavate with uneven expansion, apical portion bearing approximately 20 setulae; phallus large, in ventral view (Fig. 16) complex, partially sclerotized; phallapodeme in lateral and ventral views (Figs 16, 18) elongate, tubular, truncate apically; ejaculatory apodeme in lateral view (Fig. 17) long, length subequal to that of phallapodeme, clavate, apex only moderately expanded.

**Figure 19.**
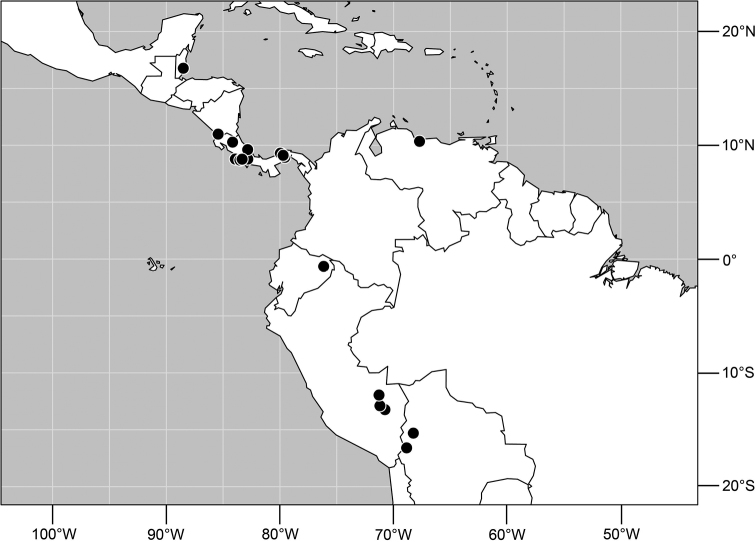
Distribution of *Planinasus neotropicus* sp. n.

#### Type material.

The holotype male is labeled “*PANAMA*: CANAL ZONE Barro Colorado Isl. 28. June. [day and month handwritten] 1978 N.E.Woodley/Malaise trap/USNM ENT 00118285 [plastic bar code label]/**HOLOTYPE** ♂ *Planinasus neotropicus* Mathis & Rung USNM [red].” The holotype is double mounted (minuten wired to a larger pin), is in good condition (some setae missing), and is deposited in the USNM. Paratypes are as follows: *PANAMA*. Balboa (08°57'N, 79°34'W), Feb 1958, M. R. Wheeler (1♂, 1♀; USNM). Fort Davis (09°17.3'N, 79°54.4'W), 28 Aug 1952, F. S. Blanton (1♀; USNM). Fort Sherman, Mojinga Swamp (09°18.2'N, 79°57.9'W), 30 Jan 1952, F. S. Blanton (1♂; USNM). Gamboa, Rio Agua Salud (09°07.1'N, 79°41.6'W), Jul 1967, W. W. Wirth (1♂; USNM). Las Cruces Trail (09°07.3'N, 79°42.7'W), Feb 1958, M. R. Wheeler (3♂, 2♀; USNM). Monte Lirio (08°57.5'N, 82°49.5'W), 6 Apr 1923, R. C. Shannon (1♀; USNM).

**Type locality.** Panama. Canal Zone: Barro Colorado Island (09°09.1'N, 79°50.8'W).

**Other specimens examined.** *BELIZE*. Stann Creek: Cockscomb Basin Wildlife Sanctuary (16°47'N, 88°30'W), 5–6 Apr 1993, W. N. Mathis (3♂; USNM).

*BOLIVIA. La Paz*: Apa Apa (8 km S Chulumani; 16°35.6'S, 68°51.2'W; 3840 m), 10 Mar 2001, W. N. Mathis (1♀; USNM); Mapiri (15°18.6'S, 68°13'W; 720 m), 15 Mar 2001, W. N. Mathis (1♂; USNM); Mapiri (5 km W; Arroyo Tuhiri; 15°17.8'S, 68°15.6'W; 750 m), 16 Mar 2001, A. Freidberg, W. N. Mathis (16♂, 10♀; USNM).

*COSTA RICA. Cartago*: Tapanti National Park (08°47.7'N, 83°55.6'W; wet log over stream), 11 Oct 1999, S. A. Marshall (2♂, 2♀; DEBU). *Guanacaste*: Pitilla (9 km S Santa Cecilia; 10°59.5'N, 85°25.8'W; 700 m), 21 Feb 1996, S. A. Marshall (1♂; DEBU). *Heredia*: La Virgen del Socorro (10°16.8'N, 84°10'W; 700 m; swept over wet leaf litter), 17 Mar 1991, D. A. Grimaldi, J. Stark (4♂; AMNH). *Limón*: Bribri (4 km NE; 09°37.5'N, 82°49.8'W; 50 m), Dec-Mar, 1989, 1990, P. Hanson (1♂; DEBU). *Puntarenas*: Peninsula de Osa, Rincón (08°42.5'N, 83°29.2'W; palm-mangrove forest; sea level), 24 Mar 1991, D. A. Grimaldi, J. Stark (3♂, 4♀; AMNH); Rincón (5 km S; 08°42.1'N, 83°30.8'W; 95 m), 10–11 Aug 2001, D. and W. N. Mathis (2♂, 7♀; USNM); R. F. Golfo Dulce (24 km W Piedras Blancas; 08°47.2'N, 83°19.3'W; 200 m), Nov 1990, P. Hanson (2♀; DEBU).

*ECUADOR. Orellana*: Rio Tiputini Biodiversity Station (0°38.2'S, 76°8.9'W), 12–26 Aug 1999, A. Baptista, M. Kotrba, W. N. Mathis (1♂, 16♀; USNM, ZSMC).

*PERU. Cuzco*: Quincemil (13°13.7'S, 70°45.6'W; 740 m), Aug 1962, L. E. Peña (2♂, 3♀; CNC). *Madre de Dios*: Río Manu, Pakitza (11°56.6'S, 71°16.9'W; 250 m), 9–23 Sep 1988, A. Freidberg, W. N. Mathis (17♂, 11♀; USNM); Río Manu, Erika (near Salvación; 12°50.7'S, 71°23.3'W; 550 m), 5–6 Sep 1988, A. Freidberg (2♂, 2♀; USNM).

*VENEZUELA. Aragua*: H. Pittier National Park (6 km N Rancho Grande Biological Station; 10°21.2'N, 67°42.9'W; along stream), 28 Feb 1995, S. A. Marshall (2♂; DEBU).

#### Distribution

(Fig. 19). *Neotropical*: Belize, Bolivia (La Paz), Costa Rica (Cartago, Guanacaste, Heredia, Limón, Puntarenas), Ecuador (Orellana), Panama, and Peru (Cuzco, Madre de Dios), Venezuela (Aragua).

#### Etymology.

The species epithet, *neotropicus*, is to recognize the Neotropical distribution of this species and is a noun in apposition.

#### Remarks.

This species, like *Planinasus ambiguus*, is relatively widespread, especially in Andean countries. It can be easily confounded with the other two species in the *ambiguus* group based on external characters. See “Remarks” under *Planinasus aenigmaticus* sp. n. for further discussion on how to distinguish among *Planinasus aenigmaticus* sp. n., *Planinasus ambiguus* and *Planinasus neotropicus* sp. n.

##### The *shannoni* group

**Included species.** *Planinasus kotrbae* sp. n., *Planinasus miradorus* sp. n., *Planinasus shannoni* (Malloch), *Planinasus tobagoensis* sp. n., *Planinasus venezuelensis* Hennig, and *Planinasus xanthops* sp. n.

**Diagnosis.** This species group is distinguished by the following combination of characters: *Head*: Interfrontal seta long, subequal to length of lateral vertical seta. Antennal coloration variable; pedicel with elongate ventral projection; basal flagellomere long, length nearly twice height. Large facial setae arranged in 2-3 transverse rows; face of females bulbous, evenly transversely arched, mostly black. *Thorax*: Anepisternum with 2-4 weak setae along posterior margin. Wing strongly infumate. Forefemur of male lacking a preapical yellowish annulus, bearing 1 large, posteroventral seta at apical 1/3. *Abdomen*: Surstylus generally without posterior processes or lobes (*Planinasus kotrbae* has a process), in nearly oblique alignment with anterior margin of epandrium (except in *Planinasus tobagoensis*); postgonite with small, finger-like lobe bearing a few apical setulae; phallus mostly membranous (more heavily sclerotized in *Planinasus shannoni*); ejaculatory apodeme generally reduced, inconspicuous in some species.

**Discussion.** The species of this group demonstrate considerable sexual dimorphism. The dimorphism is most evident in the shape and coloration of the face. The shape and coloration of the male face varies, depending on the species, but the face of females is quite similar in three species and is generally dark colored and conspicuously projected forward, bulbous, evenly arched transversely, somewhat flat in lateral view on ventral half. Species of this group share, with species of the *atriclypeus* group and with *Planinasus obscuripennis* sp. n. (the *obscuripennis* group) the reduced number of setae on the postgonite (2-6), and the ejaculatory apodeme is generally reduced or inconspicuous. In other species of *Planinasus*, the lobe of the postgonite, which is generally better developed, bears more than 20 setulae, and the ejaculatory apodeme is large, with a fan-like apical expansion.

### 4. Planinasus kotrbae sp. n.

urn:lsid:zoobank.org:act:9D270B5D-D4DC-4E86-806F-638A791337D2

http://species-id.net/wiki/Planinasus_kotrbae

**Figures 20–23.**
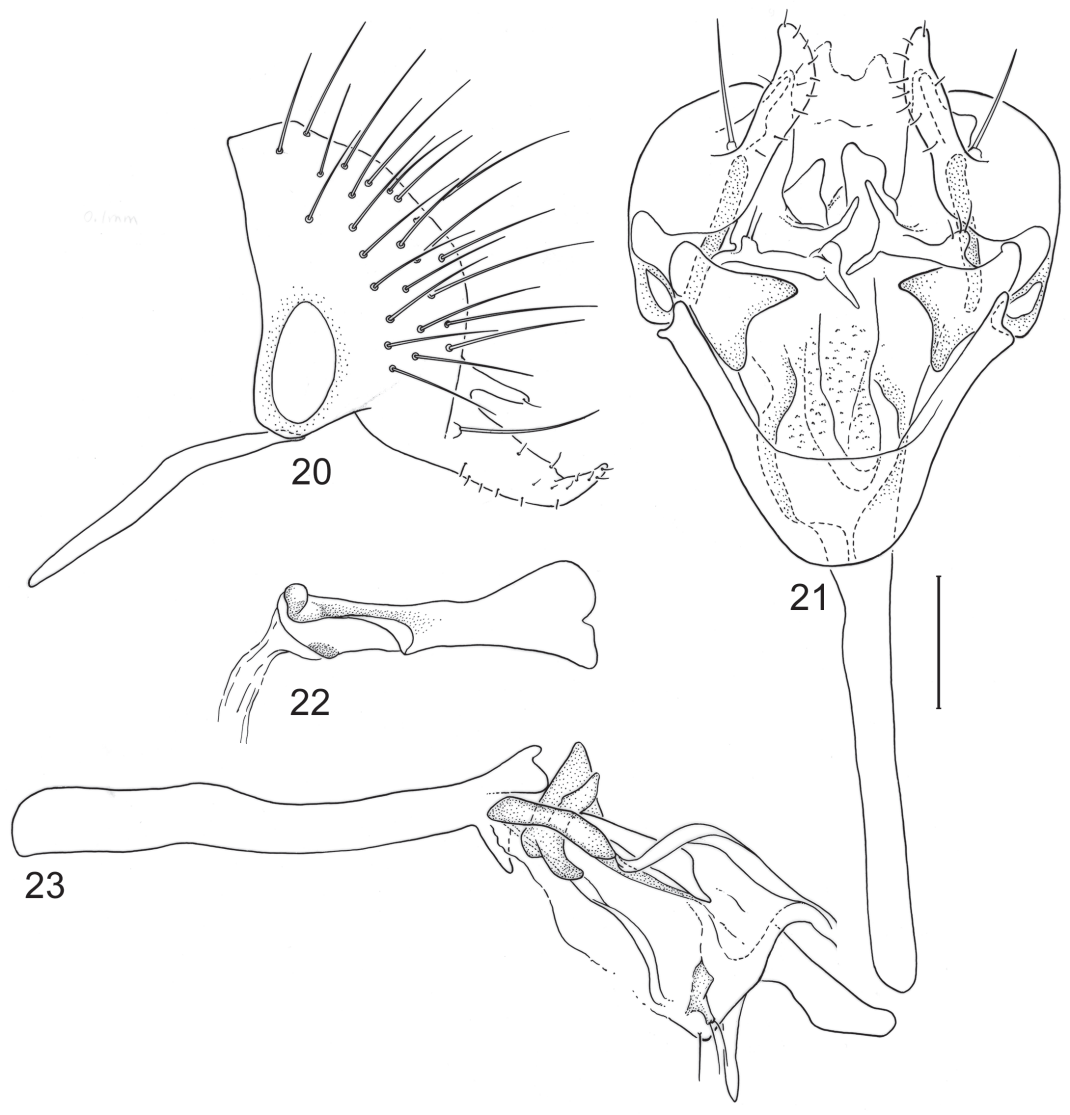
Illustrations of *Planinasus kotrbae* sp. n. (male). **20** epandrium, surstylus, hypandrium, lateral view **21** internal structures of male terminalia, ventral view **22** ejaculatory apodeme, lateral view **23** internal structures of male terminalia, lateral view. Scale bar = 0.1 mm.

Figures 20 [Fig F9] [Fig F10] –29

#### Description of male.

Moderately small to medium-sized flies, body length 2.50–3.70 mm.

*Head*: Frons mostly bare of microtomentum, shiny, except for densely microtomentose, velvety-appearing, anterolateral corners, blackish brown; frons much wider than long, frontal ratio averaging 0.48; interfrontal seta long, length subequal to that of lateral vertical seta. Antenna generally blackish brown, basal flagellomere partially yellowish basally; pedicel with ventral projection long, about 1/2 length of basal flagellomere; basal flagellomere long, slightly more than twice basal width; arista bearing 13-14 dorsal rays, 3-4 ventral rays. Face very wide, facial ratio averaging 0.96; face mostly black, black coloration extended to ventral margin of face, lateral margin just ventrad of antennal groove whitish yellow, concolorous with coloration of adjacent parafacial; facial microtomentum sparse or lacking, mostly shiny; midfacies with a conical prominence, shaped like an inverted V; antennal grooves shiny, lacking narrowly triangular, bare area extended medioventrally from ventral margin of antennal groove to oral margin; large facial setae 4-5, not arranged in transverse rows, becoming smaller ventrally; largest seta inserted dorsolaterally, dorsoclinate and slightly convergent; 2nd largest seta inserted medioventrally from largest seta, porrect and parallel; sometimes with a smaller seta inserted just above porrect seta; 2 setae inserted at margin of black and yellowish white color, these oriented ventrally. Clypeus and palpus blackish brown. Gena concolorous with lateral margin of face.

*Thorax*: Generally dark colored, black to blackish brown, anepimeron paler, brownish, with ventral margin yellowish; mesonotum thinly invested with microtomentum, appearing subshiny, with slightly metallic brown luster medially, becoming more steel blue laterally; postpronotum dark brown; area from postpronotum and extended through notopleuron mostly bare, shiny; anepisternum thinly invested with microtomentum, mostly appearing dull, grayish brown; other pleurites less densely invested. Wing conspicuously and uniformly infumate, brownish, base slightly paler, more hyaline. Coxae yellowish; femora mostly yellowish, only apex blackish (apical 1/3 of forefemur, apical 1/8 of middle and hind femora); forefemur lacking a preapical yellowish annulus; tibiae blackish brown; tarsi, except apical 1-2 dark brown tarsomeres, yellowish. Forefemur with posteroventral surface bearing 1 large seta at apical 1/3.

*Abdomen*: Uniformly blackish brown to black, mostly shiny, very sparsely invested with microtomentum. Male abdomen: Tergites 1+2-6 well developed, lengths of tergites 3-6 subequal; tergite 7 narrow; sternites 3, 4, 5 generally as rectangular plates, slightly wider than long, lateral margins shallowly arched; sternite 5 slightly wider subposteriorly; no sternites 6, 7, neither segment forming an annulus. Male terminalia (Figs 20–23): Epandrium in lateral view (Fig. 20) higher than wide, anterior margin nearly straight, posterior margin arched, dorsal margin short, length slightly more than width of surstylus, truncate; surstylus almost as long as epandrium, extended from ventroposterior margin of epandrium in nearly oblique alignment with it , in lateral view (Fig. 20) slipper shaped, tapered toward apex, apex curved dorsally, acutely pointed, with a second, dorsal, slender, digitiform process from base, bearing 1 large, basal setula; hypandrium in ventral view (Fig. 21) more or less V-shaped, arms more slender than thickened base, anterior margin rounded; pregonite in ventral view (Fig. 21) approximately triangular, with anterior margin receded and with slightly round corners; postgonite in ventral view (Fig. 21) convoluted, with a lobe about as wide as long, bearing a few apical setulae, lobe in lateral view (Fig. 23) small, bearing two apical setulae; phallus in ventral view (Fig. 21) complex, only partially sclerotized; phallapodeme in lateral and ventral views (Figs 21, 23) elongate, tubular, nearly straight, very shallowly sinuous, bluntly rounded apically; ejaculatory apodeme much reduced, a little longer than half of length of phallapodeme, in lateral view (Fig. 22) expanded apically, lacking fan-like process.

#### Description of female.

Same as male except as follows: *Head*: Frontal ratio averaging 0.55; face and antenna mostly blackish brown, lateral margin of face (immediately adjacent to parafacial) whitish yellow; face not as wide, facial ratio averaging 0.42; face projected forward on ventral 1/2, bulbous, evenly arched transversely, shallowly arched vertically, mostly flat.

**Figure 24. F9:**
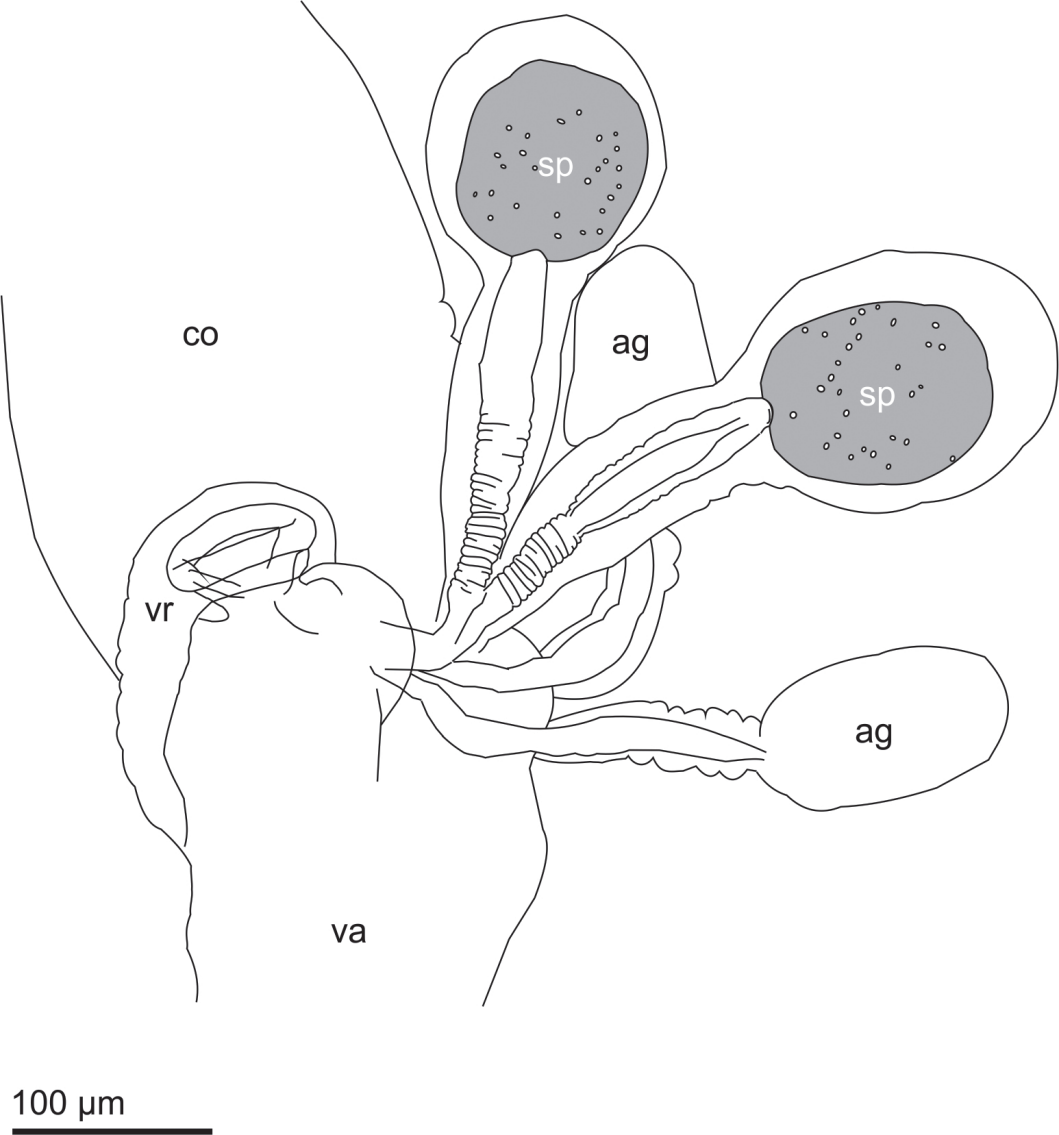
Internal female reproductive organs of *Planinasus kotrbae* sp. n., schematic drawing, left lateral view, ovaries and lateral oviducts omitted. **ag** accessory gland **co** common oviduct **sp** spermatheca **va** vagina **vr** ventral receptacle.

**Figures 25–28. F10:**
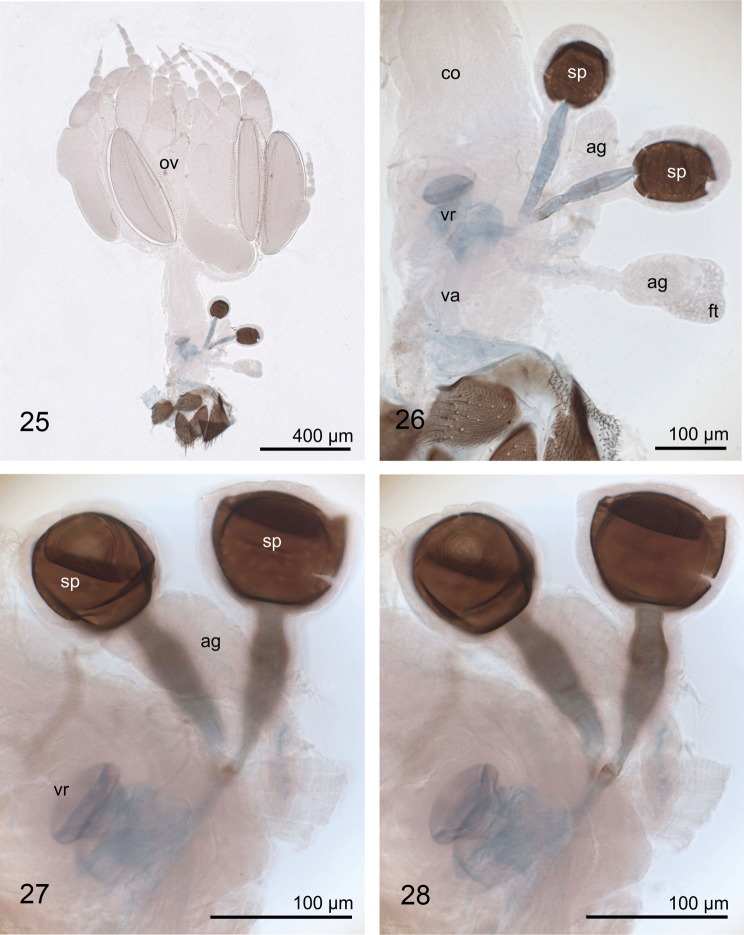
Internal female reproductive organs of Planinasus kotrbae sp. n., photos, left lateral view. **25** overview **26** vagina and associated organs **27** ventral receptacle and spermathecae **28** same, different focal plane. **ag** accessory gland **co** common oviduct **ft** fatty tissue **ov** ovaries **sp** spermatheca, slightly cracked and distorted in the microscopic slide preparation **va** vagina **vr** ventral receptacle.

*Abdomen*: Internal female reproductive tract (Figs 24–28): Common oviduct opening anterodorsally into vagina. Posterior to this, paired spermathecae and accessory glands opening adjacent to each other into dorsal vaginal wall. Each spermatheca consisting of ovoid dark brown chamber with shallow circular depression apically; its sclerotized wall slightly wrinkled with tiny dimples; no basal introvert present. Spermathecal ducts lined by thick cuticle with more or less distinct annulation, sometimes slightly sclerotized at their base. Accessory glands about as long as spermathecae; their gland reservoirs and ducts lined by membranous cuticle. Ventral receptacle colourless, one-chambered, arising from anteroventral portion of vagina, round with circular apical depression, thus resembling (an inverted toilet plunger or) a shallow double-walled bowl.

**Figure 29.**
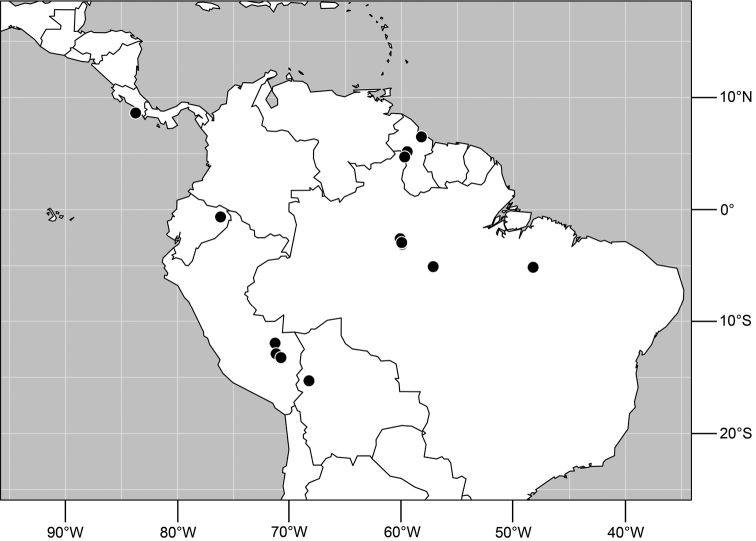
Distribution of *Planinasus kotrbae* sp. n.

#### Type material.

The holotype male is labeled “ECUADOR. Prt. Or[e]ll[a]na: RioTiputini [Biodiversity Station] (0°38.2'S, 76°8.9'W)[,] 12–26Aug 1999,W.N.Mathis, A. Baptista, M. Kotrba/USNM ENT 00118275 [plastic bar code label]/**HOLOTYPE** ♂ *Planinasus kotrbae* Mathis & Rung USNM [red].” The holotype is double mounted (minuten pin in plastic block), is in excellent condition, and is deposited at the USNM. Forty-eight paratypes (30♂, 18♀; USNM, ZSMC) bear the same label data as the holotype. Other paratypes are as follows: *PERU. Madre de Dios*: Río Manu, Pakitza (11°56.6'S, 71°16.9'W; 250 m), 9–23 Sep 1988, A. Freidberg, W. N. Mathis (14♂, 20♀; USNM); Río Manu, Erika (near Salvación; 12°50.7'S, 71°23.3'W; 550 m), 5–6 Sep 1988, A. Freidberg (3♂, 2♀; USNM). *Cuzco*: Quincemil (13°13.7'S, 70°45.6'W; 740 m), 13–31 Aug 1962, L. E. Peña (1♂; CNC).

#### Type locality.

Ecuador. Orellana: Rio Tiputini Biodiversity Station (0°38.2'S, 76°08.9'W).

#### Other specimens examined.

*BOLIVIA. La Paz*: Mapiri (5 km west; 15°17.8'S, 68°15.6'W; 750 m), 16 Mar 2001, W. N. Mathis (1♂; USNM).

*BRAZIL. Amazonas*: Carauari (05°04.5'S, 57°10.2'W), Jul 2005, A. Henriques (1♀; INPA); Manaus, UFAM (03°05.9'S, 59°58.2'W; 50 m), 7 May 2010, D. and W. N. Mathis (3♂; INPA, USNM); Reserva Cuieiras (02°35.2'S, 60°07.2'W; 110 m), 8 May 2010, D. and W. N. Mathis (1♂; USNM); Reserva Florestal Adolpho Ducke (02°55.8'S, 59°58.5'W; 40 m), 5 May 2010, D. and W. N. Mathis (2♂; INPA, USNM); Reserva Florestal Adolpho Ducke, Igarapé Barro Branco (02°58.1'S, 60°0.3'W; 40 m; Malaise trap), A. Henriques (1♂; INPA); Reserva Florestal Adolpho Ducke, Igarapé Tinga (02°55.8'S, 59°58.5'W; suspension trap at 25 m), Mar 2004, A. Henriques (1♀; INPA). *Maranhão*: São Pedro da Água Branca, Fazenda Santa Rosa (05°07.8'S, 48°15.2'W), 6 Dec 2001, F. L. Oliveira, J. A. Rafael, J. Vidal (1♀; INPA).

*COSTA RICA. Puntarenas*: San Pedrillo (8°37.2'N, 83°44.1'W), 12–14 Aug 2001, D. and W.N. Mathis (8♀; USNM).

*GUYANA*. Conservation of Ecological Interactions and Biotic Associations (CEIBA; ca. 40 km S Georgetown; 06°29.9'N, 58°13.1'W), 13–21 Apr-28 Aug 1994, 1995, 1997, W. N. Mathis (47♂, 12♀; USNM). Menzies Landing (05°10.1'N, 59°29.5'W), 23 Aug 1997, W. N. Mathis (3♂, 1♀; USNM). Paramakatoi (04°42'N, 59°42.8'W), 24–25 Aug 1997, W. N. Mathis (1♂, 1♀; USNM).

#### Distribution

(Fig. 29). *Neotropical*: Bolivia (La Paz), Brazil (Amazonas, Maranhão), Costa Rica (Puntarenas), Ecuador (Orellana), Guyana, and Peru (Cuzco, Madre de Dios).

#### Etymology.

The specific epithet, *kotrbae*, is Latin genitive patronym to honor Dr. Marion Kotrba (ZSMC), who conducted field work with us in Ecuador and allowed us to use her rearing cage for making observations on the mating behavior of this species. Marion directly participated in making these observations.

#### Mating behavior.

Marion Kotrba and W. N. Mathis observed the mating behavior of *Planinasus kotrbae* in the field (Ecuador. Orellana: Rio Tiputini Biodiversity Station (0°38.2'S, 76°08.9'W)) and in a rearing cage in the laboratory. In the field, we observed what we called “head butting” on repeated occasions, especially on leaves laying on the substrate. This refers to the specific behavior of males confronting each other head on and then lunging forward, making physical contact with the front of the head. Marion collected several specimens that she released into a rearing cage. Within minutes the specimens were mating and going through sequences of mating behavior. We observed 10 matings with the following durations: 1×2s, 2×5s, 1×10s, 4×15s, 2×18s. It cannot be decided whether all of these were successful in terms of sperm transfer. Two behavioral elements commonly observed within the context of mating were “kissing” and an “arc dance”. Between matings and also at other encounters the flies frequently made contact with the frontally extended proboscis. These “kisses” lasted up to about 4 seconds with sometimes only a brief second between. “Kissing” was observed a few times between specimens of the same gender. When “arc dancing” a male would shuttle back and forth laterally with spread wings while facing the female at a short distance of only a few centimeters. The lateral movements were usually in a short arc pattern of 30–45° but up to 180°. One observed mating sequence consisted of five matings, 24 kisses and four arc dances in total. Another male demonstrated no lateral movements after mating and kissing a female just once.

Having access to a rearing cage facilitated observations in the laboratory as well as temporarily holding specimens that were to be dissected. Keeping females to be dissected in the cage ensured their freshness, which makes dissecting easier, and the preparations that resulted were excellent. The tissue is pliable and extraneous material, such as tracheoles, can easily be removed.

#### Remarks.

This species is a member of the *shannoni* group and is distinguished from other congeners of that group by the black antenna in both sexes, the mostly shiny black face except for the whitish yellow, lateral margin immediately adjacent to the parafacial, the conically prominent face of the male, and the mostly yellowish femora (only the apices are dark colored). The presence of a basal projection on the surstylus also distinguishes this species from other members of the same species group.

Antennal bases of males more approximate, distance between slightly more than antennal length; face of males with a subshiny, black area extended obliquely medioventrally from ventral margin of antennal groove nearly to oral margin

### 5. Planinasus miradorus sp. n.

urn:lsid:zoobank.org:act:F9039118-26ED-40C3-841C-1ECE62557E02

http://species-id.net/wiki/Planinasus_miradorus

Figures 30 –34

#### Description of male.

Moderately small to medium-sized flies, body length 2.15–3.05 mm.

*Head*: Head ratio 0.53–0.56; frons brownish black to black, thinly invested with microtomentum, subshiny to shiny, except for densely microtomentose, anterolateral angles and undercut anterior margin; frons much wider than long, frontal ratio 0.35–0.38; interfrontal seta long, length subequal to that of lateral vertical seta. Scape and pedicel black; basal flagellomere with apical half black, basal half whitish yellow; pedicel with ventral projection long, about 1/2 length of basal flagellomere; basal flagellomere long, length about twice height at base; arista bearing 13–14 dorsal rays, 3–4 ventral rays; pedicel bearing 1 dorsoapical seta and 1 dorsal seta. Face generally yellow; very wide, facial ratio 0.96-1.00; dorsad of transverse carina wide, shield-like, medial portion in some specimens faintly blackish, very sparsely microtomentose with some silvery white microtomentum; ventral portion of face yellow, moderately densely microtomentose, silvery white, seriaceous; large facial setae variable, in about 2 transverse rows; dorsal row with porrect medial pair of setae, next laterally a large, dorsoclinate seta; ventral row with 1-2 ventroclinate seta. Clypeus yellow; palpus yellowish brown.

*Thorax*: Generally dark colored, black to blackish brown, anepimeron paler, brownish, with ventral margin yellowish; mesonotum thinly invested with microtomentum, appearing subshiny, with slightly metallic brown luster medially, becoming more steel blue laterally; postpronotum dark brown; area from postpronotum and extended through notopleuron mostly bare, shiny; anepisternum thinly invested with microtomentum, mostly appearing dull, grayish brown; other pleurites less densely invested. Wing conspicuously and uniformly infumate, brownish, base slightly paler, more hyaline. Forecoxa with base blackish brown, apical 2/3 yellow; midcoxa yellow; hindcoxa faintly brownish; trochanters, and base of femora yellow; forefemur with dorsum black or mostly black; forefemur with a preapical, whitish to yellowish partial annulus; basal 3 tarsomeres yellow; apical two brownish black; forefemur with posteroventral surface bearing 1 large seta at apical 1/3.

**Figures 30–33.**
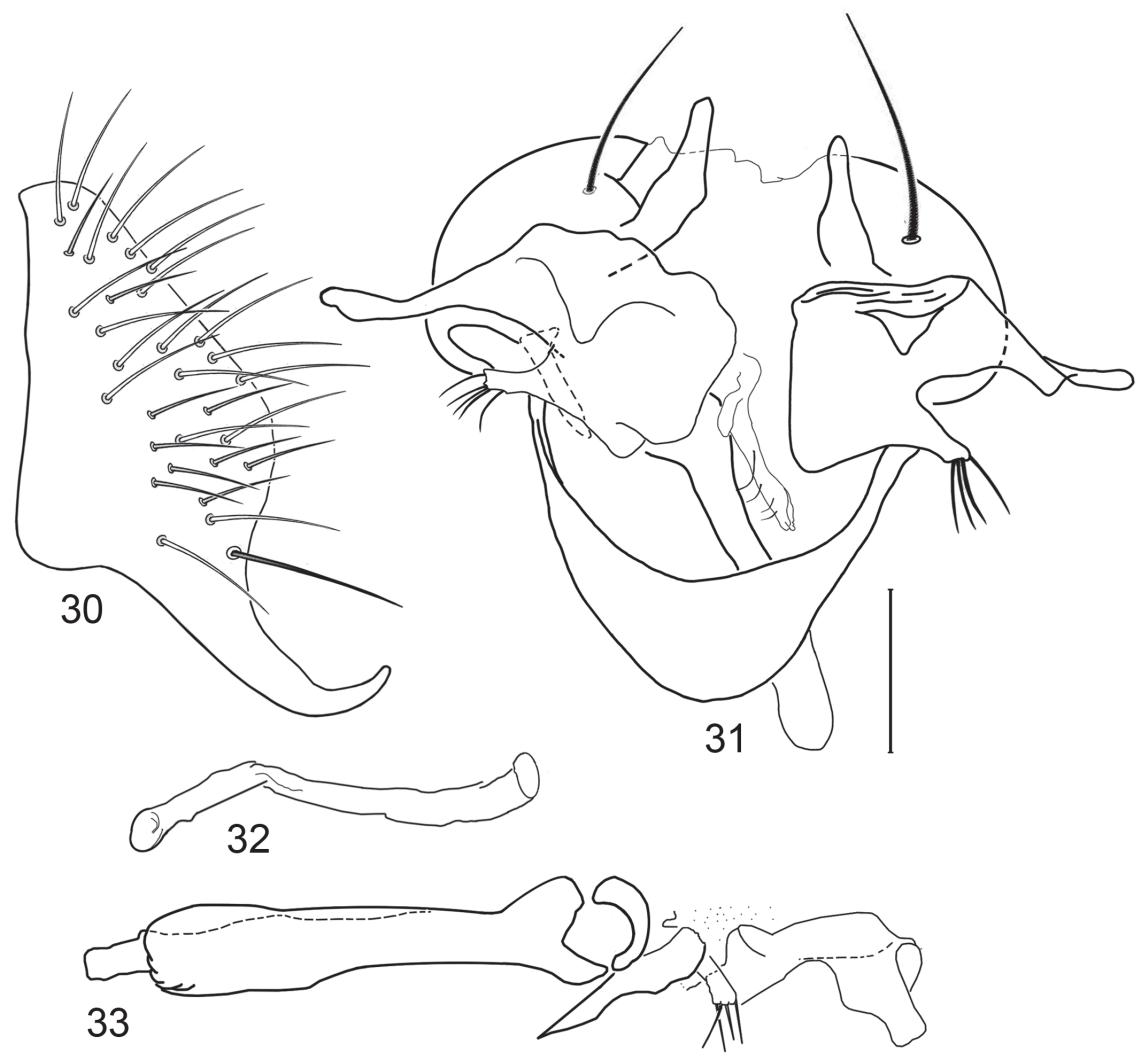
Illustrations of *Planinasus miradorus* sp. n. (male). **30** epandrium, surstylus, , lateral view **31** epandrium, hypandrium, and internal structures of male terminalia, ventral view **32** ejaculatory apodeme, lateral view **33** internal structures of male terminalia, lateral view. Scale bar = 0.1 mm.

*Abdomen*: Uniformly blackish brown to black, mostly shiny, very sparsely invested with microtomentum. Male abdomen: Tergites 1+2-6 well developed, lengths of tergites 3-6 subequal; tergite 7 narrow; sternites 3, 4, 5 generally as rectangular plates, slightly wider than long, with lateral margins shallowly arched; no sternites 6, 7, neither segment forming an annulus. Male terminalia (Figs 30-33): Epandrium in lateral view (Fig. 30) higher than wide, more or less triangular but with short dorsal surface truncate, anterior margin nearly straight, posterior margin nearly straight dorsally, ventral portion arched; surstylus almost as long as epandrium, extended from ventral margin of epandrium in nearly oblique alignment with it, in lateral view (Fig. 30) elongate, thinly developed, more or less “J” shaped, tapered, curved subapically, apex narrowly developed, bearing 1 large, basal setula; hypandrium in ventral view (Fig. 31) broadly U-shaped, robustly developed anteriorly, arms tapered, more slender than wide base, anterior margin broadly rounded;; postgonite in ventral view (Fig. 31) subquadrate, slightly wider than long with posterior lateral arms spread outwards; with lobe bearing setulae, in lateral view (Fig. 33) longer than wide, lobe digitiform, bearing 4 apical setulae; phallus in ventral view (Fig. 31) complex, partially sclerotized; phallapodeme elongate, slender, in lateral view (Fig. 33) parallel sided, tubular, nearly straight, rounded apically, in ventral view (Fig. 31) tapered, margins shallowly undulous, apex narrowly developed; ejaculatory apodeme , in lateral view (Fig. 32) almost as long as phallapodeme but only slightly longer than surstylus in lateral view, apex not expanded.

**Figure 34.**
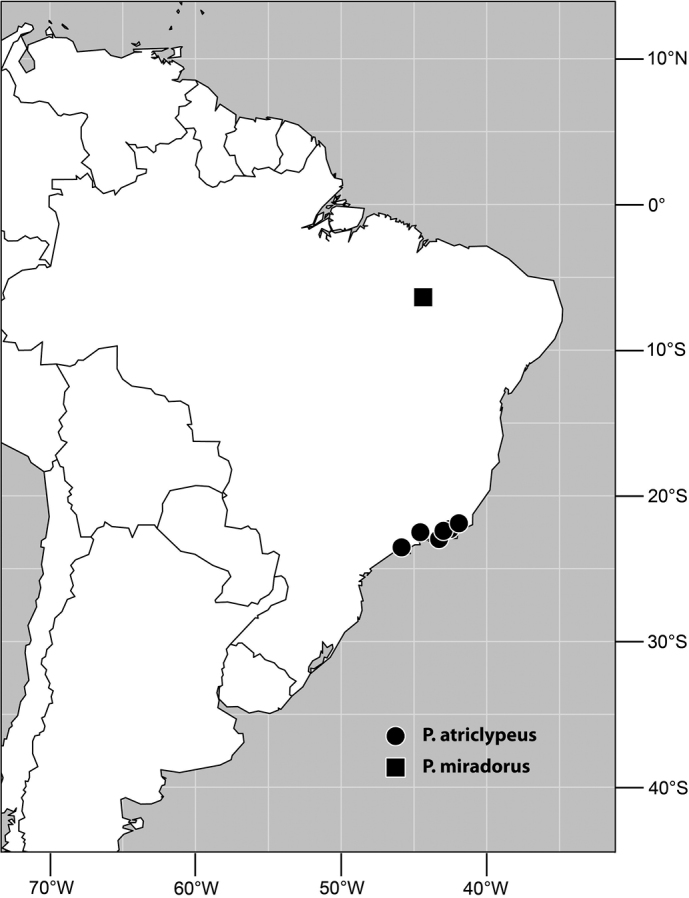
Distribution of *Planinasus miradorus* sp. n. (square) and *Planinasus atriclypeus* (dots).

#### Description of female.

Same as male except as follows: *Head*: Generally narrower; head ratio 0.68–0.71; frontal ratio 0.49–0.52; antenna black; face mostly bluish black, subshiny to shiny, laterally more microtomentose, grayish white; facial ratio 0.34-0.37.

*Thorax*: Forefemur lacking preapical annulus.

#### Type material.

The holotype male is labeled “Brasil (MA[RANHÃO]), Mirador Parque Est. Mirador Base da Geraldina/Armadilha Suspensa 23.ix.2006 [23 Sep 2006], F. Limeira de Oliveira, col./USNM ENT 00118277 [plastic bar code label]/**HOLOTYPE** ♂ *Planinasus miradorus* Mathis & Rung INPA [red].” The holotype is double mounted (glued to a paper point), is in good condition (left basal flagellomere missing), and is deposited in INPA. Six paratypes (3♂, 3♀; INPA, USNM) bear the same locality data as the holotype but with dates from 9 Aug-4 Dec 2006, 2007. One male paratype and all female paratypes were collected in a Malaise trap.

#### Type locality.

Brazil. Maranhão: Parque Estadual Mirador, Base da Geraldina (06°22.2'S, 44°21.8'W).

#### Distribution

(Fig. 34). *Neotropical*: Brazil (Maranhão).

#### Etymology.

The specific epithet, *miradorus*, is to recognize the name of the state park in the Brazilian state of Maranhão where the type locality is located and is a noun in apposition.

#### Remarks.

This species is very similar to *Planinasus shannoni* and especially to *Planinasus venezuelensis* but can be distinguished from these and other congeners of the *shannoni* group by the distinctively colored male basal flagellomere, which is markedly two-toned with the base whitish yellow and the apical half black. The surstylus is very similar in shape to that of *Planinasus venezuelensis*, but other characters of the male terminalia, such as the relatively well-developed ejaculatory apodeme, the outspread arms of the postgonite in ventral view, and the general shape of the postgonite in lateral view distinguish this species from *Planinasus venezuelensis*. Furthermore, colorational characters of the basal flagellomere are very distinctive and constant in the sampling available to us, and based on this evidence, we are currently of the opinion that this is a separate species. Species of the *shannoni* group, this species and *Planinasus venezuelensis* in particular, would be excellent candidates for molecular analysis to test the strength of the morphological characters.

### 6. Planinasus shannoni

(Malloch)

http://species-id.net/wiki/Planinasus_shannoni

Figures 5 35–38

Schizochaeta shannoni Malloch 1934: 53.
Planinasus shannoni . Hennig 1969: 614 [generic combination]. Mathis and Rung 2011: 363 [world catalog].

#### Description of male.

Moderately small flies, body length 2.35–2.55 mm.

*Head*: Frons mostly bare of microtomentum, shiny, except for densely microtomentose, velvety-appearing, anterolateral angles, blackish brown; frons much wider than long, frontal ratio averaging 0.42; interfrontal seta long, length subequal to that of lateral vertical seta. Scape and pedicel blackish brown except for whitish yellow, ventral projection of pedicel; pedicel with ventral projection long, about 1/2 length of basal flagellomere; basal flagellomere long, slightly more than twice basal width, yellowish; arista bearing 13–14 dorsal rays, 3–4 ventral rays. Face very wide (at least of male), facial ratio 0.67; dorsal 2/3 (above transverse carina) wide, shield-like, medial portion (between antennal bases) moderately densely microtomentose, mostly whitish but with blackish brown background coloration also appearing, lateral portion (just below antennae) bare, shiny, especially ventrally, and with ventral margin of antennal grooves quite evident; ventral 1/3 of face (below transverse facial carina) mostly densely microtomentose, somewhat shiny, whitish to whitish yellow, with blackish, somewhat bare, narrowly triangular area extended obliquely medioventrally from ventral margin of antennal groove; large facial setae 3, not arranged in transverse rows, becoming smaller ventrally; largest facial seta inserted dorsolaterally, dorsoclinate and convergent; 2nd largest seta inserted medioventrally from largest seta, porrect and parallel; 3rd seta inserted ventrolaterally and with porrect to slightly ventroclinate orientation. Clypeus and palpus blackish brown. Gena concolorous with ventral 1/3 of face.

*Thorax*: Generally dark colored, black to blackish brown, anepimeron paler, brownish, with ventral margin yellowish; mesonotum thinly invested with microtomentum, appearing subshiny, with slightly metallic brown luster medially, becoming more steel blue laterally; postpronotum dark brown; area from postpronotum and extended through notopleuron mostly bare, shiny; anepisternum thinly invested with microtomentum, mostly appearing dull, grayish brown; other pleurites less densely invested. Wing conspicuously and uniformly infumate, brownish, base slightly paler, more hyaline. Coxae yellowish; forefemur mostly blackish, only basal 1/8 yellowish, also with a preapical, yellowish annulus; middle and hind femora mostly yellowish, apical 1/4–1/3 dark brown; tibiae blackish brown; tarsi, except apical 1–2 dark brown tarsomeres, yellowish. Forefemur with posteroventral surface bearing 1 large seta at apical 1/3.

**Figures 35–38.**
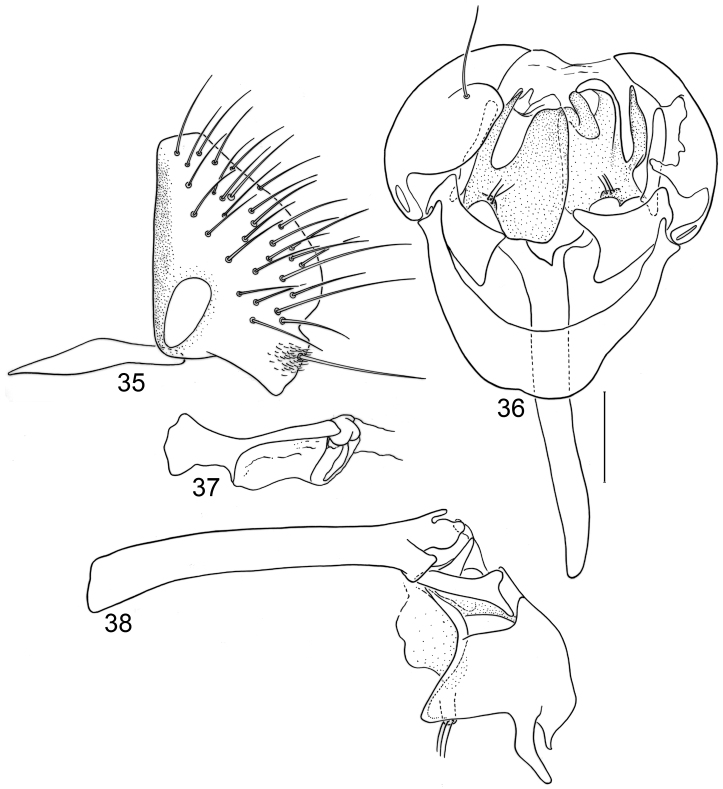
Illustrations of *Planinasus shannoni* (Malloch) (male). **35** epandrium, surstylus, hypandrium, lateral view **36** structures of internal male terminalia, ventral view **37** ejaculatory apodeme, lateral view **38** internal structures of male terminalia, lateral view. Scale bar = 0.1 mm.

*Abdomen*: Uniformly blackish brown to black, mostly shiny, very sparsely invested with microtomentum. Male abdomen: Tergites 1+2-6 well developed, lengths of tergites 3-6 subequal; tergite 7 narrow; sternites 3, 4, 5 generally as rectangular plates, slightly wider than long, lateral margins shallowly arched; no sternites 6, 7, neither segment forming an annulus. Male terminalia (Figs 35-38): Epandrium in lateral view (Fig. 35) higher than wide, more or less triangular but with dorsal surface truncate, short, anterior margin nearly straight, posterior margin nearly straight dorsally, ventral portion arched; surstylus length less than 1/3 length of epandrium, extended from ventroposterior margin of epandrium in nearly oblique alignment with epandrium, in lateral view (Fig. 35) short, widely truncate apically, bearing 1 large, basal setula; hypandrium in ventral view (Fig. 36) broadly U-shaped, robustly developed anteriorly, arms tapered, more slender than wide base, anterior margin rounded; pregonite in ventral view (Fig. 36) approximately triangular to subrectangular, with anterior and lateral margins receded and slightly round corners; postgonite in ventral view (Fig. 36) convoluted, bearing a ventral digitiform lobe with approximately 3 setulae; phallus in ventral view (Fig. 38) complex, partially sclerotized; phallapodeme elongate, in lateral view (Fig. 38) parallel sided, tubular, nearly straight, truncate apically, in ventral view (Fig. 36) slightly tapered, apex narrowly developed; ejaculatory apodeme in lateral view (Fig. 37) almost as long as ½ length of phallapodeme, apex only slightly expanded.

**Description of female.** Same as male except as follows: *Head*: Frontal ratio 0.55; face and antenna entirely blackish brown; face not as wide, facial ratio 0.32; face projected forward on ventral 1/2, bulbous, evenly arched transversely, shallowly arched vertically, mostly flat.

*Thorax*: Forefemur lacking preapical annulus.

**Type material.** The holotype male is labeled “IquitosPeru MarApr1931 RCShannon/Type No. 49549 U.S.N.M. [red, number handwritten]/Schizochaeta shannoni Type det. JRMALLOCH [species name and “Type” handwritten, label has a black sub-border].” The holotype is double mounted (glued to a paper point), is in fair condition (the middle and hind right legs are missing), and is deposited in the USNM (49549). The allotype female and four additional paratypes, all males, bear the same locality, date, and collector data as the holotype. In the original description, Malloch stated that a paratype would be deposited in the British Museum (Natural History).

**Type locality.** Peru. Loreto: Iquitos (03°45'S, 73°16.3'W).

**Other specimens examined.** *ECUADOR. Orellana*: Rio Tiputini Biodiversity Station (0°38.2'S, 76°8.9'W), 12–26 Aug 1999, A. Baptista, M. Kotrba, W. N. Mathis (2♂; USNM, ZSMC).

**Distribution** (Fig. 5). *Neotropical*: Ecuador (Orellana), Peru (Loreto).

**Etymology.** Malloch named this species after his friend and fellow dipterist, Raymond Corbett Shannon, who was the collector of the type series. R. C. Shannon conducted much field work in the Neotropical Region and most of his collections were donated to the USNM.

**Remarks.** This species is very similar to *Planinasus venezuelensis* but is distinguished from it by the mostly yellow mid- and hindfemora, which have at most the apical one-fourth to one-third dark, and the shape of the surstylus (Fig. 35) short and widely truncate apically. The surstylus of *Planinasus venezuelensis* is long, J-shaped. From other congeners, this species is distinguished by the following characters of males: the pale-colored basal flagellomere and apex of the ventral projection of the pedicel, the whitish microtomentum on the mid-dorsal surface of the face, the relatively approximate antennal bases, and the narrowly triangular areas that extend medioventrally from the ventral margin of the antennal groove. The shape of structures of the male terminalia also clearly distinguishes this species (see Figs 35–38).

### 7. Planinasus tobagoensis sp. n.

urn:lsid:zoobank.org:act:03C8E4C6-84A2-4868-9651-981E4FF3438E

http://species-id.net/wiki/Planinasus_tobagoensis

Figures 39 –43

#### Description of male.

Moderately small to medium-sized flies, body length 2.10–3.10 mm.

*Head*: Head ratio 0.53–0.56; frons mostly bare of microtomentum, shiny, except for densely microtomentose, velvet-like, anterolateral corner, blackish brown; frons much wider than long, frontal ratio 0.35–0.37; interfrontal seta long, length subequal to that of lateral vertical seta. Antenna blackish brown; pedicel with ventral projection moderately long, about 1/2 length of basal flagellomere; basal flagellomere long, length slightly more than twice basal width; arista bearing 13–14 dorsal rays, 3–4 ventral rays. Face very wide, facial ratio 0.81–0.83; face dorsad of transverse carina, including antennal grooves, mostly black to deep bluish black, face ventrad of transverse carina with broad mediovertical, black vitta, ventrolateral portion of face becoming progressively lighter in color, yellowish laterally; parafacial yellow; large facial setae 4–5 on each side, 2 medial, porrect to shallowly ventroclinate setae in vertical alignment, larger seta ventrad; next seta toward lateral margin dorsoclinate to inclinate, aligned with dorsal medial seta; next 2 lateral setae, more or less aligned vertically, both shallowly ventroclinate. Clypeus and palpus blackish brown. Gena concolorous with lateral margin of face.

*Thorax*: Mesonotum generally brownish black to deep bluish black, very thinly microtomentose, subshiny to mostly shiny; postpronotum dark brown with some yellowish coloration around margin; area from postpronotum and through notopleuron mostly bare, finely microtomentose, dull to subshiny; pleural areas finely microtomentose, dull, blackish brown; anepisternum mostly bare except for 2–3 setulae along posterior margin; katepisternum generally setulose, bearing 2 setae toward dorsal margin. Wing without pattern, generally infumate, slightly more so anteriorly, base hyaline. Coxae and trochanters whitish yellow to yellow; femora and tibiae uniformly brownish black; forefemur with preapical, pale annulus lacking or weakly indicated; tarsi mostly yellowish, apical 1-2 tarsomeres becoming darker; forefemur bearing 1 seta at apical 1/3 along posteroventral surface.

**Figures 39–42.**
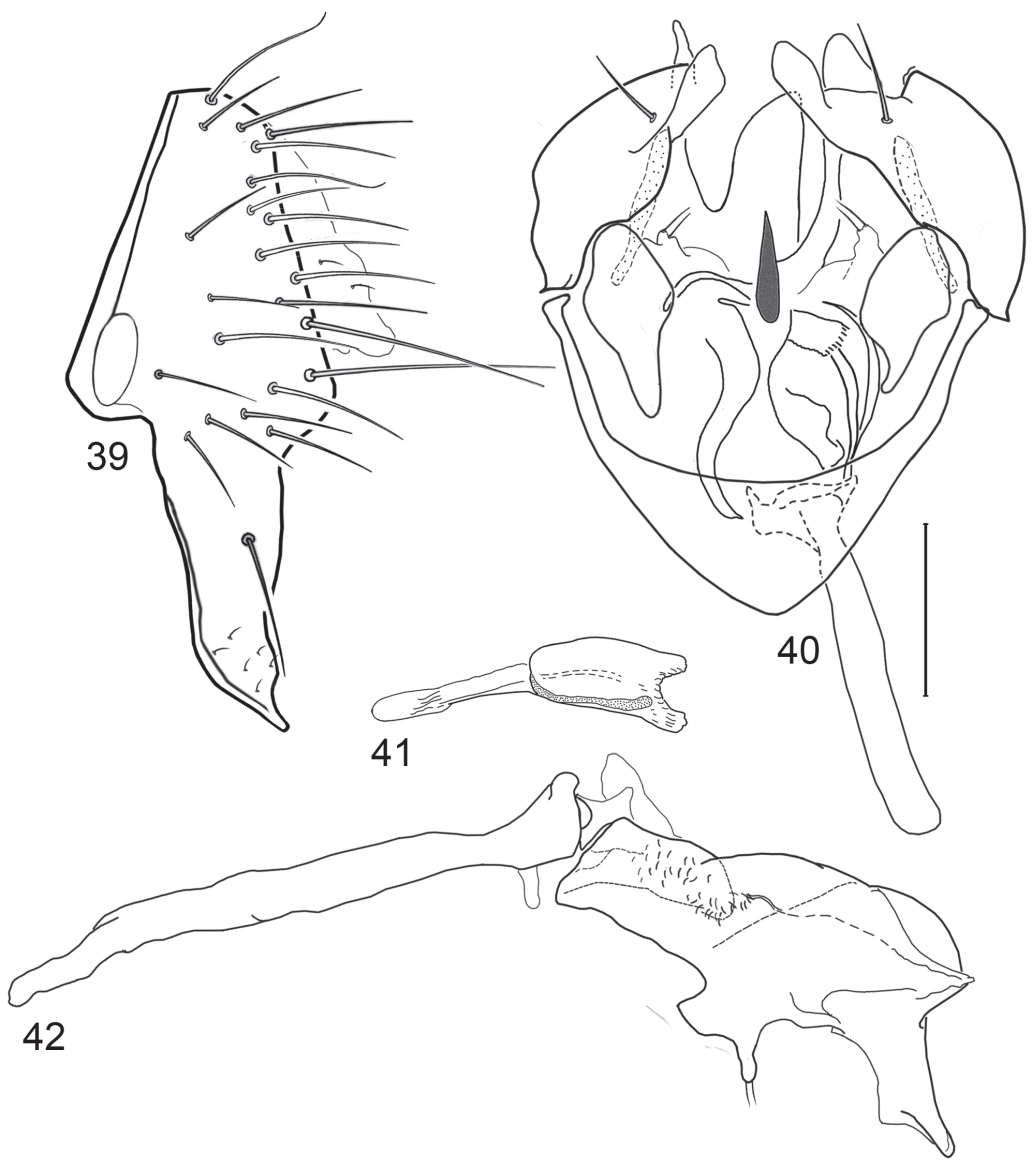
Illustrations of *Planinasus tobagoensis* sp. n. (male). **39** epandrium, surstylus, lateral view **40** structures of internal male terminalia, ventral view **41** ejaculatory apodeme, lateral view **42** internal structures of male terminalia, lateral view. Scale bar = 0.1 mm.

*Abdomen*: Uniformly blackish brown to black, mostly shiny, very sparsely invested with microtomentum. Male abdomen: Tergites 1+2-6 well developed, lengths of tergites 3-6 subequal; tergite 7 narrow; sternites 3, 4, 5 generally as rectangular plates, slightly wider than long, lateral margins shallowly arched; no sternites 6, 7, neither segment forming an annulus. Male terminalia (Figs 39-42): Epandrium in lateral view (Fig. 39) trapezoidal, higher than wide, anterior and posterior margins nearly straight, dorsal margin short, length slightly less than surstylar width at base, slightly arched; surstylus as long as epandrium, in lateral view (Fig. 39) extended from ventral margin of epandrium in nearly vertical alignment with it, tapered, anterior margin arched, especially subapically, posterior margin shallow sinuous, apex acutely pointed, bearing 1 large, medial setula; hypandrium in ventral view (Fig. 40) more or less V-shaped, arms in ventral view (Fig. 40) thin at attachment with epandrium, anterior margin thickened anteromedially, broadly pointed, forming a right angle; pregonite irregularly shaped, roughly diamond-shaped (Fig. 40); postgonite in ventral view (Fig. 40) convoluted, bearing lobe with setae, in lateral view (Fig. 42) lobe bearing 2 apical setulae; phallus in ventral view (Fig. 40) complex, mostly membranous, in lateral view (Fig. 42) thumb-like, bearing tiny setulae; phallapodeme in lateral and ventral views (Figs 40, 42) elongate, tubular, nearly straight, bluntly rounded apically; ejaculatory apodeme much reduced, length subequal to ½ length of phallapodeme , in lateral view (Fig. 41) with basal half enlarged, basal margin deeply incised, apical half narrow, rod-like, lacking fan-like process.

#### Description of female.

Same as male except as follows: *Head*: Head ratio 0.70–0.74; frontal ratio 0.43–0.45; face and antenna mostly blackish brown, lateral margin of face (immediately adjacent to parafacial) whitish yellow; face not as wide, facial ratio 0.43–0.45; face projected forward on ventral 1/2, bulbous, evenly arched transversely, shallowly arched vertically, mostly flat.

**Figure 43.**
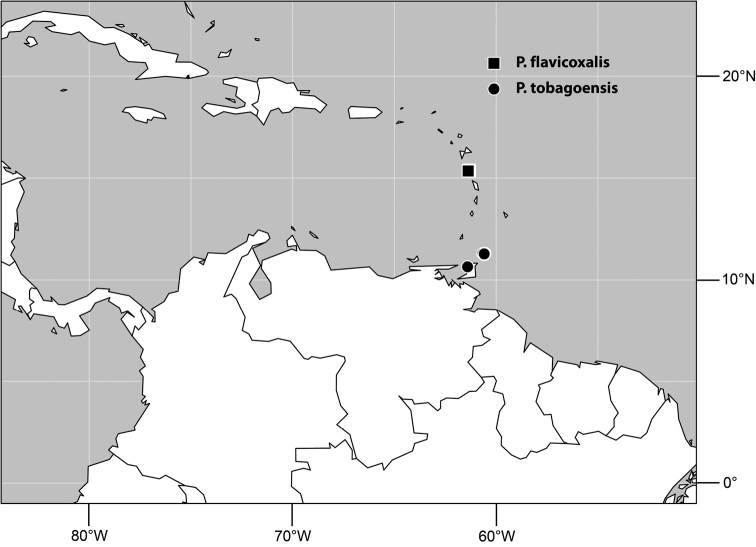
Distribution of *Planinasus tobagoensis* sp. n. (dots) and *Planinasus flavicoxalis* sp. n. (square).

#### Type material. 

The holotype male is labeled “**TOBAGO.** St. John: Parlatuvier (11°18'N, 60°39'W), 14 Jun 1993, W.N. Mathis/USNM ENT 00118281 [plastic bar code label]/**HOLOTYPE** ♂ *Planinasus tobagoensis* Mathis & Rung USNM [red]. The holotype is double mounted (minuten pin in a plastic block), is in excellent condition, and is deposited in the USNM. Seven paratypes (3♂, 4♀; USNM) bear the same locality data as the holotype but with dates from 20 Apr–14 Jun 1993, 1994. Other paratypes are as follows: TRINIDAD and TOBAGO. Tobago. *St. John*: Charlotteville (2 km S; 11°19'N, 60°33'W), 10 Jun 1993, W. N. Mathis (1♀; USNM). *St. Paul*: Argyle Falls (11°15'N, 60°35'W), 21 Apr 1994, W. N. Mathis (1♂; USNM); Roxborough (6 km NNW; 11°16'N, 60°35.4'W), 20 Apr 1994, W. N. Mathis (1♂; USNM); Roxborough (6.5 km N; 11°17'N, 60°35'W), 14 Jun 1993, W. N. Mathis (1♂; USNM). *TRINIDAD. St. George*: Mount St. Benedict (10°39'N, 61°24'W), 18–21 Jun 1993, W. N. Mathis (2♂, 1♀; USNM).

#### Type locality.

Trinidad and Tobago. Tobago. St. John: Parlatuvier (11°17.9'N, 60°39'W).

#### Other specimens examined.

*ECUADOR. Orellana*: Rio Tiputini Biodiversity Station (0°38.2'S, 76°8.9'W), 12–26 Aug 1999, A. Baptista, M. Kotrba, W. N. Mathis (1♀; ZSMC).

*GUYANA*. Kumu River and Falls (25 km SE Lethem in Kanuku Mountains; 3°15.9'N, 59°43.6'W), 28–30 Apr 1995, W. N. Mathis (1♀; USNM).

#### Distribution

(Fig. 43). *Neotropical*: Ecuador (Orellana), Guyana, Trinidad and Tobago.

#### Etymology.

The specific epithet, *tobagoensis*, is to recognize the island where the type locality is located.

#### Remarks. 

This species is known from northern South America, including Trinidad and Tobago. It keys out near *Planinasus kotrbae*. Besides the characters given in the key, it can be easily distinguished from that species by the absence of a basal process on the surstylus and a roughly diamond-shaped pregonite (roughly triangular in *Planinasus kotrbae*).

### 8. Planinasus venezuelensis

Hennig

http://species-id.net/wiki/Planinasus_venezuelensis

Figures 44 –47

Planinasus venezuelensis Hennig 1969: 615. Mathis and Rung 2011: 363 [world catalog].

#### Description of male.

Moderately small to medium-sized flies, body length 2.35–3.10 mm.

*Head*: Frons mostly bare of microtomentum, shiny, except for densely microtomentose, velvety-appearing, anterolateral angles, blackish brown; frons much wider than long, frontal ratio 0.40–0.45; interfrontal seta long, length subequal to that of lateral vertical seta. Scape and pedicel blackish brown except for whitish yellow, ventral projection of pedicel; pedicel with ventral projection long, about 1/2 length of basal flagellomere; basal flagellomere long, slightly more than twice basal width, yellowish; arista bearing 13–14 dorsal rays, 3–4 ventral rays. Face variable, wide to very wide (at least of male), facial ratio 0.85–1.25; dorsal 2/3 (above transverse carina) wide, shield-like, medial portion (between antennal bases) moderately densely microtomentose, mostly whitish but with blackish brown background coloration also appearing, lateral portion (just below antennae) bare, shiny, especially ventrally, and with ventral margin of antennal grooves quite evident; ventral 1/3 of face (below transverse facial carina) mostly densely microtomentose, somewhat shiny, whitish to whitish yellow, with blackish, somewhat bare, narrowly triangular area extended obliquely medioventrally from ventral margin of antennal groove; large facial setae 3, not arranged in transverse rows, becoming smaller ventrally; largest facial seta inserted dorsolaterally, dorsoclinate and convergent; 2nd largest seta inserted medioventrally from largest seta, porrect and parallel; 3rd seta inserted ventrolaterally and with porrect to slightly ventroclinate. Clypeus and palpus blackish brown. Gena concolorous with ventral 1/3 of face.

*Thorax*: Generally dark colored, black to blackish brown, anepimeron paler, brownish, with ventral margin yellowish; mesonotum thinly invested with microtomentum, appearing subshiny, with slightly metallic brown luster medially, becoming more steel blue laterally; postpronotum dark brown; area from postpronotum and extended through notopleuron mostly bare, shiny; anepisternum thinly invested with microtomentum, mostly appearing dull, grayish brown; other pleurites less densely invested. Wing conspicuously and uniformly infumate, brownish, base slightly paler, more hyaline. Coxae yellowish; femora mostly blackish, basal areas yellowish and forefemur with a preapical yellowish annulus; tibiae blackish brown; tarsi, except apical 1–2 dark brown tarsomeres, yellowish. Forefemur with posteroventral surface bearing 1 large seta at apical 1/3.

**Figures 44–46.**
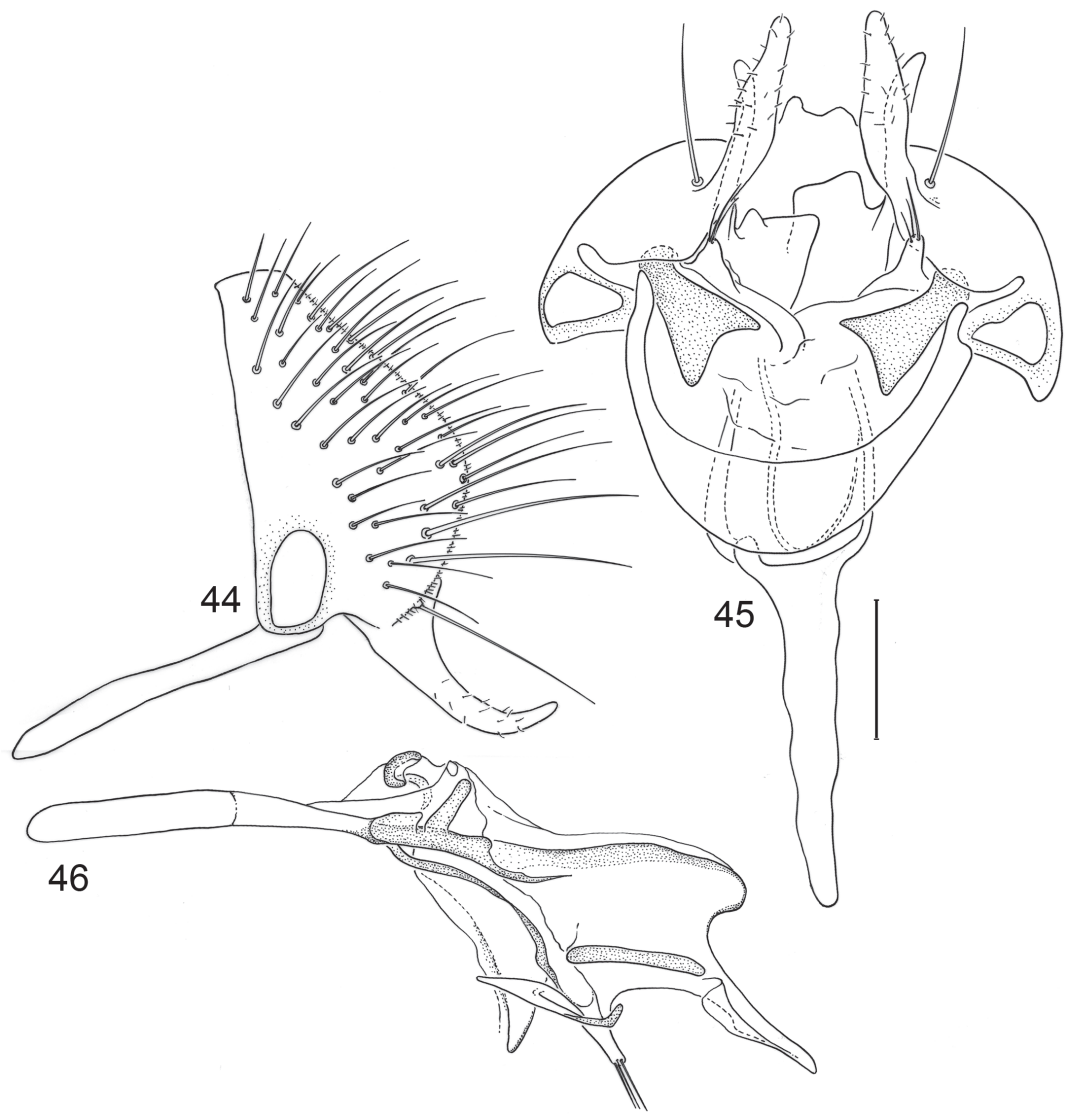
Illustrations of *Planinasus venezuelensis* Hennig (male). **44** epandrium, surstylus, hypandrium, lateral view **45** structures of internal male terminalia, ventral view **46** internal structures of male terminalia, lateral view. Scale bar = 0.1 mm.

*Abdomen*: Uniformly blackish brown to black, mostly shiny, very sparsely invested with microtomentum. Male abdomen: Tergites 1+2–6 well developed, lengths of tergites 3–6 subequal; tergite 7 narrow; sternites 3, 4, 5 generally as rectangular plates, slightly wider than long, lateral margins shallowly arched; no sternites 6, 7, neither segment forming an annulus. Male terminalia (Figs 44–46): Epandrium in lateral view (Fig. 44) higher than wide, more or less triangular but with short dorsal surface truncate, anterior margin nearly straight, posterior margin nearly straight dorsally, ventral portion arched; surstylus almost as long as epandrium, extended from ventral margin of epandrium in nearly oblique alignment with it, in lateral view (Fig. 44) elongate, thinly developed, more or less “J” shaped, tapered, curved subapically, apex narrowly developed, bearing 1 large, basal setula; hypandrium in ventral view (Fig. 45) broadly U-shaped, robustly developed anteriorly, arms tapered, more slender than wide base, anterior margin broadly rounded; pregonite in ventral view (Fig. 45) approximately triangular, with anterior margin receded and slightly round corners; postgonite in ventral view (Fig. 45) elongate, longer than wide, with lobe bearing a few setulae, in lateral view (Fig. 46) longer than wide, lobe digitiform, bearing 3 apical setulae; phallus in ventral view (Fig. 45) complex, partially sclerotized; phallapodeme elongate, slender, in lateral view (Fig. 46) parallel sided, tubular, nearly straight, rounded apically, in ventral view (Fig. 45) tapered, margins shallowly undulous, apex narrowly developed; ejaculatory apodeme greatly reduced.

#### Description of female.

Same as male except as follows: *Head*: Frontal ratio averaging 0.57; face and antenna entirely blackish brown; face not as wide, facial ratio averaging 0.44; face projected forward on ventral 1/2, bulbous, evenly arched transversely, shallowly arched vertically, mostly flat.

**Figure 47.**
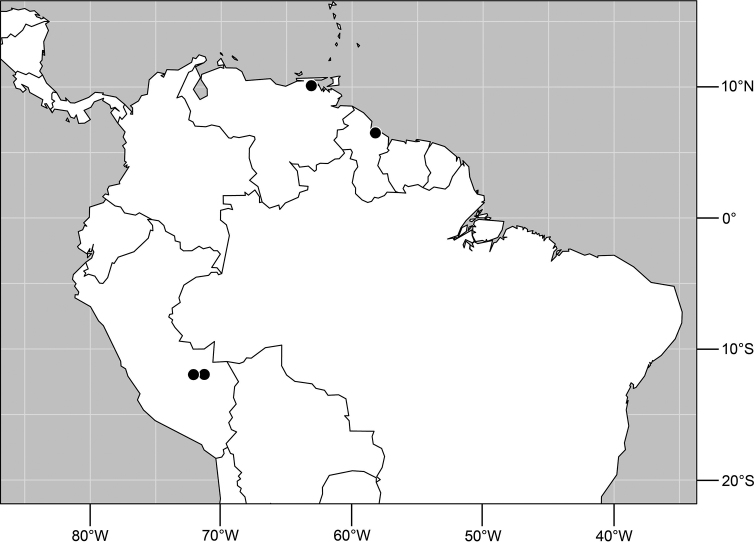
Distribution of *Planinasus venezuelensis* Hennig.

#### Type material.

The holotype male is labeled “Caripito [10°06.6'N, 63°06.8'W], Venezuela 26-III 1942 [day and month handwritten, 26 Mar 1942]/HOLOTYPUS [red]/Planinasus venezuelensis Hennig [handwritten].” The holotype is directly pinned, is in good condition, and is deposited in the American Museum of Natural History.

#### Type locality.

Venezuela. Monagas: Caripito (10°06.6'N, 63°06.8'W).

#### Other specimens examined.

*GUYANA*. Conservation of Ecological Interactions and Biotic Associations (CEIBA; ca. 40 km S Georgetown; 06°29.9'N, 58°13.1'W), 13 Apr-28 Aug 1994, 1997, W. N. Mathis (5♂, 2♀; USNM).

*PERU. Madre de Dios*: Río Manu, Pakitza (11°56.6'S, 71°16.9'W; 250 m), 9–23 Sep 1988, A. Freidberg, W. N. Mathis (28♂, 25♀; USNM); Rio Manu, Cocha Salvador (11°57'S, 72°07'W; 240 m), 14 Sep 1988, W. N. Mathis (1♀; USNM).

#### Distribution

(Fig. 47). *Neotropical*: Guyana, Peru (Madre de Dios), and Venezuela (Monagas).

#### Natural history.

Adults of this species were fairly common on vegetation in the understory of gallery forests along the Rio Manu. The flies occurred on prominent, frequently sun-lit, broad leaves, especially in stream bottoms, where males displayed and courted females and drove off other males.

#### Remarks.

This species is very similar to *Planinasus shannoni* and *Planinasus miradorus* but can be distinguished from these and other congeners of the *shannoni* group by the following characters of males: the pale-colored, unicolorous basal flagellomere and apex of the ventral projection of the pedicel, the whitish microtomentum on the mid-dorsal surface of the face, the relatively approximate antennal bases, and the narrowly triangular areas that extend medioventrally from the ventral margin of the antennal groove. Males and females are distinguished by the mostly black middle and hind femora (only the basal one-fourth to one-third yellowish in males, and for females only the basal one-eighth is yellowish). See “Remarks” under *Planinasus venezuelensis* for further discussion of similarities and differences.

### 9. Planinasus xanthops sp. n.

urn:lsid:zoobank.org:act:6C187AF2-BC7F-4BDC-BF53-E699ED5FE7A9

http://species-id.net/wiki/Planinasus_xanthops

Figures 48 –52

#### Description of male.

Moderately small to medium-sized flies, body length 2.10–3.05 mm.

*Head*: Head ratio 0.56–0.58; frons mostly bare of microtomentum, subshiny to shiny, except for densely microtomentose, anterolateral angles and undercut anterior margin, blackish brown; frons much wider than long, frontal ratio 0.36–0.39; interfrontal seta long, length subequal to that of lateral vertical seta. Antenna yellow; pedicel with ventral projection long, about 1/2 length of basal flagellomere; basal flagellomere long, about twice height at base; arista bearing 13–14 dorsal rays, 3–4 ventral rays; pedicel bearing 1 dorsoapical seta and 1 dorsal seta. Face generally yellow; very wide, facial ratio 0.69–0.73; dorsad of transverse carina wide, shield-like, medial portion in some specimens faintly brownish yellow, very sparsely microtomentose with some silvery white microtomentum; ventral portion of face yellow, sparsely microtomentose, somewhat seriaceous; large facial setae in 2–3 transverse rows; dorsal row with porrect medial pair of setae, next laterally a large, dorsoclinate seta; ventral row with a ventroclinate seta immediately ventrad of dorsoclinate seta, then with 2 smaller, ventroclinate setae. Clypeus yellow to brownish yellow; palpus yellowish brown. Gena concolorous with ventral 1/3 of face.

*Thorax*: Generally dark colored, black to blackish brown, anepimeron paler, brownish, with ventral margin yellowish; mesonotum thinly invested with microtomentum, appearing subshiny, with slightly metallic brown luster medially, becoming more steel blue laterally; postpronotum dark brown; area from postpronotum and extended through notopleuron mostly bare, shiny; anepisternum thinly invested with microtomentum, mostly appearing dull, grayish brown; other pleurites less densely invested. Wing conspicuously and uniformly infumate, brownish, base slightly paler, more hyaline. Coxae, trochanters, and base of femora yellow; remainder of femora and tibiae brownish black; forefemur with a preapical, whitish to yellowish partial annulus; basitarsomeres yellow; remaining tarsomeres becoming darker with apical 1–2 dark brown; forefemur with posteroventral surface bearing 1 large seta at apical 1/3.

**Figures 48–51.**
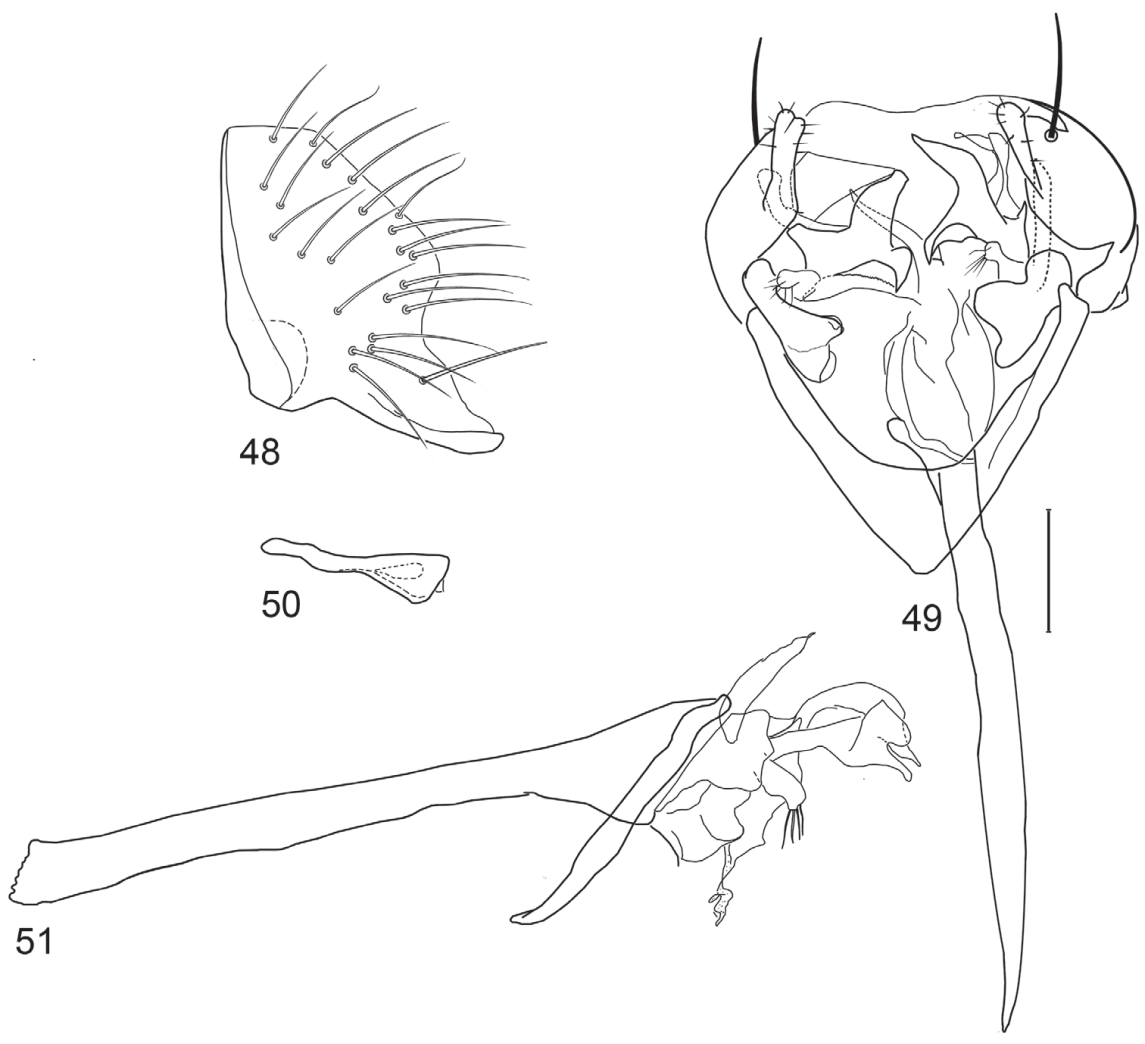
Illustrations of *Planinasus xanthops* sp. n. (male). **48** epandrium, surstylus, lateral view **49** structures of internal male terminalia, ventral view **50** ejaculatory apodeme, lateral view **51** phallus, phallapodeme, pre- and postgonite, lateral view. Scale bar = 0.1 mm.

*Abdomen*: Uniformly blackish brown to black, mostly shiny, very sparsely invested with microtomentum. Male abdomen: Tergites 1+2–6 well developed, lengths of tergites 3–6 subequal; tergite 7 narrow; sternites 3, 4, 5 generally as rectangular plates, slightly wider than long, lateral margins shallowly arched; sternite 5 with posterior margin of sclerotized portion shallowly arched posteriorly, bearing medially a patch of 10–12 setulae; no sternites 6, 7, neither segment forming an annulus. Male terminalia (Figs 48–51): Epandrium in lateral view (Fig. 48) higher than wide, anterior margin nearly straight, posterior margin irregularly arched, dorsal portion nearly straight, thereafter ventrally recurved to surstylar base, dorsal margin short, length about equal to surstylar width at base, truncate; surstylus length a little longer than half length of epandrium, extended from ventroposterior margin of epandrium in nearly oblique alignment with it, in lateral view (Fig. 48) triangular, tapered to narrow apex, anterior margin nearly straight, posterior margin shallowly sinuous, bearing 1 large, basal seta; hypandrium in ventral view (Fig. 49) more or less V-shaped, arms at attachment with epandrium thin, becoming gradually and slightly thicker toward anterior margin, anterior margin angulate, pointed; pregonite in ventral view (Fig. 49) roughly triangular, with anterior and lateral margins receded and slightly round corners; postgonite in ventral view (Fig. 49) convoluted, with lobe bearing setulae, especially at apex, in lateral view (Fig. 51) irregularly, bearing finger-like lobe with 4 apical setulae; phallus in ventral view (Fig. 49) complex, partially sclerotized, in lateral view (Fig. 51) difficult to discern; phallapodeme in lateral and ventral views (Figs 49, 51) elongate, tubular, nearly straight, in ventral view very gradually tapered to acute point, in lateral view (Fig. 51) parallel sided, truncate apically; ejaculatory apodeme short, length subequal to 1/4 length of phallapodeme, thickened basally, tapered to thin, sinuous apical half without expansion.

**Figure 52.**
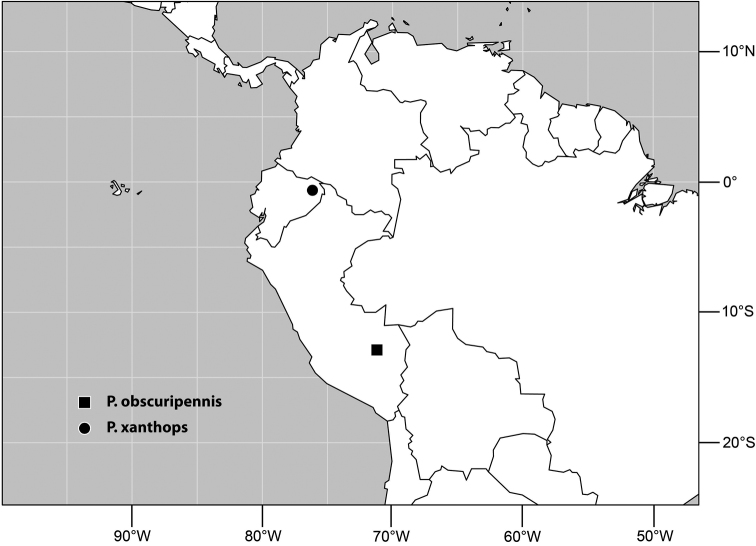
Distribution of *Planinasus xanthops* sp. n. (dot) and *Planinasus obscuripennis* sp. n. (square).

#### Description of female.

Same as male except as follows: *Head*: Head generally narrower; head ratio 0.69; frontal ratio 0.49; antenna black; dorsad of facial carina blackish brown; ventrad of facial carina yellowish; facial ratio 0.38.

*Thorax*: Forefemur lacking preapical annulus.

#### Type material.

The holotype male is labeled “**ECUADOR.** Prt.Or[e]ll[a]na: RioTiputini (0°38.2'S, 76°8.9'W)[,]12–26Aug1999,W.N.Mathis, A. Baptista, M. Kotrba/USNM ENT 00118278 [plastic bar code label]/**HOLOTYPE** ♂ *Planinasus xanthops* Mathis & Rung USNM [red].” The holotype is double mounted (minuten pin in a plastic block), is in excellent condition, and is deposited in the USNM. Four paratypes (3♂, 1♀; USNM) bear the same label data as the holotype.

#### Type locality.

Ecuador. Orellana: Rio Tiputini Biodiversity Station (0°38.2'S, 76°8.9'W).

#### Distribution 

(Fig. 52). *Neotropical*: Ecuador (Orellana).

#### Etymology.

The specific epithet, *xanthops*, is of Greek derivation, meaning yellow face, and refers to yellow face and antennae of males.

##### The *nigritarsus* group

**Included species.** *Planinasus argentifacies* sp. n.,* P. insulanus* sp. n.,* P. nigritarsus* sp. n.

**Diagnosis.** This species group is distinguished by the following combination of characters: *Head*: Interfrontal seta long, subequal to length of lateral vertical seta. Antenna yellow to yellowish orange with some blackish coloration dorsally; pedicel with short ventral projection; basal flagellomere long, length nearly twice height. Large facial setae arranged in a single transverse row of about 8 setae; face of females somewhat bulbous, evenly transversely arched, mostly black dorsally, densely silvery white, microtomentose ventrally. *Thorax*: Anepisternum with 3–4 small setae along posterior margin. Wing moderately to strongly infumate. Forefemur of male lacking a pale, irregular, subapical annulus, bearing 1 large, posteroventral seta at apical 1/3. *Abdomen*: Surstylus generally bearing a basal process and generally in nearly oblique alignment with epandrium; postgonite with robustly developed lobe bearing numerous setulae apically; phallus mostly sclerotized, convoluted; ejaculatory apodeme generally well-developed, at least as long as phallapodeme, with expanded apex.

**Discussion.** Species of this species group share, with species of the *ambiguus* group, and only with them, a postgonite with a robustly developed lobe that bears numerous setulae. In other species of *Planinasus*, the lobe of the postgonite, which is generally less well developed, bears fewer than four apical setulae, and fewer than six setulae overall. The *nigritarsus* and *ambiguus* groups also share an ejaculatory apodeme that is generally well developed, at least as long as the phallapodeme, but this character state is also present in *Planinasus mcalpineorum* sp. n. (the *nigrifacies* group). The presence of a basal process on the surstylus that bears a setula distinguishes the *nigritarsus* group from most other groups of *Planinasus*, but it is also present in *Planinasus kotrbae*.

### 10. Planinasus argentifacies sp. n.

urn:lsid:zoobank.org:act:26F3D1E7-8710-4181-AE7B-A88583F33D60

http://species-id.net/wiki/Planinasus_argentifacies

Figures 53 –57

#### Description of male.

Moderately small flies, body length 2.10–2.75 mm.

*Head*: Head ratio 0.51–0.54; frons generally brownish black to black, mostly moderately microtomentose, dull to faintly subshiny, except for densely microtomentose, velvet-like, anterior margin, including area laterad of antennal bases; frons wider than long, frontal ratio 0.49–0.53; interfrontal seta shallowly curved, elongate, length subequal to length of lateral vertical seta. Antenna mostly yellowish orange, especially medially and ventrally; dorsum of pedicel and basal flagellomere brownish to blackish; basal flagellomere moderately long, length conspicuously greater than width at base, tapered to moderately acute point at apex, dorsal and ventral margins nearly straight, at most very shallowly curved; pedicel with ventral projection short, not extended anteriorly much beyond dorsal margin, bearing moderately long, ventroapical seta (not extended to apex of basal flagellomere); arista bearing 13–14 dorsal rays, 3–4 ventral rays. Face comparatively wide, facial ratio 0.58–0.62; dorsad of transverse carina moderately microtomentose, subshiny, mostly blackish brown, but becoming brownish to yellowish toward antennal grooves; ventrad of transverse carina densely microtomentose, carina and immediately ventrad blackish brown, thereafter ventrally densely silvery white to slightly yellowish along peristomal margin, sericeous; large facial setae arranged in a more-or-less single transverse row of about 8 setae, medial pair approximate, porrect to shallowly ventroclinate, next seta shallowly curved dorsally, lateral 2 setae ventroclinate. Clypeus and palpus brownish black; clypeus variable, all yellow, two-toned, to all black with some silvery white microtomentum.

*Thorax*: Mesonotum generally brownish black, faintly bluish black to black, somewhat sparsely microtomentose, subshiny; postpronotum, notopleuron, dorsal portion of anepisternum blackish brown; area from postpronotum and through notopleuron mostly bare to finely microtomentose, subshiny to shiny; pleural areas finely microtomentose, mostly dull, blackish brown, becoming more grayish brown ventrally, especially on katepisternum; anepisternum mostly bare but with 3–4 longer setulae along posterior margin; katepisternum generally setulose, bearing 2 setae toward dorsal margin. Wing without pattern, generally infumate, often slightly more so anteriorly, base and posterior margin slightly more hyaline. Coxae and trochanters grayish brown, yellowish brown to yellow; femora and tibiae uniformly brownish black, bases sometimes partially yellow; forefemur lacking a preapical annulus; foretarsus blackish brown dorsally, yellowish ventrally; mid- and hindtarsi mostly yellowish; apical 2–3 tarsomeres of all legs becoming darker; forefemur bearing 1 seta at apical 1/3 along posteroventral surface.

**Figures 53–56.**
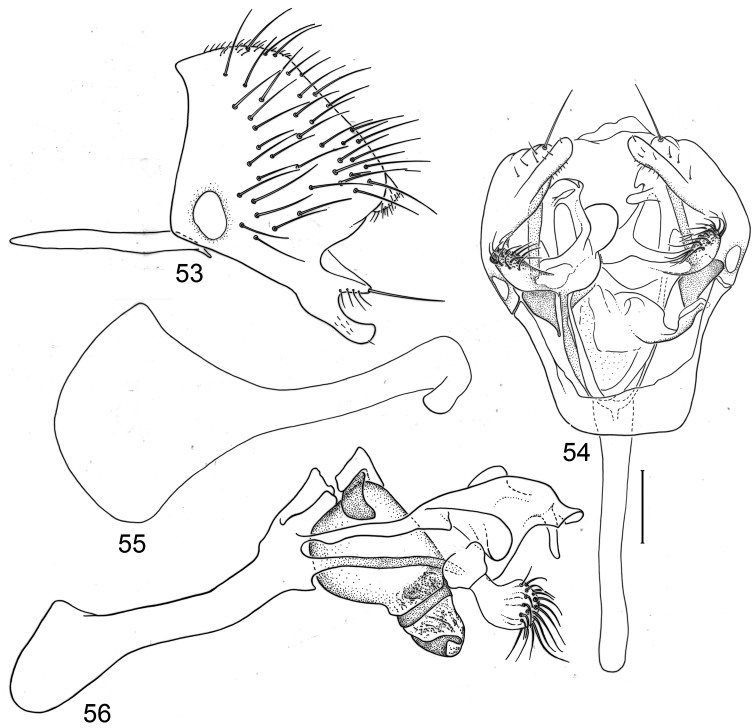
Illustrations of *Planinasus argentifacies* sp. n. (male). **53** epandrium, surstylus, and hypandrium, lateral view **54** structures of internal male terminalia, ventral view **55** ejaculatory apodeme, lateral view **56** internal structures of male terminalia, lateral view. Scale bar = 0.1 mm.

*Abdomen*: Uniformly blackish brown, mostly dull to faintly subshiny, moderately invested with microtomentum. Male abdomen: Tergites 1+2–6 well developed, lengths of tergites 3–6 subequal; tergite 7 narrow; sternite 3 only slightly wider than long, posterior margin nearly straight; sternite 4 rectangular with width about 1.5X length; sternite 5 rectangular with width about twice length, becoming slighter wider posteriorly, posterior margin of sclerotized portion with moderately broad, shallow V-shaped emargination on medial portion; sternite 6 apparently absent; sternite 7 narrow, band-like, forming an annulus with tergite 7. Male terminalia (Figs 53–56): Epandrium in lateral view (Fig. 53) broadly trapezoidal, about as wide as high, narrowed dorsally, anterior margin shallowly emarginate, posterior margin straight; surstylus length slightly less than half length of epandrium, extended from ventral margin of epandrium in nearly oblique alignment with it, in lateral view (Fig. 53), moderately wide at base, bifurcate apically with posterior lobe pointed and bearing an apical seta, deeply rounded, relatively narrowed emargination with some setulae, length of robustness of anterior lobe of bifurcation greater than posterior lobe, more robustly developed, apex truncate and slightly dilated; hypandrium in ventral view (Fig. 54) angularly U-shaped, arms narrow, anterior margin straight, robustly developed; postgonite in ventral view (Fig. 54) robust, length more than twice width, with densely setulose lobe, lobe in lateral view (Fig. 56) ovate, apex expanded, bearing more than 20 setulae; phallus in ventral view forming complex, convoluted structure with pre- and postgonites, in lateral view (Fig. 56) with narrow, bar-like processes; phallapodeme in lateral and ventral views (Figs 54, 56) elongate, in ventral view (Fig. 54) parallel sided, bluntly rounded apically, in lateral view with basal 2/3 parallel sided, slightly angulate, thereafter apically enlarged with subapical, angulate, shallow projection, apex rounded; ejaculatory apodeme enlarged, length subequal to that of phallapodeme, basal half relatively slender, almost parallel-sided, apical 1/3 greatly expanded, fan-like.

**Figure 57.**
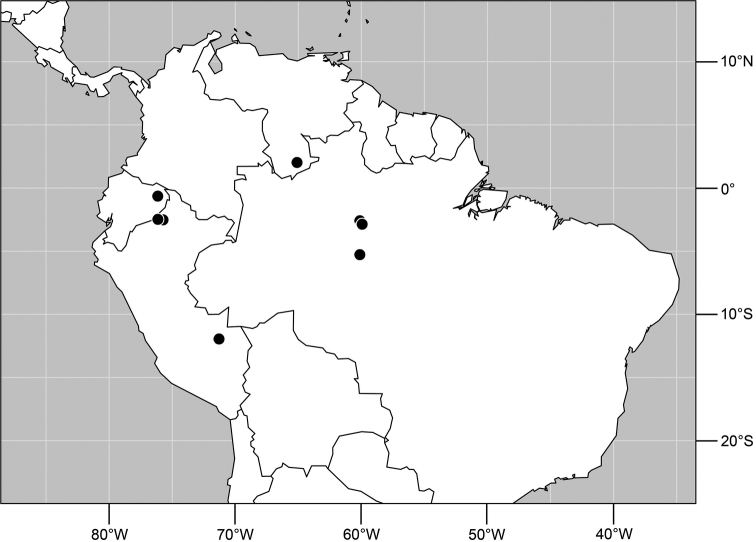
Distribution of *Planinasus argentifacies* sp. n.

#### Description of female.

As in male except as follows: Head generally narrower, head ratio 0.65–0.67; frontal ratio 0.55–0.56; facial ratio 0.35–0.45.

#### Type material.

The holotype male is labeled “PERU.Madre de Dios: Manu, Rio Manu, 250 m[,] Pakitza, 12°7'S, 70°58'W [*sic*; 11°56.6'S, 71°16.9'W], 9–23 Sep 1988[,] Amnon Freidberg/USNM ENT 00118286 [plastic bar code label]/**HOLOTYPE** ♂ *Planinasus argentifacies* Mathis & Rung USNM [red].” The holotype is double mounted (minuten pin in a plastic block), is in excellent condition, and is held in trust at the USNM for eventual deposit in Peru. Four paratypes (3♂, 1♀; USNM) bear the same label data as the holotype. Other paratypes are as follows: *PERU. Madre de Dios*: Río Manu, Pakitza (5 km E; Aguajal; 11°58.2'S, 71°17'W; 250 m), 19 Sep 1988, A. Freidberg (1♂, 2♀; USNM).

#### Type locality.

Peru. Madre de Dios: Río Manu, Pakitza (11°56.6'S, 71°16.9'W; 250 m).

#### Other specimens examined.

*BRAZIL. Amazonas*: Novo Aripuaná, Reserva Soka (05°15.9'S, 60°07.1'W; Malaise trap), 28 Apr-5 Mar 1999, R. J. Leite, R. A. Rocha, J. Vidal (2♂; INPA); Reserva Cuieiras (02°35.2'S, 60°07.2'W; 110 m), 8 May 2010, D. and W. N. Mathis (4♂, 2♀; INPA, USNM); Reserva Florestal Adolpho Ducke (02°55.8'S, 59°58.5'W; 40 m), 5 May-7 Nov 2008, 2010, D. and W. N. Mathis (3♂, 3♀; INPA, USNM); Reserva Florestal Adolpho Ducke, Igarapé Barro Branco (02°58.1'S, 60°0.3'W; 40 m; Malaise and suspension traps), 11 Apr-16 Dec 2004, A. Henriques (5♂, 2♀; INPA); Reserva Florestal Adolpho Ducke, Igarapé Tinga (02°55.8'S, 59°58.5'W; Malaise trap), 10–20 May 2004, A. Henriques (1♂; INPA); Sítio Vida Tropical (02°51.9'S, 59°55.9'W; 60 m), 5 May 2010, D. and W. N. Mathis (1♂, 2♀; INPA, USNM).

*ECUADOR. Orellana*: Rio Tiputini Biodiversity Station (0°38.2'S, 76°8.9'W), 12–26 Aug 1999, A. Baptista, M. Kotrba, W. N. Mathis (10♂, 7♀; USNM, ZSMC).

*PERU. Loreto*: Campamento San Jacinto (02°30.8'S, 75°43.5'W; 175–215 m), 11 Jul 1993, R. Leschen (2♀; DEBU); Teniente Lopez (1.5 km N; 02°28.5'S, 76°08.1'W; 230–305 m), 22 Jul 1993, R. Leschen (1♀; DEBU).

*VENEZUELA. Amazonas*: Rio Mavaca Camp (02°02'N, 65°06'W; 150 m), 16–27 Mar 1989, D. A. Grimaldi (1♂; AMNH).

#### Distribution

(Fig. 57). *Neotropical*: Brazil (Amazonas), Ecuador (Orellana), Peru (Loreto, Madre de Dios), and Venezuela (Amazonas).

#### Etymology.

The specific epithet, *argentifacies*, if of Latin derivation and refers to the distinctive, silvery microtomentum on the ventral one-third of the face.

#### Remarks.

Structures of the male terminalia of this species and those of *Planinasus nigritarsus* are very similar, and based on these morphological characters alone, we would have suggested that these two species are conspecific. There are external characters (see diagnosis and key) that clearly distinguish between these two species, however, and based on these, we conclude that they represent closely related but separate species.

### 11. Planinasus insulanus sp. n.

urn:lsid:zoobank.org:act:1DB5CFBA-9C02-48DB-8C25-8112EFBBF539

http://species-id.net/wiki/Planinasus_insulanus

**Figures 58–59.**
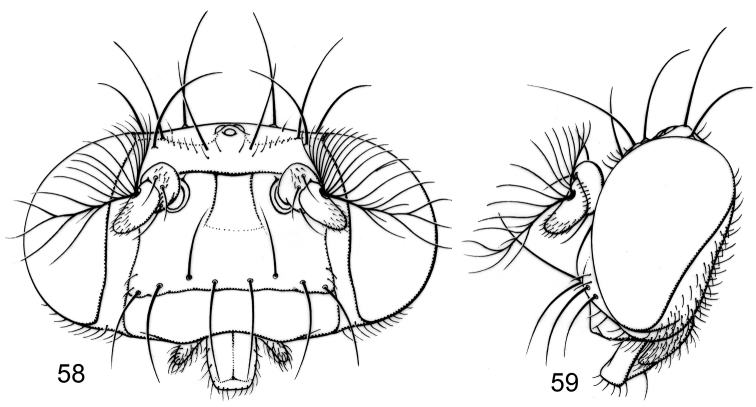
Illustrations of *Planinasus insulanus* sp. n. (male). **58** head, anterior view **59** same, lateral view.

Figures 58 [Fig F24] –64

#### Description of male.

Small to moderately small flies, body length 1.60–2.80 mm.

*Head* (58–59): Head ratio 0.52–0.54; frons generally brownish black to black, mostly bare, shiny, except for microtomentose, velvety-appearing, anterolateral angles, latter setulose; frons conspicuously wider than long, frontal ratio 0.34–0.37; interfrontal seta shallowly curved, elongate, length subequal to length of lateral vertical seta. Antenna mostly yellowish orange, especially medially and ventrally; pedicel brown laterally, mostly yellowish medially, dorsum brown; basal flagellomere moderately long, length conspicuously greater than height at base, tapered to moderately acute point at apex, both dorsal and ventral margins curved; pedicel with ventral projection short, not extended anteriorly much beyond dorsal margin, bearing long, ventroapical seta (extended slightly beyond apex of basal flagellomere), 1 dorsomedial seta and 1 medial seta; arista bearing 13–14 dorsal rays, 3–4 ventral rays. Face comparatively wide, facial ratio 0.64–0.68; dorsad of transverse carina moderately microtomentose, appearing subshiny, mostly blackish brown, but antennal grooves yellowish and with silvery white microtomentum immediately dorsal of transverse carina; ventrad of transverse carina densely microtomentose, silvery white, sericeous; large facial setae arranged in a single transverse row of about 8 setae, medial pair of setae approximate, ventroclinate, next seta curved dorsally, lateral 2 setae ventroclinate. Clypeus and palpus brownish black; clypeus with some silvery white microtomentum.

*Thorax*: Mesonotum generally brownish black to black, sparsely microtomentose, subshiny to shiny; postpronotum brown with some yellowish coloration around margin; area from postpronotum and through notopleuron mostly bare, finely microtomentose, dull; pleural areas finely microtomentose, dull, blackish brown; anepisternum mostly bare but with several setulae dorsally and posteriorly, 3–4 setulae along posterior margin longer than others; katepisternum generally setulose, bearing 2 setae toward dorsal margin. Wing without pattern, generally infumate, slightly more so anteriorly, base hyaline. Coxae and trochanters yellowish brown to brown; femora and tibiae uniformly brownish black; forefemur lacking a preapical annulus; tarsi mostly yellowish, apical 2–3 tarsomeres becoming darker; forefemur bearing 1 seta at apical 1/3 along posteroventral surface.

**Figures 60–63. F24:**
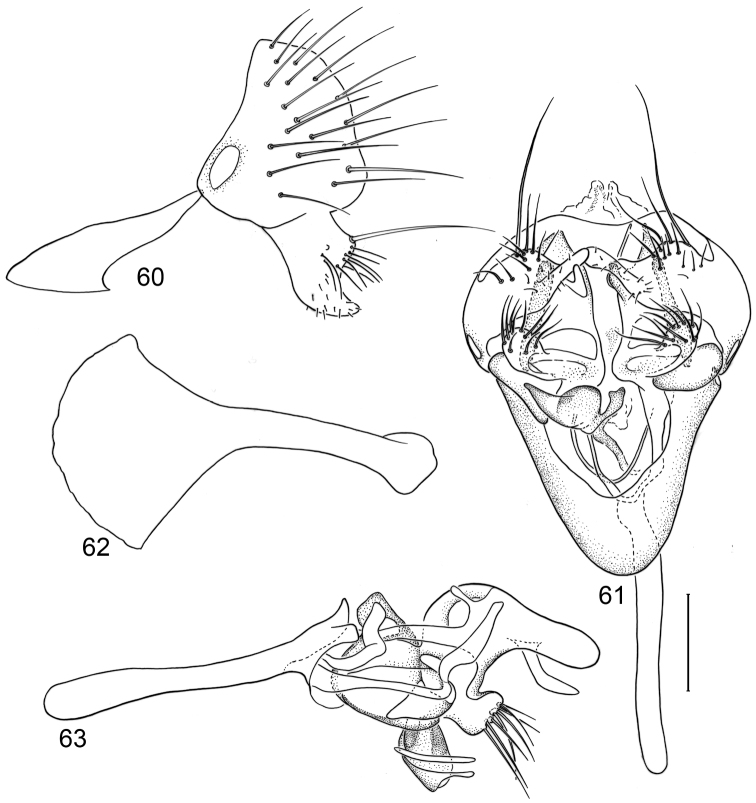
Illustrations of *Planinasus insulanus* sp. n. (male). **60** epandrium, surstylus, lateral view **61** structures of internal male terminalia, ventral view **62** ejaculatory apodeme, lateral view **63** internal structures of male terminalia, lateral view. Scale bar = 0.1 mm.

*Abdomen*: Uniformly blackish brown, mostly dull to faintly subshiny, moderately invested with microtomentum. Male abdomen: Tergites 1+2–6 well developed, lengths of tergites 3–6 subequal; tergite 7 narrow; sternites 3, 4, 5 generally as rectangular plates, slightly wider than long, lateral margins shallowly arched; sternite 5 rectangular, width twice length, posterior margin with medial notch in sclerotized portion, bearing row of setulae along posterior margin; sternite 6 apparently absent; sternite 7 narrow, band-like, forming an annulus with tergite 7. Male terminalia (Figs 60–63): Epandrium in lateral view (Fig. 60) roughly trapezoidal, higher than wide, narrowed dorsally, anterior margin shallowly emarginate, posterior margin sinuous; surstylus length slightly more than half length of epandrium, extended from posteroventral margin of epandrium in nearly oblique alignment with it, in lateral view (Fig. 60) wide, robustly developed basally, apical half abruptly tapered, forming a sub-basal posterior angle before posteroventral, moderately deep emargination, forming a tapered, pointed shallowly curved apex, bearing an elongate seta at sub-basal angle and numerous setulae along emargination; hypandrium in ventral view (Fig. 61) robustly developed, rounded V-shaped, anterior angle rounded; postgonite in ventral view (Fig. 61) robust, bearing, ovate lobe invested with numerous setulae, in lateral view (Fig. 63) with lobe truncate apically, bearing many long setulae; phallus in ventral view forming complex, convoluted structure with pre- and postgonites, in lateral view (Fig. 63) with narrow, bar-like processes; phallapodeme in lateral and ventral views (Figs 61, 63) elongate, parallel sided, bluntly rounded apically; ejaculatory apodeme in lateral view (Fig. 62) enlarged, length subequal to that of phallapodeme, basal half relatively slender, almost parallel-sided, apical 1/3 greatly expanded, fan-like.

**Figure 64.**
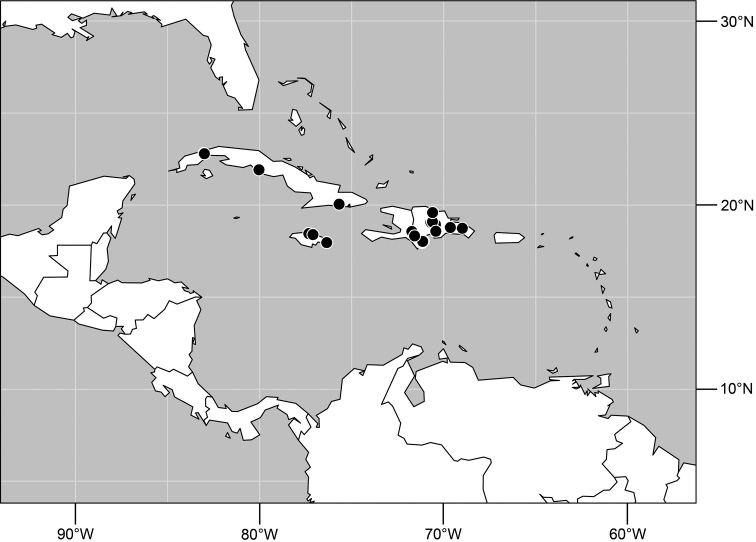
Distribution of *Planinasus insulanus* sp. n.

#### Description of female.

As in male except as follows: Head generally narrower, head ratio 0.60–0.62; frontal ratio 0.51–0.54; facial ratio 0.26–0.29.

#### Type material.

The holotype male is labeled “**DOMINICAN R**[E]**p**[UBLIC]. LaVega: n[ea]r.Jarabacoa, Salto Guasara, 19°04.4'N, 70°42.1'W, 680m, 9May 1995,Wayne N.Mathis/USNM ENT 00136283 [plastic bar code label]/**HOLOTYPE** ♂ *Planinasus insulanus* Mathis & Rung USNM [red].” The holotype is double mounted (minuten pin), is in excellent condition, and is deposited in the USNM. Paratypes are as follows: *DOMINICAN REPUBLIC. Barohona*: Baoruco (8.3 km S, 18°01.9'N, 71°08.4'W), 15 May 1995, W. N. Mathis (2♀; USNM); La Ciénaga (just S; 18°02.9'N, 71°06.4'W; 30 m; swept near stream), 3 Aug 1991, D. A. Grimaldi, J. Stark (1♂, 4♀; AMNH); Ojeda (17°58.2'N, 71°10.6'W), 22 Mar 1999, W. N. Mathis (6♂, 10♀; USNM); Paraíso (5 km N; 18°01.5'N, 71°11.6'W; 150 m), 21 Mar 1999, W. N. Mathis (4♂, 2♀; USNM); Paraíso (7 km N; 18°03'N, 71°11'W; 200 m), 27 Nov–4 Dec 1991, L. Masner, S. Peck (1♀; DEBU); San Rafael (18°01.9'N, 71°08.4'W), 22 Mar 1999, W. N. Mathis (4♂, 2♀; USNM). *El Seibo*: El Seibo (5 km E, 18°44.7'N, 68°59.2'W, 120 m), 12 May 1995, W. N. Mathis (1♀; USNM). *Independencia*: La Descubierta (18°34.1'N, 71°43.8'W), 25 Mar 1999, W. N. Mathis (1♂, 3♀; USNM); Puerto Escondido (18°19.6'N, 71°35'W; 1370 m), 24 Mar 1999, W. N. Mathis (4♂, 7♀; USNM). *La Vega*: Bonao (21 km W; 18°56.6'N, 70°27'W; 1036 m), 2 Aug 1991, D. A. Grimaldi, J. Stark (6♂, 5♀; AMNH); Jarabacoa (1–2 km S; 19°06.9'N, 70°37'W; 520 m), 8–21 May 1995, 1998, D. and W. N. Mathis (2♀; USNM); Jarabacoa (near), Salto Guasara (near, 19°04.4'N, 70°42.1'W; 680 m), 9 May 1995, W. N. Mathis (1♂; USNM); Jarabacoa (5 km S, 19°04.4'N, 70°36.5'W; 640 m), 8–20 May 1995, W. N. Mathis (1♂, 5♀; USNM). *Monte Plata*: Bayaguana (9 km N; 18°47'N, 69°38'W; 91 m; on fungus), 8 Aug 1991, D. A. Grimaldi, J. Stark (3♂, 6♀; AMNH). *Peravia*: San José Ocoa (10 km NE; 18°35'N, 70°25.6'W), 21 May 1998, D. and W. N. Mathis (12♂, 5♀; USNM). *Puerto Plata*: Sonador (19°35.9'N, 70°36.2'W; 440 m), 18 May 1995, W. N. Mathis (4♂, 2♀; USNM).

#### Type locality.

Dominican Republic. La Vega: near Jarabacoa, Salto Guasara (19°04.4'N, 70°42.1'W, 680 m).

#### Other specimens examined.

*CUBA. Cienfuegos*: Topes de Collantes (5 km WNW, 21°56.5'N, 80°02.3'W; 600 m), 11 Dec 1994, W. N. Mathis (3♂, 3♀; USNM). *Pinar del Rio*: Soroa (2 km NW, 22°48.6'N, 83°01.2'W), 4–5 Dec 1995, W. N. Mathis (10♂, 5♀; USNM); Soroa (22°47.7'N, 83°W), 4–6 Dec 1995, W. N. Mathis (2♂, 3♀; USNM). *Sancti Spiritus*: Topes de Collantes (21°55.2'N, 80°02.3'W; 350 m), 10 Dec 1995, W. N. Mathis (5♂, 4♀; USNM). *Santa Clara*: San Juan Mountains (20°03'N, 75°41'W), Jan-Feb 1927, C. T. and B. B. Brues (1♂, 1♀; USNM).

*JAMAICA. St. Ann*: Runaway Bay (18°27.4'N, 77°19.6'W; stream bed), Feb 1969, W. W. Wirth (1♀; USNM). Ocho Rios (18°23.9'N, 77°6.2'W), Jul 1958, W. B. Heed, M. Wasserman (1♂, 1♀; USNM). *St. Thomas*: near Bath (17°57.9'N, 76°21'W), Feb 1956, W. B. Heed (1♀; USNM).

#### Distribution

(Fig. 64). *Neotropical*: West Indies. Cuba (Cienfuegos, Pinar del Rio, Sancti Spiritus, Santa Clara), Dominican Republic (Barahona, El Seibo, Independencia, La Vega, Monte Plata, Peravia, Puerto Plata), Jamaica (St. Ann).

#### Etymology.

The species epithet, *insulanus*, is of Latin derivation, meaning island and refers to the occurrence of this species on West Indian islands.

#### Remarks.

Variation in wing coloration is particularly evident in specimens of this species and may be related to the age of specimens, older specimens having darker wings, but also to underlying genetic variation. The shape of the surstylus and hypandrium readily distinguish this species from congeners.

### 12. Planinasus nigritarsus sp. n.

urn:lsid:zoobank.org:act:BD9CEBD6-C6B1-492B-A19B-D4FC36C7595E

http://species-id.net/wiki/Planinasus_nigritarsus

Figures 65 –69

#### Description of male.

Moderately small flies, body length 2.10–2.80 mm.

*Head*: Head ratio 0.56–0.60; frons generally brownish black to black, mostly bare, shiny, except for microtomentose, velvety-appearing, anterolateral angles and overcut, anterior margin, anterolateral area setulose; frons conspicuously wider than long, frontal ratio 0.47–0.50; interfrontal seta shallowly curved, elongate, length subequal to length of lateral vertical seta. Antenna mostly yellowish orange, especially medially and ventrally; pedicel mostly yellowish, especially medially, brown dorsally; basal flagellomere moderately long, length conspicuously greater than height at base, tapered to moderately acute point at apex, both dorsal and ventral margins curved; pedicel with ventral projection short, not extended anteriorly much beyond dorsal margin, bearing long, ventroapical seta (extended slightly beyond apex of basal flagellomere), 1 dorsomedial seta and 1 medial seta; arista bearing 13–14 dorsal rays, 3–4 ventral rays. Face comparatively wide, facial ratio 0.60–0.64; dorsad of transverse carina moderately microtomentose, appearing subshiny, mostly blackish brown, but antennal grooves yellowish and with silvery white microtomentum immediately dorsal of transverse carina; ventrad of transverse carina densely microtomentose, silvery white, sericeous; large facial setae arranged in a single transverse row of about 8 setae, medial pair of setae approximate, ventroclinate, next seta curved dorsally, lateral 2 setae ventroclinate. Clypeus and palpus brownish black; clypeus with some silvery white microtomentum.

*Thorax*: Mesonotum generally brownish black to black, sparsely microtomentose, subshiny to shiny; postpronotum brown with some yellowish coloration around margin; area from postpronotum and through notopleuron mostly bare, finely microtomentose, dull; pleural areas finely microtomentose, dull, blackish brown; anepisternum mostly bare but with several setulae dorsally and posteriorly, 3–4 setulae along posterior margin longer than others; katepisternum generally setulose, bearing 2 setae toward dorsal margin. Wing without pattern, generally infumate, slightly more so anteriorly, base hyaline. Coxae and trochanters whitish yellow to yellow; femora and tibiae uniformly brownish black; base of femora yellowish; forefemur with a preapical, pale area medially; foretarsus mostly dark, especially dorsally, brownish to blackish, paler ventrally, yellowish; mid- and hindtarsi mostly yellowish, apical 2–3 tarsomeres becoming darker; forefemur bearing 1 seta at apical 1/3 along posteroventral surface.

**Figures 65–68.**
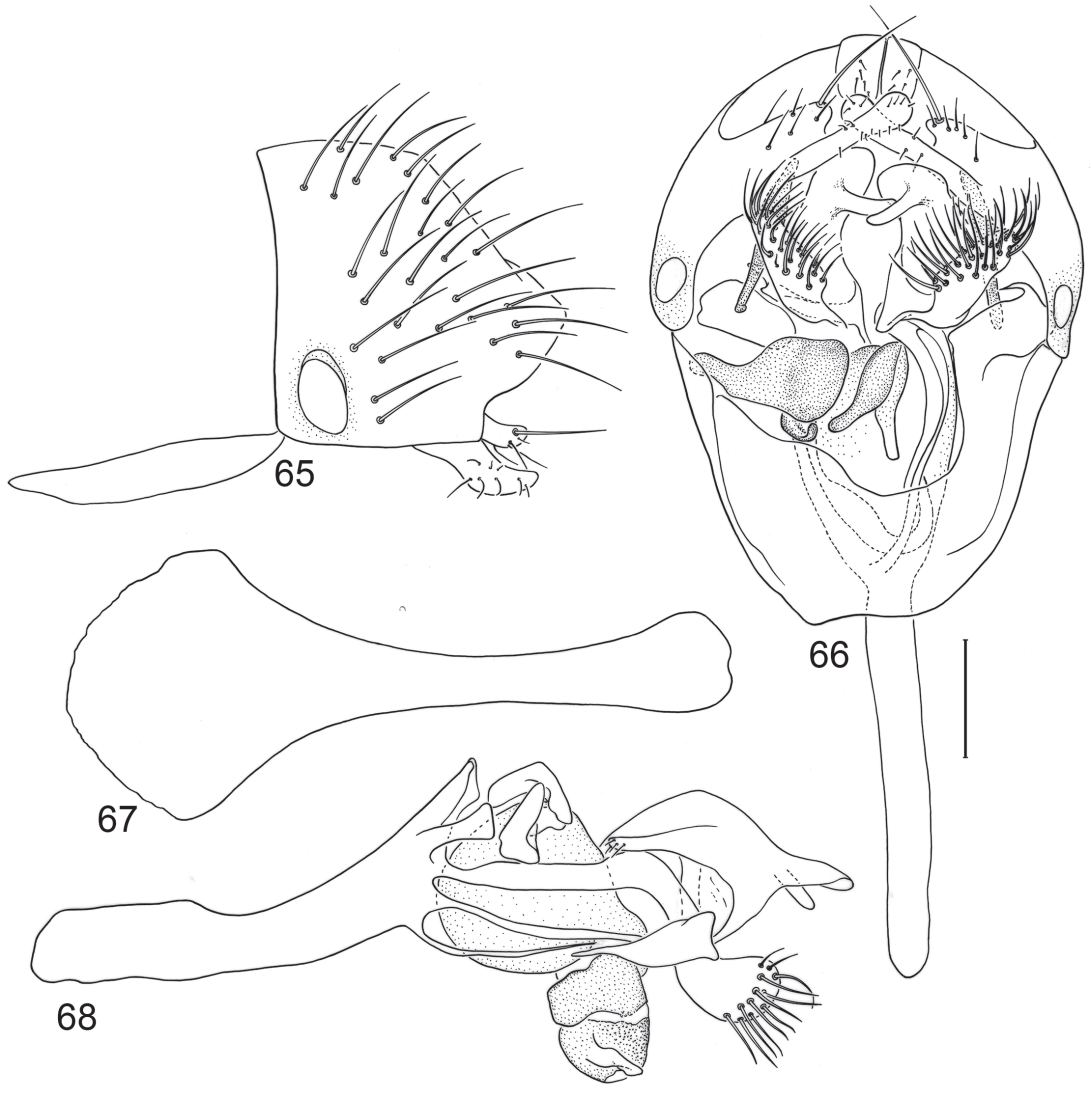
Illustrations of *Planinasus nigritarsus* sp. n. (male). **65** epandrium, surstylus, lateral view **66** structures of internal male terminalia, ventral view **67** ejaculatory apodeme, lateral view **68** internal structures of male terminalia, lateral view. Scale bar = 0.1 mm.

*Abdomen*: Uniformly blackish brown, mostly dull to faintly subshiny, moderately invested with microtomentum. Male abdomen: Tergites 1+2–6 well developed, lengths of tergites 3–6 subequal; tergite 7 narrow; sternite 3 only slightly wider than long, posterior margin nearly straight; sternite 4 rectangular with width about 1.5X length; sternite 5 rectangular with width about twice length, becoming slighter wider posteriorly, posterior margin of sclerotized portion nearly straight; sternite 6 apparently absent; sternite 7 narrow, band-like, forming an annulus with tergite 7. Male terminalia (Figs 65–68): Epandrium in lateral view (Fig. 65) broadly rectangular on anterior half, greatest width as wide as high, narrowed dorsally, anterior margin very shallowly emarginate, posterior margin straight, angled posteriorly, forming a pointed, broadly based projection; surstylus length 1/3 length of epandrium, extended from posteroventral margin of epandrium in nearly oblique alignment with it, in lateral view (Fig. 65) relatively short, bilobed, dorsal lobe smaller, length half that of ventral lobe, bearing apical, elongate setulae, ventral lobe as a parallelogram, more robustly developed than dorsal lobe, bearing several shorter setulae; hypandrium in ventral view (Fig. 66) angularly and robustly U-shaped, arms only slightly narrower than anterior margin, anterior margin nearly straight, robustly developed; postgonite in ventral view (Fig. 66) robust, bearing densely setulose lobe, length of lobe more than twice width, , lobe in lateral view (Fig. 68) ovate, apex expanded, bearing more than 20 setulae; phallus in ventral view forming complex, convoluted structure with pre- and postgonites, in lateral view (Fig. 68) with narrow, bar-like processes; phallapodeme in lateral and ventral views (Figs 68, 66) elongate, in ventral view (Fig. 66) parallel sided, bluntly rounded apically, in lateral view slightly curved, especially along dorsal margin, slightly dilated basally, apical 1/3 parallel sided; ejaculatory apodeme in lateral view (Fig. 67) enlarged, longer than phallapodeme, basal half relatively slender, almost parallel-sided, apical 1/3 greatly expanded, fan-like.

**Figure 69.**
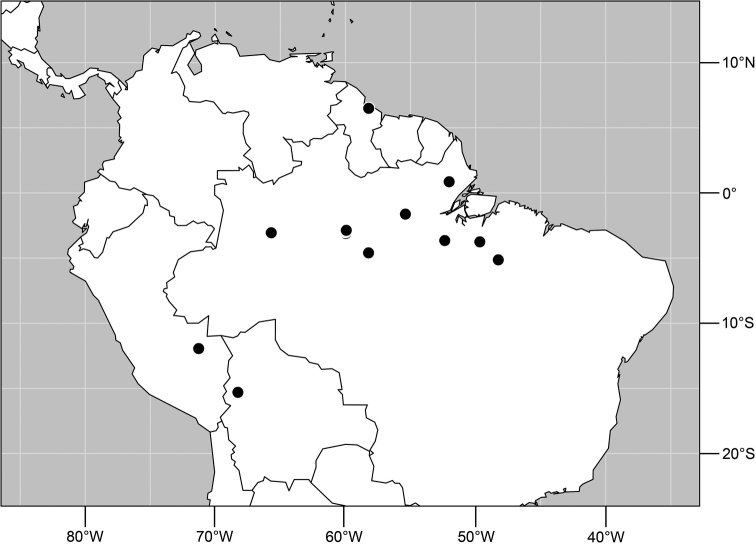
Distribution of *Planinasus nigritarsus* sp. n.

#### Description of female.

As in male except as follows: Head generally narrower, head ratio 0.60–0.62; frontal ratio 0.61–0.64; facial ratio 0.27–0.31.

#### Type material.

The holotype male is labeled “**GUYANA.** CEIBA (ca. 40 km S Georgetown)[,] 06°29.9'N, 58°13.1'W[,] 21 April 1995[,] Wayne N. Mathis/USNM ENT 00134161 [plastic bar code label]/**HOLOTYPE** ♂ *Planinasus nigritarsus* Mathis & Rung USNM [red]”. The holotype is double mounted (minuten in a block of plastic elastomere), is in excellent condition, and is held in trust at the USNM for eventual deposit in Guyana. Nineteen paratypes (13♂, 6♀; USNM) bear the same label data as the holotype. Other paratypes are as follows: Same locality as the holotype but with dates from 13 Apr-28 Aug 1994, 1997 (17♂, 5♀; USNM).

#### Type locality.

Guyana. Conservation of Ecological Interactions and Biotic Associations (CEIBA; ca. 40 km S Georgetown; 06°29.9'N, 58°13.1'W).

#### Other specimens examined.

*BOLIVIA. La Paz*: Mapiri (5 km W; Arroyo Tuhiri; 15°17.8'S, 68°15.6'W; 750 m), 16–19 Mar 2001, S. D. Gaimari, W. N. Mathis (3♂; USNM).

*BRAZIL. Amapá*: Serra do Navio (0°52'N, 52°01.5'W; Malaise trap), 15–17 May 1989, N. Bittencourt (1♀; INPA). *Amazonas*: Manaus, Universidade Federal do Amazonas (03°05.9'S, 59°58.2'W; 50 m), 7 May 2010, D. and W. N. Mathis (1♂; USNM); Reserva da Campina, Rio Abacaxis (04°35.8'S, 58°13.2'W), 30–31 May 2008, J. A. Rafael (1♀; INPA); Reserva Florestal Adolpho Ducke (02°55.8'S, 59°58.5'W; 40 m), 5 May 2010, D. and W. N. Mathis (1♂; USNM); Reserva Florestal Adolpho Ducke, baixio trilha leste-oeste (02°55.8'S, 59°58.5'W; suspension trap), Mar 2004, G. Freitas, J. Vidal (1♂, 1♀; INPA); Reserva Florestal Adolpho Ducke, Platô, trilha leste-oeste (02°55.8'S, 59°58.5'W; suspension trap), 14 Feb-6 Mar 2007, G. Freitas (1♀; INPA); Reserva Florestal Adolpho Ducke, Igarapé Barro Branco (02°58.1'S, 60°0.3'W; Malaise trap, suspension trap at 25 m), Feb 2004, A. Henriques (2♀; INPA); Reserva Florestal Adolpho Ducke, Igarapé Tinga (02°55.8'S, 59°58.5'W; Malaise trap, suspension trap at 25 m), Mar-23 Sep 2004, A. Henriques (2♀; INPA); Sítio Vida Tropical (02°51.9'S, 59°55.9'W; 60 m), 5 May 2010, D. and W. N. Mathis (1♂; USNM); Urini (03°03'S, 65°41.7'W), 22 Jul-3 Aug 1995, N. Aguiar, P. Bűhmhein (1♀; INPA). *Maranhão*: São Pedro da Água Branca, Fazenda Santa Rosa (05°07.8'S, 48°15.2'W), 6 Dec 2001, F. L. Oliveira, J. A. Rafael, J. Vidal (1♀; INPA). *Pará*: Óbidos, Fazenda Pajurá (01°37.4'S, 55°23.2'W), 1 Sep 2001, J. A. Rafael, J. Vidal (1♂; INPA); Rio Zingu Camp (03°39'S, 52°22'W; ca. 60 km S Altamira), 2–8 Oct 1986, O. S. Flint, P. Spangler (2♀; USNM); Apr 1930, N. C. Davis (1♂; USNM); Tucurui, Rio Tocantins (03°45.1'S, 49°40.5'W), 9–11 Jun 1984, (1♀; INPA).

*PERU. Madre de Dios*: Río Manu, Pakitza (11°56.6'S, 71°16.9'W; 250 m), 9–23 Sep 1988, A. Freidberg, W. N. Mathis (6♂, 2♀; USNM).

#### Distribution

(Fig. 69). *Neotropical*: Bolivia (La Paz), Brazil (Amapá, Amazonas, Maranhão, Pará), Guyana, and Peru (Madre de Dios).

#### Etymology.

The specific epithet, *nigritarsus*, is of Latin derivation and alludes to the dark tarsomeres of the foreleg.

#### Remarks.

See “Remarks” under *Planinasus argentifacies* for a discussion on distinguishing characters as well as similar morphological characters.

##### The *nigrifacies* group

**Included species.** *Planinasus atriclypeus* sp. n., *Planinasus atrifrons* sp. n., *Planinasus flavicoxalis* sp. n., *Planinasus mcalpineorum* sp. n., and *Planinasus nigrifacies* sp. n.

**Diagnosis.** This species group is distinguished by the following combination of characters: *Head*: Interfrontal seta short, about half length of lateral vertical seta. Antennal coloration variable; pedicel with short ventral projection; basal flagellomere short, about as high as long. Large facial setae arranged in 2–3 transverse rows; face of males and females similar in shape and color. *Thorax*: Anepisternum with 1 large seta along posterior margin. Wing hyaline to faintly infumate. Forefemur lacking subapical, irregular, pale-colored annulus, bearing 2 large seta at apical 1/3 along posteroventral surface. *Abdomen*: Surstylus in nearly oblique alignment with epandrium, generally bearing a posterior process, or lobe, with a setula; postgonite generally with a digitiform lobe bearing a few setulae apically; phallus mostly membranous; ejaculatory apodeme generally greatly reduced.

**Discussion.** This species group may not be monophyletic, being based primarily on plesiomorphic characters, and comprises the “remainders” species that could not be conveniently placed in other groups. Species of this group share, with those of the *shannoni* group, and with *Planinasus obscuripennis* the greatly reduced phallapodeme (conspicuous only in *Planinasus nigrifacies* in this group) and the small number of setulae on the lobe of the postgonite (less than 6). Furthermore, in the *shannoni* and *nigrifacies* groups, the phallus is only partially sclerotized, contrasting with the heavily sclerotized phallus typical of species of the *nigritarsus* and *ambiguus* groups.

### 13. Planinasus atriclypeus sp. n.

urn:lsid:zoobank.org:act:B5A68187-07BF-46F4-807F-A7CC479CBE12

http://species-id.net/wiki/Planinasus_atriclypeus

Figures 34 70–72

#### Description of male.

Moderately small to medium-sized flies, body length 2.60–3.15 mm.

*Head*: Head ratio 0.62–0.65; frons generally uniformly brownish black to black, mostly very finely and sparsely microtomentose, subshiny to shiny, except for more densely microtomentose, anterolateral angles and slightly undercut anterior margin; anterolateral angles setulose; frons wider than long, frontal ratio 0.53–0.57; interfrontal seta shallowly curved, moderately elongate, about 2/3 length of lateral vertical seta. Antenna mostly uniformly black; some specimens with ventral portion of basal flagellomere and pedicel faintly yellowish brown; basal flagellomere moderately short, length at most only slightly greater than height at base, tapered gradually to narrowly rounded acute, dorsal and ventral margins nearly straight;; pedicel with ventral projection short, length of projection conspicuously less than length of pedicel without considering projection, bearing long, ventroapical seta (extended slightly beyond apex of basal flagellomere), 1 dorsoapical seta, 1 dorsal seta; arista bearing 13–14 dorsal rays, 3–4 ventral rays. Face comparatively narrow, facial ratio 0.34–0.37; portion dorsad of transverse carina moderately large, sparsely microtomentose, subshiny to shiny, mostly brownish to bluish black; antennal grooves especially shiny; ventrad of transverse carina more densely microtomentose, yellowish brown medially, moderately sparsely silvery yellow microtomentose, sericeous, lateral portions of face becoming bluish black; large facial setae arranged in approximately 2 transverse rows; dorsal row with 6 setae, medial pair of dorsal row moderately widely separate, shallowly dorsoclinate; next setae much shorter, shallowly ventroclinate; ventral row with 6 setae, all ventroclinate, medial pair slightly shorter than others. Clypeus brownish black to black with very sparse whitish yellow microtomentum; palpus brownish black.

*Thorax*: Mesonotum generally brownish black to deep bluish black, thinly microtomentose, subshiny to shiny; postpronotum brown, some specimens with some yellowish coloration around margin; area from postpronotum and through notopleuron at most finely and thinly microtomentose, subshiny to shiny; pleural areas finely microtomentose, subshiny, blackish brown; anepisternum mostly bare but with 1–2 setulae along posterior margin; katepisternum generally setulose, bearing 2 setae toward dorsal margin. Wing without pattern, generally infumate. Coxae and trochanters yellow; Femora and tibiae mostly brownish black; forefemur lacking a preapical annulus; tarsi mostly yellowish basally, apical 3–4 tarsomeres becoming darker; forefemur bearing 1 seta at apical 1/3 along posteroventral surface.

**Figures 70–72.**
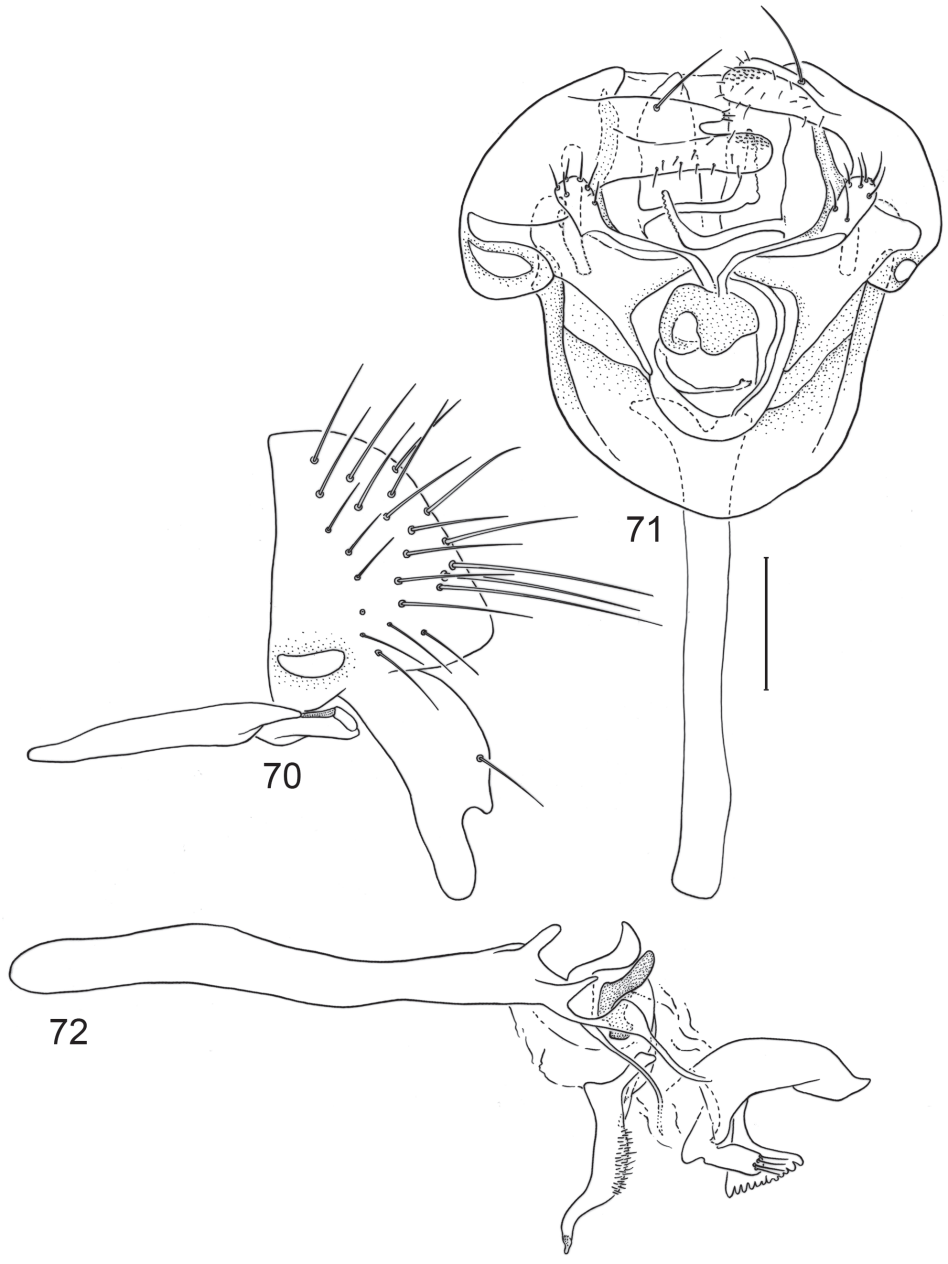
Illustrations of *Planinasus atriclypeus* sp. n. (male). **70** epandrium, surstylus, hypandrium, and pregonite, lateral view **71** structures of internal male terminalia, ventral view **72** internal structures of male terminalia, lateral view, lateral view. Scale bar = 0.1 mm.

*Abdomen*: Uniformly blackish brown, mostly dull to faintly subshiny, moderately invested with microtomentum. Male abdomen: Tergites 1+2–6 well developed, lengths of tergites 3–6 subequal; tergite 7 narrow; sternites 3, 4, 5 generally as rectangular plates, slightly wider than long, lateral margins shallowly arched; sternite 5 with lateral margins nearly straight, posterior margin of sclerotized portion nearly straight; sternite 6 apparently absent; sternite 7 very narrow, band-like, lightly sclerotized medially, forming an annulus with tergite 7. Male terminalia (Figs 70–72): Epandrium in lateral view (Fig. 70) trapezoidal, higher than wide, narrowed dorsally, anterior and posterior margins nearly straight; surstylus as long as epandrium, extended from ventral margin of epandrium in nearly straight to slightly oblique alignment with it, in lateral view (Fig. 70) elongate, robustly developed, unevenly bilobed apically, anterior lobe much larger, digitiform, rounded apically, short posterior lobe shallowly pointed, bearing medial, elongate setula; hypandrium in ventral view (Fig. 71) broadly and robustly U-shaped, arms tapered, narrowed toward attachment with epandrium, anterior portion robustly developed, anterior margin shallowly pointed; pregonite roughly triangular (Fig. 71), with anterior margin significantly receded and slightly round corners; postgonite in ventral view (Fig. 71) convoluted, bearing digitiform lobe with 6 apical and marginal setulae, in lateral view (Fig. 72) lobe 4 apical, short setulae are seen; phallus in ventral view difficult to discern, in lateral view (Fig. 72) longer than wide, slender, with pointed apex and posterior fine hairs; phallapodeme in lateral and ventral views (Figs 71–72) elongate, shallowly sinuous to nearly straight; ejaculatory apodeme greatly reduced.

#### Description of female.

As in male except as follows: Head generally slightly narrower, head ratio 0.65–68; frontal ratio 0.44–48; facial ratio 0.34–37. Face mostly uniformly bluish black, silvery white microtomentum more evident.

#### Type material.

The holotypes male is labeled “Floresta da Tijuca- BRASIL/30 XII 1991 A. BAPTISTA R. BAPTISTA em Marantaceae [date and name of plant family handwritten]/USNM ENT 00118276 [plastic bar code label]/**HOLOTYPE** ♂ *Planinasus atriclypeus* Mathis & Rung MZUSP [red]. The holotype is double mounted (glued to a paper point), is in good condition, and is deposited in the MZUSP. Eleven paratypes (5♂, 6♀; MZUSP, USNM) bear the same locality label as the holotype. Other paratypes are as follows: Brazil. Rio de Janeiro: Parque Nacional de Itatiaia, Maromba (22°29.7'S, 44°33.7'W), 13–17 Dec 1991, A. and R. Baptista (2♂, 1♀; MZUSP, USNM).

#### Type locality.

Brazil. Rio de Janeiro: Rio de Janeiro, Floresta da Tijuca (22°57.6'S, 43°16.4'W).

#### Other specimens examined.

*BRAZIL. Rio de Janeiro*: Nova Friburgo (22°17.2'S, 42°32'W), 1 May 1995, A. and R. Baptista (1ex (abdomen missing); USNM); Paineiras, Teresópolis (22°24.3'S, 42°58.3'W), 5 Feb 1995, A. and R. Baptista (1♂; USNM); Parque Estadual do Desengano (near Santa Maria Magdalena; 21°53'S, 41°55'W), 19–23 Dec 1991, A. and R. Baptista (1♂; MZUSP). *São Paulo*: Salesópolis (23°31.9'S, 45°50.8'W), 1–3 Mar 1992, A. and R. Baptista (1♂; USNM).

#### Distribution

(Fig. 34). *Neotropical*: Brazil (Rio de Janeiro, São Paulo).

#### Etymology.

The specific epithet, *atriclypeus*, is of Latin derivation and refers to the black clypeus, a diagnostic feature of this species.

#### Remarks.

**Figures 73–75.**
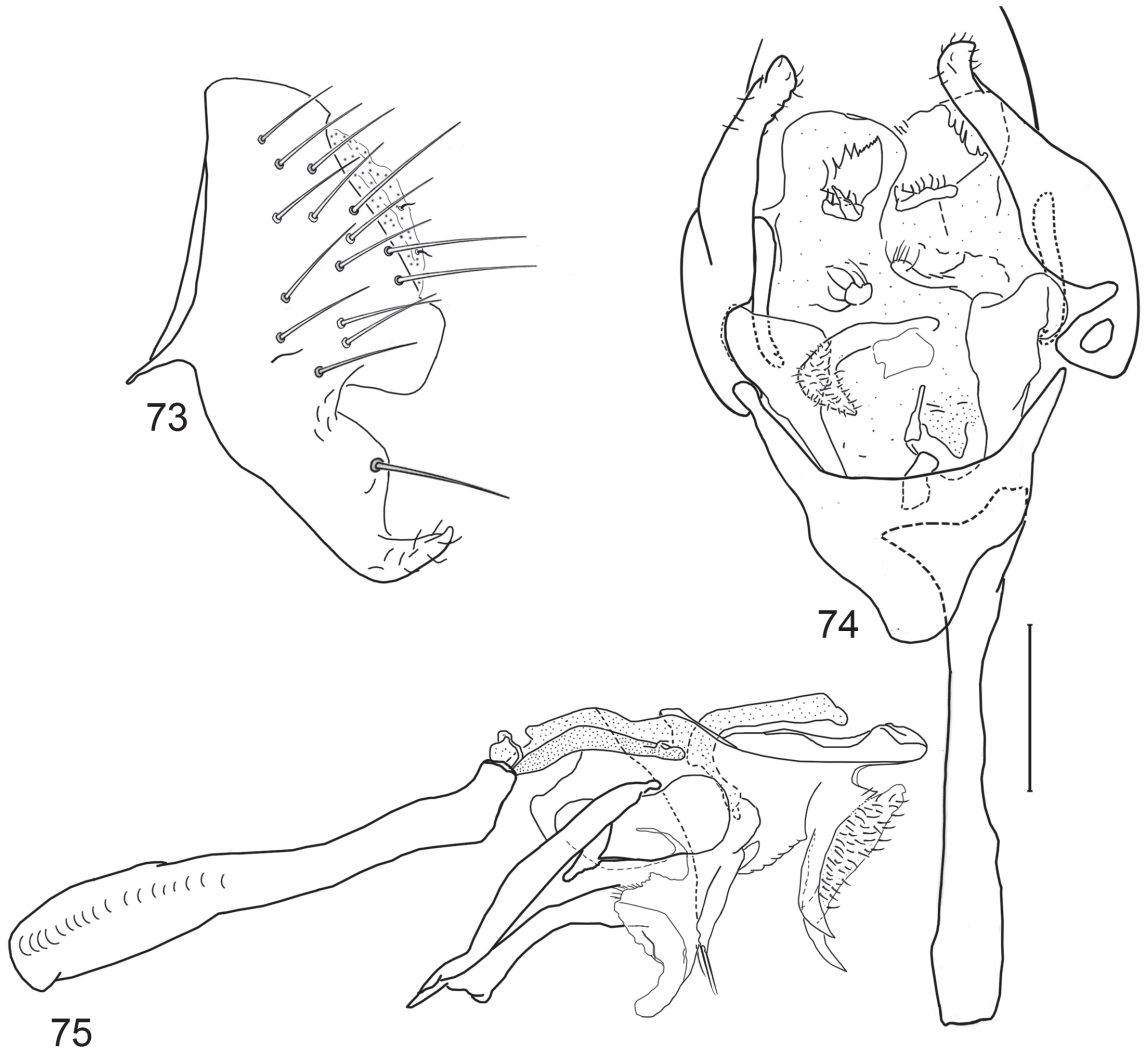
Illustrations of *Planinasus atrifrons* sp. n. (male). **73** epandrium and surstylus, lateral view **74** structures of internal male terminalia, ventral view **75** internal structures of male terminalia, lateral view. Scale bar = 0.1 mm.

This species is most similar to *Planinasus atrifrons*. Besides the characters given in the key, it can be distinguished from that species by structures of the male terminalia, particularly the unevenly bilobed surstylus, with anterior lobe much larger, digitiform, rounded apically, and a short, shallowly pointed posterior lobe. In *Planinasus atrifrons*, the surstylus is swollen medially, and the apex makes a nearly right angle with the main surstylar apex (Fig. 73).

### 14. Planinasus atrifrons sp. n.

urn:lsid:zoobank.org:act:E2390043-F256-4951-B4EF-A8DABA4D87B3

http://species-id.net/wiki/Planinasus_atrifrons

Figures 73 –76

#### Description of male.

Moderately small to medium-sized flies, body length 2.20–3.00 mm (holotype 2.38 mm).

*Head*: Head ratio 0.63; frons generally brownish black to black, mostly very finely and sparsely microtomentose, subshiny to shiny, except for more densely microtomentose, anterolateral angles and undercut anterior margin; anterolateral angles setulose; frons wider than long, frontal ratio 0.45; interfrontal seta shallowly curved, moderately elongate, about 2/3 length of lateral vertical seta. Antenna yellow to black; scape yellow medially; pedicel faintly brownish dorsally, yellowish ventrally; basal flagellomere yellowish brown, elongate, length conspicuously longer than height, tapered to moderately acute point at apex, dorsal margin shallowly depressed, ventral margin nearly straight to very shallowly arched; pedicel with ventral projection short, length of projection conspicuously less than length of pedicel without considering projection, bearing long, ventroapical seta (extended slightly beyond apex of basal flagellomere), 1 dorsoapical seta, 1 dorsal seta; arista bearing 13–14 dorsal rays, 3–4 ventral rays. Face comparatively narrow, facial ratio 0.47; portion dorsad of transverse carina moderately large, sparsely microtomentose, subshiny to shiny, mostly yellow to brownish yellow in flattened antennal grooves; ventrad of transverse carina more densely microtomentose, brown medially, silvery yellow laterally, sericeous; large facial setae arranged in 2 transverse rows; dorsal row with 6 setae, medial pair of dorsal row somewhat widely separate, shallowly dorsoclinate; next setae shorter, shallowly ventroclinate; ventral row with 6 setae, all ventroclinate, medial pair shorter than others. Clypeus brownish black to black with very sparse whitish yellow microtomentum; palpus brownish black.

*Thorax*: Mesonotum generally brownish black to deep bluish black, thinly microtomentose, subshiny to shiny; postpronotum brown, some specimens with some yellowish coloration around margin; area from postpronotum and through notopleuron at most finely and thinly microtomentose, subshiny to shiny; pleural areas finely microtomentose, subshiny, blackish brown; anepisternum mostly bare but with 1–2 setulae along posterior margin; katepisternum generally setulose, bearing 2 setae toward dorsal margin. Wing without pattern, generally infumate. Coxae, trochanters, and base of femora yellow; remainder of femora and tibiae mostly brownish black; forefemur lacking a preapical annulus; tarsi mostly yellowish basally, apical 2–3 tarsomeres becoming darker; forefemur bearing 1 seta at apical 1/3 along posteroventral surface.

*Abdomen*: Uniformly blackish brown, mostly dull to faintly subshiny, moderately invested with microtomentum. Male abdomen: Tergites 1+2–6 well developed, lengths of 3–6 subequal; tergite 7 narrow; sternites 3, 4, 5 generally as rectangular plates, slightly wider than long, lateral margins shallowly arched; sternite 5 slightly wider subposteriorly; sternites 6, 7 lacking, neither segment forming an annulus. Male terminalia (Figs 72–75): Epandrium in lateral view (Fig. 73) broadly trapezoidal, higher than wide, narrowed dorsally, anterior margin nearly straight, posterior margin straight on dorsal 2/3 thereafter ventrally forming a robust truncate extension; surstylus almost as long as epandrium, extended from ventral margin of epandrium in nearly oblique alignment with it, in lateral view (Fig. 73) elongate, robustly developed, distinctly tapered, apex recurved, anterior margin very shallowly sinuous, posterior margin swollen medially, apical 1/4 tapered to moderately acute point, partially recurved, bearing medial, elongate setula from posteromedial extension; hypandrium in ventral view (Fig. 74) moderately robustly V-shaped, arms tapered toward attachment with epandrium, anterior portion robustly developed with lateral margins sinuous, anterior margin broadly pointed; pregonite roughly triangular , with anterior margin receded and slightly round corners (Fig. 74); postgonite in ventral view (Fig. 74) convoluted, with relatively small, narrow lobe bearing 6 marginal and apical setulae, in lateral view (Fig. 75) lobe bar-like, with 3 visible apical, short setulae; phallus in J-shaped, bearing apical hairs, in lateral view (Fig. 75) obscured by the postgonites; phallapodeme in lateral and ventral views (Figs 74–75) elongate, irregularly parallel sided, truncate apically; ejaculatory apodeme greatly reduced.

**Figure 76.**
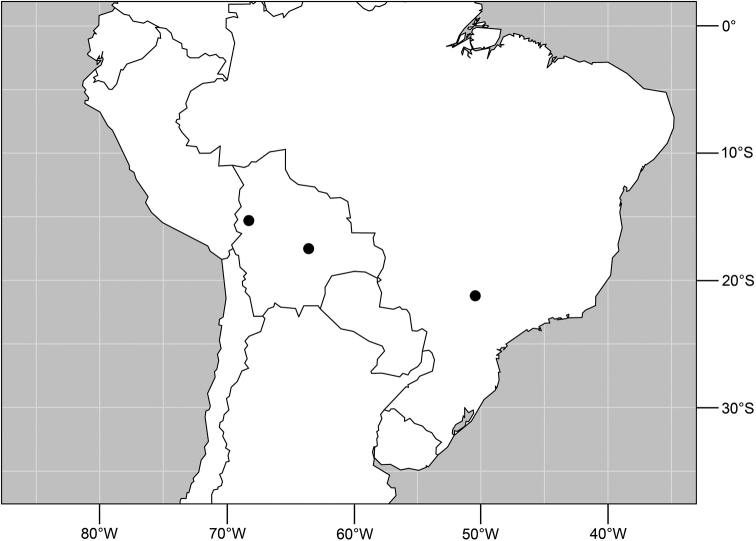
Distribution of *Planinasus atrifrons* sp. n.

#### Description of female.

As in male except as follows: Head generally slightly narrower, head ratio 0.67–69; frontal ratio 0.41–44; facial ratio 0.41–44.

#### Type material.

The holotype male is labeled “BOLIVIA: Santa Cruz Dept. Ichilo Prov. Hotel Flora Y Fauna[,] 4–6 km SSE Buena Vista[,] 17°29.95'S, 63°33.15'W[,] 400–500m[,] 11 November 2003[,] N. E. Woodley/USNM ENT 00118279 [plastic bar code label]/**HOLOTYPE** ♂ *Planinasus atrifrons* Mathis & Rung USNM [red].” The holotype is double mounted (minuten pin in a synthetic block), is in excellent condition (abdomen removed and dissected, parts in an attached microvial of glycerin), and is deposited in the USNM. A female paratype (1♀; USNM) is from: *BOLIVIA. La Paz*: Guanay (22 km SE; 15°17.8'S, 68°15.6'W; 540 m), 17 Mar 2001, W. N. Mathis.

#### Type locality.

Bolivia. Santa Cruz: Ichilo, Buena Vista (4–6 km SSE; Hotel Flora y Fauna; 17°29.95'S, 63°33.15'W; 4–500 m).

#### Other specimens examined.

*BRAZIL. São Paulo*: Araçatuba, Córrego Azul (21°12.6'S, 50°26.6'W), Mar 1947, M. P. Barretto (3♀; MZUSP).

#### Distribution

(Fig. 76). *Neotropical*: Bolivia (La Paz, Santa Cruz), Brazil (São Paulo).

#### Etymology.

The specific epithet, *atrifrons*, is of Latin derivation and refers to the black frons, a feature of this species.

#### Remarks.

See “Remarks” under *Planinasus atryclypeus* for diagnostic characters to distinguish this species from that one.

### 15. Planinasus flavicoxalis sp. n.

urn:lsid:zoobank.org:act:0C27D541-96E0-479B-ADB0-9FCC78B6A035

http://species-id.net/wiki/Planinasus_flavicoxalis

Figures 43 , 77–79

#### Description of male.

Moderately small to medium-sized flies, body length 2.55–3.25 mm.

*Head*: Head ratio 0.63–0.66; frons generally brownish black to black, mostly very finely and sparsely microtomentose, subshiny to shiny, except for more densely microtomentose, anterolateral angles and anterior margin; anterolateral angles setulose; frons wider than long, frontal ratio 0.51–0.57; interfrontal seta shallowly curved, moderately elongate, about 2/3 length of lateral vertical seta. Antenna black; basal flagellomere variable but usually short, length usually not exceeding height at base, tapered to moderately acute point at apex, both dorsal and ventral margins nearly straight to very shallowly depressed dorsally and arched ventrally; pedicel with ventral projection short, length of projection conspicuously less than length of pedicel without considering projection, bearing long, ventroapical seta (extended slightly beyond apex of basal flagellomere), 1 dorsal seta, 1 dorsomedial seta, 1 medial seta; arista bearing 13–14 dorsal rays, 3–4 ventral rays. Face comparatively narrow, facial ratio 0.46–0.52; portion dorsad of transverse carina large, sparsely microtomentose, subshiny to shiny, mostly yellow to slightly brownish yellow in flattened antennal grooves; ventrad of transverse carina more densely microtomentose, silvery yellow, sericeous; large facial setae arranged in 2 transverse rows; dorsal row with 4 setae, medial pair of dorsal row widely separate, shallowly dorsoclinate; next seta shallowly ventroclinate; ventral row with 6 setae, all ventroclinate. Clypeus yellow with very sparse whitish yellow microtomentum; palpus brownish black.

*Thorax*: Mesonotum generally brownish black to deep bluish black, thinly microtomentose, subshiny to shiny; postpronotum brown, some specimens with some yellowish coloration around margin; area from postpronotum and through notopleuron at most finely and thinly microtomentose, subshiny to shiny; pleural areas finely microtomentose, subshiny, blackish brown; anepisternum mostly bare but with 2–3 setulae along posterior margin; katepisternum generally setulose, bearing 2 setae toward dorsal margin. Wing without pattern, generally hyaline to faintly infumate. Coxae generally yellow to whitish yellow, hindcoxa yellowish brown in some specimens; trochanters yellow; basal 1/5 of femora yellow, remainder brown; tibiae mostly brown; forefemur lacking a preapical annulus; tarsi mostly yellowish basally, apical 2–3 tarsomeres becoming darker; forefemur bearing 1 seta at apical 1/3 along posteroventral surface.

**Figures 77–79.**
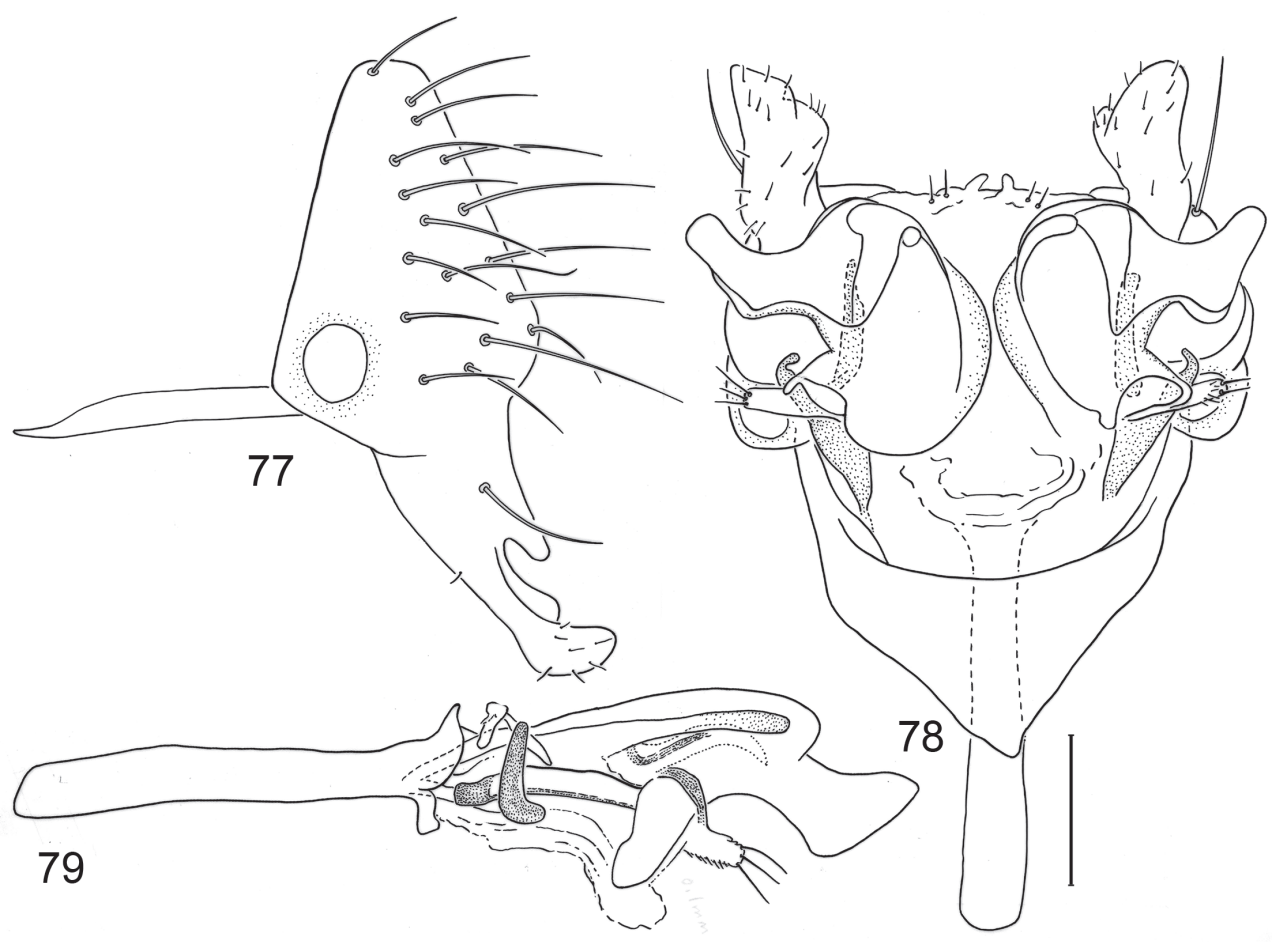
Illustrations of *Planinasus flavicoxalis* sp. n. (male). **77** epandrium, surstylus, hypandrium, lateral view **78** structures of internal male terminalia, ventral view **79** internal structures of male terminalia, lateral view. Scale bar = 0.1 mm.

*Abdomen*: Uniformly blackish brown, mostly dull to faintly subshiny, moderately invested with microtomentum. Male abdomen: Tergites 1+2–6 well developed, lengths of 3–6 subequal; tergite 7 narrow; sternites 3, 4, 5 generally as rectangular plates, slightly wider than long, lateral margins shallowly arched; sternite 5 with posterior margin of sclerotized portion shallowly emarginate posteriorly; no sternites 6, 7, neither segment forming an annulus. Male terminalia (Figs 77–79): Epandrium in lateral view (Fig. 77) narrowly trapezoidal, higher than wide, very narrowed dorsally, anterior and posterior margins nearly straight; surstylus almost as long as epandrium, extended from ventral margin of epandrium in nearly oblique alignment with it, in lateral view (Fig. 77) elongate, robustly developed, unevenly bilobed, anterior lobe much larger, slightly enlarged apically, clavate, narrowly rounded apically, short posterior lobe digitiform, narrowly rounded apically, bearing elongate setula sub-basally near middle; hypandrium in ventral view (Fig. 78) broadly and robustly U- to V-shaped, posterior margin U-shaped, anterior margin V-shaped, arms tapered, narrowed toward attachment with epandrium, anterior portion robustly developed, anterior margin conspicuously pointed; postgonite in ventral view convoluted (Fig. 78) with lateral arms spread out and longer than wide, lobe bearing a few setulae, in lateral view (Fig. 79) longer than wide, lone digitiform, bearing 3 apical, short setulae; phallus in ventral and lateral views (Figs 78, 79) mostly membranous, difficult to distinguish; phallapodeme in lateral and ventral views (Figs 78–79) elongate, parallel sided, nearly straight; ejaculatory apodeme greatly reduced.

#### Description of female.

As in male except as follows: Head generally slightly narrower, head ratio 0.70–75; frontal ratio 0.63–0.67; facial ratio 0.31–0.36.

#### Type material.

The holotypes male is labeled “DOMINICA, W.I[.] trail,1mi.n.junc. rds to Rosalie & Castle Bruce, 1300' Apr. 23, 1966[,] R.J. Gagne/Bredin-Archbold-Smithsonian Bio.Surv.Dominica/USNM ENT 00118283 [plastic bar code label]**HOLOTYPE** ♂ *Planinasus flavicoxalis* Mathis & Rung USNM [red].” The holotype is double mounted (glued to a paper triangle), is in good condition (hindlegs broken, one glued to dorsum of paper triangle), and is deposited in the USNM. Paratypes are as follows: West Indies. *DOMINICA*. Boeri Lake (15°21.1'N, 61°19.2'W), 22 Feb 1964, D. F. Bray (1♂; USNM); Fond Figues River (15°23.9'N, 61°18.2'W; 122 m), 29 Apr 1966, R. J. Gagne (1♂; USNM); Freshwater Lake (15°21.1'N, 61°19.2'W; 792 m), 21 Jan-23 Mar, 1964, 1965, T. M. Clarke, D. F. Bray (1♂, 1♀; USNM); Pont Cassé (15°22'N, 61°21'W), 23 Nov 1964, P. J. Spangler (1♀; USNM); Pont Cassé (2.75 km E; 15°21'N, 61°19'W), 10 Mar 1965, W. W. Wirth (1♂; USNM); Pont Cassé (0.8 km E; 15°21'N, 61°20'W), 11 Apr 1966, R. J. Gagné (1♀; USNM); Pont Cassé (3.2 km NW; 15°23'N, 61°22'W), 5 Jun 1965, D. R. Davis (1♂; USNM); Sylvania (15°21'N, 61°22.2'W), 10 Feb 1964, H. Robinson (1♂; USNM).

#### Type locality.

West Indies. Dominica. St. David: 1.6 km N of junction of roads to Rosalie and Castle Bruce (15°23.8'N, 61°18.6'W; 396 m).

#### Distribution

(Fig. 43). *Neotropical*: West Indies (Dominica).

#### Etymology.

The specific epithet, *flavicoxalis*, is of Latin derivation and refers to the yellowish coxae of this species.

#### Remarks.

Externally this species is very similar to *Planinasus atrifrons* sp. n and *Planinasus atriclypeus* sp. n. Besides the characters noted in the key, the shape of the surstylus, hypandrium and postgonite is sufficient to distinguish this species from these congeners.

### 16. Planinasus mcalpineorum sp. n.

urn:lsid:zoobank.org:act:2A86FEF8-971E-4E4E-A471-D223CB1AFB48

http://species-id.net/wiki/Planinasus_mcalpineorum

**Figures 80–81.**
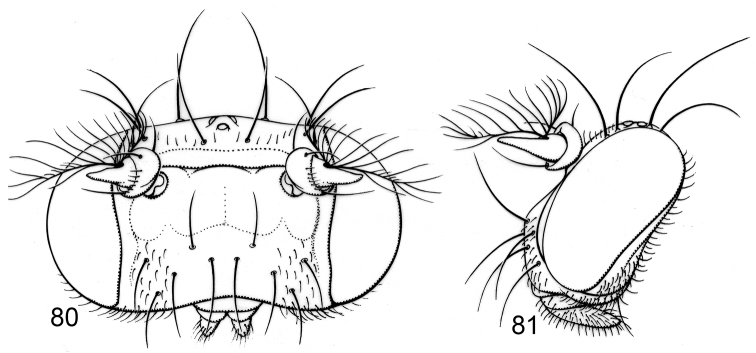
Illustrations of *Planinasus mcalpineorum* sp. n. (male). **80** head, anterior view **81** same, lateral view.

Figures 80 [Fig F33] –86

#### Description of male.

Moderately small to medium-sized flies, body length 2.45–3.15 mm.

*Head* (Figs 80–81): Head ratio 0.56–0.58; frons generally brownish black to black, mostly very finely and sparsely microtomentose, subshiny, except for densely microtomentose, velvet-like anterolateral angles; latter setulose; immediate anterior margin moderately microtomentose, dull; frons conspicuously wider than long, frontal ratio 0.35–0.36; interfrontal seta shallowly curved, elongate, length subequal to length of lateral vertical seta. Antenna brownish black to black; basal flagellomere moderately long, length conspicuously greater than width at base, tapered to moderately acute point at apex, both dorsal margin shallowly depressed; pedicel with ventral projection short, not extended anteriorly much beyond dorsal margin, bearing long, ventroapical seta (extended slightly beyond apex of basal flagellomere), 1 dorsal seta, 1 dorsomedial seta, 1 medial seta; arista bearing 13–14 dorsal rays, 3–4 ventral rays. Face comparatively wide, facial ratio 0.80–0.82; dorsad of transverse carina sparsely microtomentose, subshiny to shiny, mostly blackish brown, but dorsal margin partially yellowish; ventrad of transverse carina densely microtomentose, silvery white with some faint yellowish, sericeous; large facial setae not arranged in transverse row; medial pair of setae approximate, shallowly ventroclinate; next seta curved dorsally; next seta ventroclinate, inserted ventrad of medial pair; lateral 2 setae ventroclinate, slightly aligned vertically. Clypeus black with whitish gray microtomentum; palpus brownish black.

*Thorax*: Mesonotum generally brownish black to deep bluish black, sparsely microtomentose, subshiny to shiny; postpronotum brown with some yellowish coloration around margin; area from postpronotum and through notopleuron mostly bare, subshiny, at most finely microtomentose; pleural areas finely microtomentose, subshiny, blackish brown; anepisternum mostly bare but with 3–4 setulae along posterior margin; katepisternum generally setulose, bearing 2 setae toward dorsal margin. Wing without pattern, generally hyaline, only very slightly infumate. Coxae brown with gray to whitish gray microtomentum; trochanters yellowish brown to yellow; femora and tibiae uniformly brownish black; forefemur lacking a preapical annulus; tarsi mostly yellowish, apical 2–3 tarsomeres becoming darker; forefemur bearing 1 seta at apical 1/3 along posteroventral surface.

**Figures 82–85. F33:**
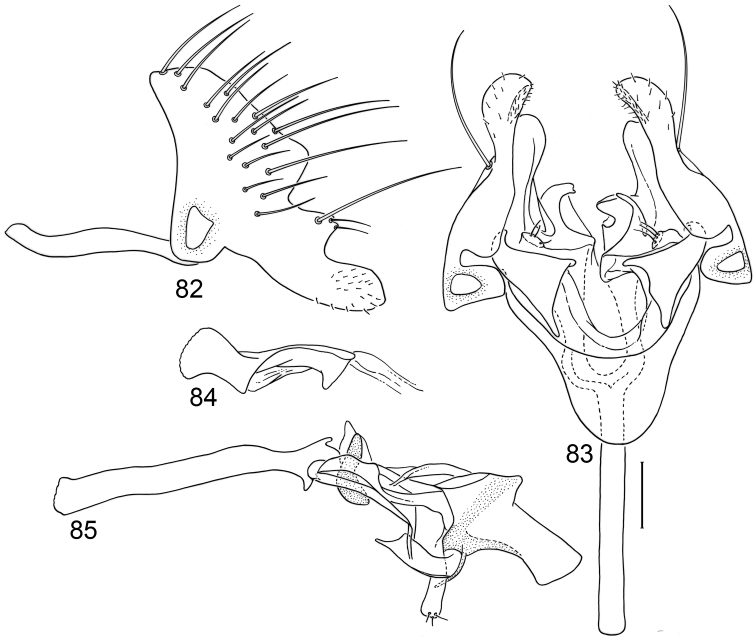
Illustrations of *Planinasus mcalpineorum* sp. n. (male). **82** epandrium, surstylus, and hypandrium, lateral view **83** structures of internal male terminalia, ventral view **84** ejaculatory apodeme, lateral view **85** internal structures of male terminalia, lateral view. Scale bar = 0.1 mm.

*Abdomen*: Uniformly blackish brown, mostly dull to faintly subshiny, modera-tely invested with microtomentum. Male abdomen: Tergites 1+2–6 well developed, lengths of 3–6 subequal; tergite 7 well developed but narrow, band-like; sternites 3–5 well developed, rectangular, wider than long, lateral margins shallowly arched; sclerotized posterior margin of sternite 5 very shallowly emarginate, very broadly V-shaped; sternite 6 apparently lacking; sternite 7 moderately well developed, wider than long, with an oblique, posterior, thumb-like, short process, sternite not fused with tergite 7 to form an annulus. Male terminalia (Figs 82–85): Epandrium in lateral view (Fig. 82) trapezoidal, higher than wide, narrowed dorsally, anterior margin shallowly emarginate, posterior margin shallowly sinuous; surstylus almost as long as epandrium, extended from ventral margin of epandrium in nearly oblique alignment with it, in lateral view (Fig. 82) wide, robustly developed basally, anterior margin shallowly arched, posterior distinctly and step-wise angulate, forming a medial angle bearing an elongate seta, apical half robustly developed, thumb-like; hypandrium in ventral view (Fig. 83) parallel sided, elongate, narrowly bar-like; postgonite in ventral view (Fig. 83) convoluted with short lobe, little extended, bearing 3 apical setulae, lobe in lateral view narrow, longer than wide; phallus in ventral and lateral views (Figs 83, 85) mostly membranous and difficult to discern; phallapodeme in lateral and ventral views (Figs 83) elongate, parallel sided, bluntly rounded apically; ejaculatory apodeme reduced, about as long as half that of phallapodeme, longer than wide, apex slightly dilated but not fan-like.

**Figure 86.**
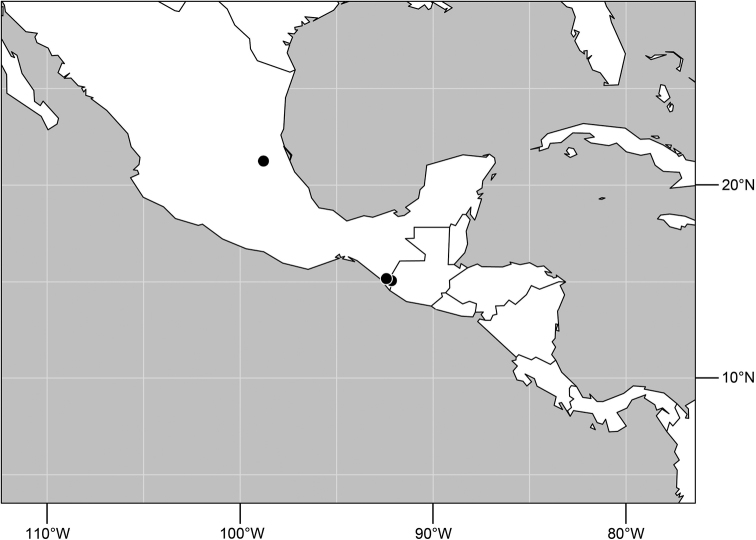
Distribution of *Planinasus mcalpineorum* sp. n.

#### Description of female.

As in male except as follows: Head generally narrower, head ratio 0.63–0.65; frontal ratio 0.43–0.45; facial ratio 0.49–0.53.

#### Type material.

The holotype male is labeled “**MEXICO:** Chiapas[:] Cacahoatan, 7kmN[,] 22 April 1983[,] Wayne N. Mathis/USNM ENT 00118282 [plastic bar code label]/**HOLOTYPE** ♂ *Planinasus mcalpineorum* Mathis & Rung USNM [red]. The holotype is double mounted (minuten pin in a plastic block), is in excellent condition, and is deposited in the USNM. Five paratypes (1♂, 4♀; USNM) bear the same label data as the holotype. Other paratypes are as follows: MEXICO. *Chiapas*: Huixtla (NE; 15°10.3'N, 92°25.4'W; 915 m), 1 Jun 1969, H. J. Teskey (2♂; CNC). *San Luis Potosí*: Tamazunchale (near; 21°15.2'N, 98°47.3'W), 5 Aug 1962 (1♂, 1♀; USNM).

#### Type locality.

Mexico. Chiapas: Cacahoatán (7 km N; 15°04.1'N, 92°07.4'W).

#### Distribution

(Fig. 86). *Neotropical*: Mexico (Chiapas, San Luis Potosí).

#### Etymology.

The species epithet, *mcalpineorum*, is a Latin genitive patronym to honor both David K. McAlpine (Australia) and J. Frank McAlpine (Canada) for their numerous and excellent contributions to our knowledge of Acalyptrate Diptera.

#### Remarks.

This species is known thus far only from southern Mexico. We suspect that it will be found to be more widespread once there is better sampling from other Central American countries. It is the only species of the *nigrifacies* group that has a conspicuously sclerotized ejaculatory apodeme.

### 17. Planinasus nigrifacies sp. n.

urn:lsid:zoobank.org:act:30CD293D-E230-4142-B007-ECF8ECE36B2D

http://species-id.net/wiki/Planinasus_nigrifacies

Figures 87 –90

#### Description of male.

Moderately small to medium-sized flies, body length 2.50–3.50 mm.

*Head*: Head ratio 0.64–0.66; frons generally brownish black to black, mostly very finely and sparsely microtomentose, subshiny to shiny, except for densely microtomentose, velvet-like anterolateral angles and anterior margin; anterolateral angles setulose; frons wider than long, frontal ratio 0.45–0.47; interfrontal seta shallowly curved, elongate, length subequal to length of lateral vertical seta. Antenna black; basal flagellomere variable but usually short, length usually not exceeding height at base, tapered to moderately acute point at apex, both dorsal and ventral margins nearly straight to very shallowly depressed dorsally and arched ventrally; pedicel with ventral projection short, length of projection conspicuously less than length of pedicel without considering projection, bearing long, ventroapical seta (extended slightly beyond apex of basal flagellomere), 1 dorsal seta, 1 dorsomedial seta, 1 medial seta; arista bearing 13–14 dorsal rays, 3–4 ventral rays. Face comparatively narrow, facial ratio 0.45–0.48; dorsad of transverse carina sparsely microtomentose, subshiny to shiny, mostly brownish black to deeply bluish black; ventrad of transverse carina densely microtomentose, silvery white, sericeous; large facial setae arranged in 2 transverse rows of 6 setae; medial pair of dorsal row widely separate, shallowly dorsoclinate; next 2 setae shallowly ventroclinate; ventral row of facial setae all ventroclinate. Clypeus black with whitish gray microtomentum; palpus brownish black.

*Thorax*: Mesonotum generally brownish black to deep bluish black, thinly microtomentose, subshiny to shiny; postpronotum brown, some specimens with some yellowish coloration around margin; area from postpronotum and through notopleuron at most finely and thinly microtomentose, subshiny to shiny; pleural areas finely microtomentose, subshiny, blackish brown; anepisternum mostly bare but with 2–3 setulae along posterior margin; katepisternum generally setulose, bearing 2 setae toward dorsal margin. Wing without pattern, generally infumate with some areas more hyaline. Coxae generally brown to brownish black with gray to whitish gray microtomentum; midcoxa with medial portion yellowish, lateral portion blackish brown; trochanters yellowish brown; femora and tibiae uniformly brownish black; forefemur lacking a preapical annulus; tarsi mostly yellowish basally, apical 2–3 tarsomeres becoming darker; forefemur bearing 1 seta at apical 1/3 along posteroventral surface.

**Figures 87–89.**
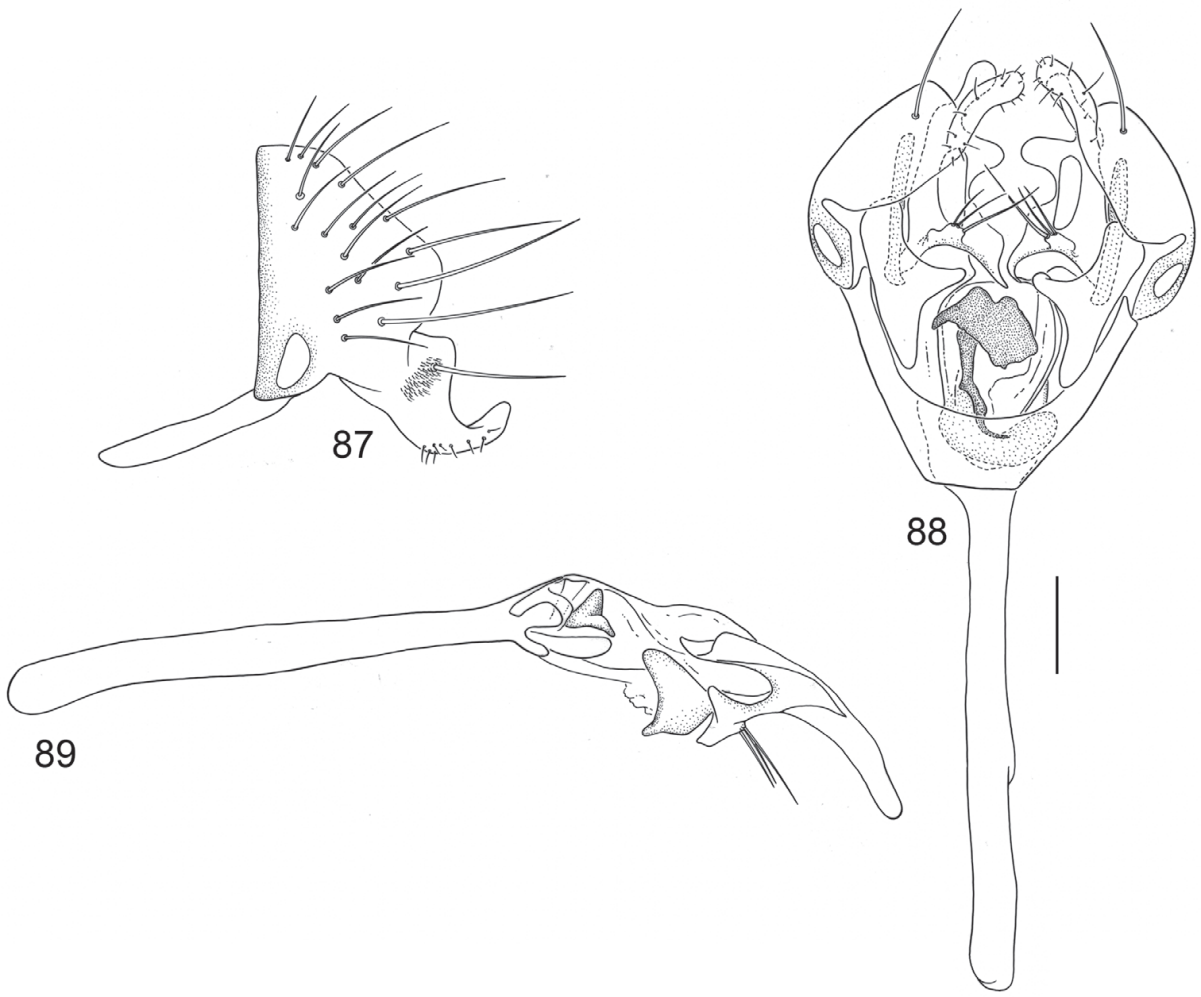
Illustrations of *Planinasus nigrifacies* sp. n. (male). **87** epandrium, surstylus, hypandrium, lateral view **88** structures of internal male terminalia, ventral view **89** internal structures of male terminalia, lateral view. Scale bar = 0.1 mm.

*Abdomen*: Uniformly blackish brown, mostly dull to faintly subshiny, moderately invested with microtomentum. Male abdomen: Tergites 1+2–6 well developed, lengths of 3–6 subequal; tergite 7 well developed but narrow, band-like; sternites 3–5 well developed, rectangular, wider than long, lateral margins shallowly arched; sclerotized posterior margin of sternite 5 shallowly incised medially; sternite 6 apparently lacking; sternite 7 moderately well developed, much wider than long, but not fused with tergite 7 to form an annulus. Male terminalia (Figs 87–89): Epandrium in lateral view (Fig. 87) trapezoidal, higher than wide, narrowed dorsally, anterior margin nearly straight, posterior margin straight on dorsal 2/3 thereafter ventrally forming a robust truncate extension; surstylus almost as long as epandrium, extended from ventral margin of epandrium in nearly oblique alignment with it, in lateral view (Fig. 87) elongate, robustly developed basally, distinctly tapered, apex recurved, anterior margin deeply arched, posterior margin swollen medially, apical 1/4 tapered to moderately acute point, partially recurved, bearing medial, elongate setula from posteromedial extension; hypandrium in ventral view (Fig. 88) moderately robustly U- to V-shaped, posterior margin U-shaped, anterior margin truncate, arms tapered toward attachment with epandrium, anterior portion robustly developed with lateral margins nearly straight; postgonite in ventral view (Fig. 88) convoluted, with relatively short lobe bearing apical setula, in lateral view (Fig. 89) with lobe very shallowly extended, bearing 3 apical, short setulae; phallus in ventral view and lateral views (Figs 88, 89) mostly membranous and difficult to discern; phallapodeme in lateral and ventral views (Figs 88–89) elongate, irregularly parallel sided, truncate apically; ejaculatory apodeme greatly reduced.

**Figure 90.**
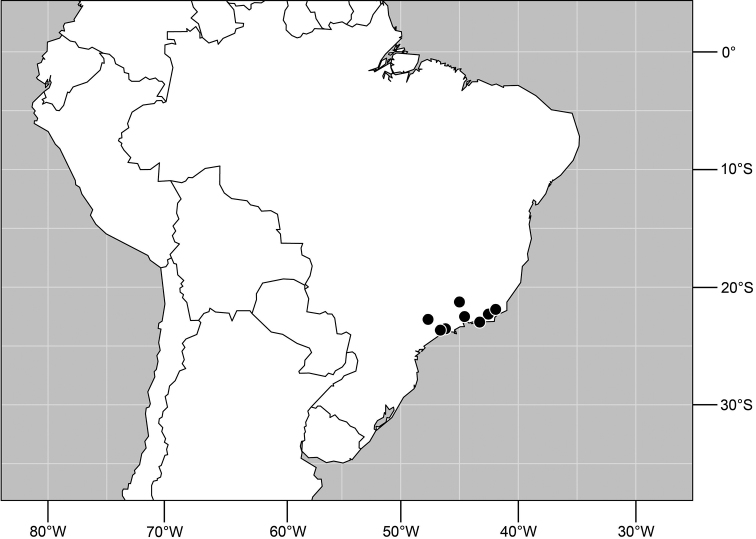
Distribution of *Planinasus nigrifacies* sp. n.

#### Description of female.

As in male except as follows: Head generally narrower, head ratio 0.86–89; frontal ratio 0.54–0.57; facial ratio 0.33–0.36.

#### Type material. 

The holotype male is labeled “**BRAZIL.** São Paulo: Mogi das Cruzes, Serra do Itapeti, 29 March 1992[,] R. & A. Baptista/USNM ENT 00118280 [plastic bar code label]/**HOLOTYPE** ♂ *Planinasus nigrifacies* Mathis & Rung MZUSP [red].” Ten paratypes (7♂, 3♀; MZUSP, USNM) bear the same label data as the holotype. The holotype is double mounted (glued to a paper point), is in very good condition (some cephalic setae misoriented), and is deposited in MZUSP.

#### Type locality.

Brazil. São Paulo: Mogi das Cruzes, Serra do Itapeti (23°31.5'S, 46°11.2'W).

#### Other specimens examined.

*BRAZIL. Minas Gerais*: Lavras (21°14.7'S, 45°W), 20 Jul 1992, A. and R. Baptista (11♂, 8♀; MZUSP, USNM). *Rio de Janeiro*: Nova Friburgo (22°17.2'S, 42°32'W), 1 May 1995, A. and R. Baptista (3♂; USNM). Rio de Janeiro, Floresta da Tijuca (22°57.6'S, 43°16.4'W), 30 Jul 1991, A. and R. Baptista (1♂; MZUSP); Parque Estadual do Desengano (near Santa Maria Madgalena; 21°53'S, 41°55'W), 19–23 Dec 1991, A. and R. Baptista (1♂; MZUSP); Parque Nacional de Itatiaia, Maromba (22°29.7'S, 44°33.7'W), 13–17 Dec 1991, A. and R. Baptista (2♀; MZUSP). *São Paulo*: São Paulo, Jaraguá (22°44.1'S, 47°40'W), 9 Dec 1990, A. and R. Baptista (1♂; MZUSP); São Paulo, Jardim Botânico (23°39'S, 46°37'W), 11 Feb 1992, A. and R. Baptista (1♂, 1♀; MZUSP).

#### Distribution

(Fig. 90). *Neotropical*: Brazil (Minas Gerais, Rio de Janeiro, São Paulo).

#### Etymology.

The species epithet, *nigrifacies*, is derived from Latin and refers to the black face, including the all black antenna.

#### Remarks.

In the key to species, this species is close to *Planinasus mcalpineorum*. Besides the distribution and characters given in the key, the unique shape of the surstylus (Fig. 87) can be used to distinguish this species readily from congeners.

##### The *obscuripennis* group

**Included species.** *Planinasus obscuripennis* sp. n.

**Diagnosis.** This species group is distinguished by the following combination of characters: *Head*: Interfrontal seta elongate, length subequal to length of lateral vertical seta. Scape and pedicel black; basal flagellomere pale, mostly yellowish on basal but black apically and faintly so dorsally, elongate, length much greater than height at base, tapered to moderately acute apex, dorsal margin shallow depressed, ventral margin shallowly arched. Large facial setae arranged in 2 transverse rows; dorsal transverse row consisting of 1 pair of separate, well-developed, dorso- to inclinate setae; ventral row arched, comprising 6 ventroclinate setae about equidistant from peristomal margin; face of males and females similar in shape and color. *Thorax*: Anepisternum mostly bare but with 2–3 setulae along posterior margin. Wing generally conspicuously and darkly infumate with base more hyaline. Forefemur with a preapical annulus, bearing 1 seta at apical 1/3 along posteroventral surface. *Abdomen*: Surstylus in nearly oblique alignment with epandrium, not a posterior process, or lobe; postgonite with a digitiform lobe bearing a few setulae apically; phallus mostly sclerotized; aejaculatory apodeme greatly reduced.

**Discussion.** This species group currently comprises a single species, *Planinasus obscuripennis*. It shares, with species of the *atriclypeus* and *shannoni* groups, the reduced number of setae on the lobe of the postgonite (2–6), and the ejaculatory apodeme is generally reduced or inconspicuous. In all other species of *Planinasus*, the lobe of the postgonite, which is generally better developed, bears more than 20 setulae, and the ejaculatory apodeme is large, with a fan-like apical expansion. We recognize this group by the characters noted above but also by the unique structures associated with the male pre- and postabdomen. Among these characters are the apparent fusion of sternites 4+5 and a well-developed sternite 6, which is broadly produced medioposteriorly to form a shallowly bifurcate projection. In addition, the ejaculatory apodeme is short and its apex is not expanded.

### 18. Planinasus obscuripennis sp. n.

urn:lsid:zoobank.org:act:31369FB9-F66F-4615-B07C-002DBE75331F

http://species-id.net/wiki/Planinasus_obscuripennis

Figures 52 , 91–93

#### Description of male.

Medium-sized flies, body length 3.00–3.15 mm.

*Head*: Head ratio 0.71; frons generally brownish black to black, mostly very finely and sparsely microtomentose, subshiny, except for densely microtomentose, anterolateral angles and anterior margin; anterolateral angles setulose; frons moderately wider than long, frontal ratio 0.49; interfrontal seta shallowly curved, elongate, length subequal to length of lateral vertical seta. Scape and pedicel black; basal flagellomere pale, mostly yellowish on basal but black apically and faintly so dorsally, elongate, length much greater than height at base, tapered to moderately acute apex, dorsal margin shallow depressed, ventral margin shallowly arched; pedicel with ventral projection short, length of projection conspicuously less than length of pedicel without considering projection, bearing long, ventroapical seta (extended slightly beyond apex of basal flagellomere), 1 dorsal seta, 1 dorsomedial seta; arista bearing 13–14 dorsal rays, 3–4 ventral rays. Face comparatively narrow, facial ratio 0.35; dorsad of transverse, arched carina sparsely microtomentose, subshiny, sericeous, broad medial portion brown to brownish yellow anteriorly; antennal grooves nearly flat, brownish black; ventrad of transverse carina more densely microtomentose, yellow medially, lateral areas blackish brown; large facial setae arranged in 2 transverse rows; dorsal transverse row consisting of 1 pair of separate, well-developed, dorso- to inclinate setae; ventral row arched, comprising 6 ventroclinate setae about equidistant from peristomal margin. Clypeus black with sparse whitish gray microtomentum; palpus brownish black.

*Thorax*: Mesonotum generally brownish black to deep bluish black, thinly microtomentose, subshiny to shiny; postpronotum brown, some specimens with some yellowish coloration around margin; area from postpronotum and through notopleuron at most finely and thinly microtomentose, subshiny to shiny; pleural areas finely microtomentose, subshiny, blackish brown; anepisternum mostly bare but with 2–3 setulae along posterior margin; katepisternum generally setulose, bearing 2 setae toward dorsal margin. Wing without pattern, generally conspicuously and darkly infumate with base more hyaline. Coxae generally brown to brownish black with gray to whitish gray microtomentum; midcoxa with medial portion yellowish, lateral portion blackish brown; trochanters yellow to yellowish brown; femora and tibiae uniformly brownish black to black; forefemur with a preapical annulus; tarsi mostly yellowish basally, apical 2–3 tarsomeres becoming darker; forefemur bearing 1 seta at apical 1/3 along posteroventral surface.

**Figures 91–93.**
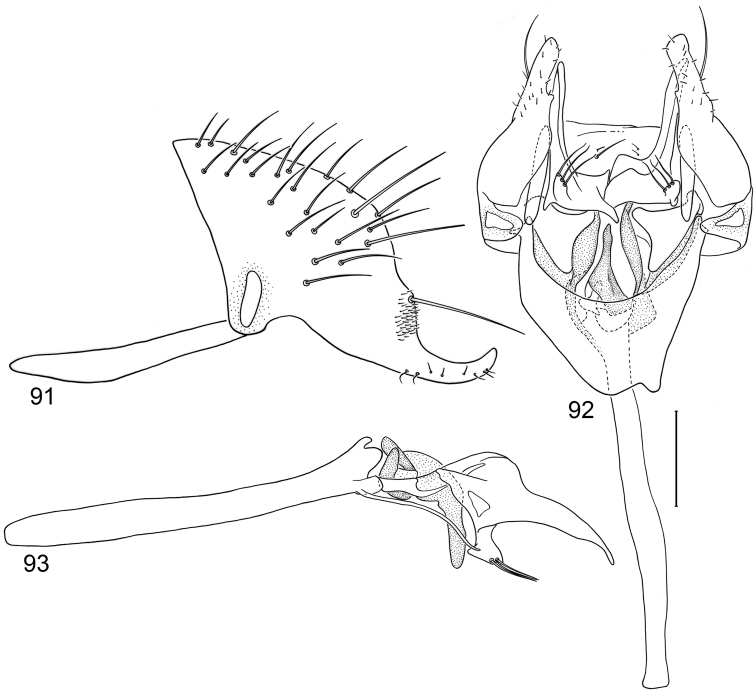
Illustrations of *Planinasus obscuripennis* sp. n. (male). **91** epandrium, surstylus, hypandrium, lateral view **92** structures of internal male terminalia, ventral view **93** internal structures of male terminalia, lateral view. Scale bar = 0.1 mm.

*Abdomen*: Male abdomen: Tergites 1+2–6 well developed, lengths of 3–6 subequal; sternite 3 slightly longer than wide, anterior margin shallowly arched; sternites 4+5 apparently fused, slightly longer than wide; sternite 6 well developed, longer than wide, lateral margins bearing longer setulae, posterior margin produced medially to form shallowly bifurcate, broad projection, each apex of shallow bifurcation bearing longer setae; tergite 7 very narrow, almost forming a complete annulus, narrowly separated from subquadrate sternite ventrally. Male terminalia (Figs 91–93): Epandrium in lateral view (Fig. 91) triangular, length of ventral portion subequal to height, anterior margin nearly straight to shallowly emarginate, posterior margin shallowly arched; surstylus as long as epandrium, extended from posteroventral margin of epandrium in nearly oblique alignment with it, in lateral view (Fig. 91) elongate, robust basally, thereafter apically tapered, apex more thinly developed, shallowly curved, bearing 1 large, elongate, posteromedial setula; hypandrium in lateral view (Fig. 91) elongate, narrow, nearly straight and parallel sided, in ventral view (Fig. 92) broadly U-shaped, robustly developed anteriorly, arms tapered, more slender than wide base, anterior margin irregularly rounded; pregonite in ventral view (Fig. 92) approximately triangular with anterior margin receded and slightly round corners; postgonite in ventral view convoluted (Fig. 92), bearing short lobe with apical setulae, in lateral view (Fig. 93) lobe only slightly longer than wide, bearing three apical setulae; phallus in ventral view (Fig. 92) complex, mostly sclerotized, tubular; phallapodeme elongate, slender, in lateral view (Fig. 93) parallel sided, tubular, nearly straight, rounded apically, in ventral view (Fig. 92) tapered, margins shallowly undulous, apex narrowly developed; ejaculatory apodeme much reduced.

#### Description of female.

As in male except as follows: Head generally narrower, head ratio 0.70; frontal ratio 0.56; facial ratio 0.35.

#### Type material.

The holotype male is labeled “PERU.Madre de Dios: Manu, Erika (near Salvacion),550m,5–6 Sept1988,AFreidberg/**HOLOTYPE** ♂ *Planinasus obscuripennis* Mathis & Rung USNM [red]/USNM ENT 00118132 [plastic bar code label]/Illustrated ♂ [handwritten].” The holotype is double mounted (minuten in a block of plastic elastomer], is in good condition (some encrustation on head, especially eyes), and is deposited in the USNM. A female paratype (USNM) bears the same label data as the holotype.

#### Type locality.

Peru. Madre de Dios: Río Manu, Erika (near Salvación; 12°50.7'S, 71°23.3'W; 550 m).

#### Distribution

(Fig. 52). *Neotropical*: Peru (Madre de Dios).

#### Etymology.

The specific epithet, *obscuripennis*, is of Latin derivation and refers to the infumate wing of this species.

#### Remarks.

The male abdomen of this species is atypical of other congeners in having apparent fusion of some sternites (sternites 4+5) and a well-developed sternite 6 that is broadly produced medioposteriorly to form a shallowly bifurcate projection. We have observed this condition only in this species, and our observation is limited to the single holotype male.

##### Fossil species

### 19. Planinasus electrus

Grimaldi & Mathis

http://species-id.net/wiki/Planinasus_electrus

Planinasus electrus Grimaldi and Mathis 1993: 396. Fossil; Dominican Republic. HT ♀ AMNH [AMNH type number DR-8–208; specific provenance unknown; size of amber 1.1 X 1.3 cm, nearly flat]. Evenhuis 1994: 424 [review]. Mathis and Rung 2011: 363 [world catalog].

#### Description of female.

Moderately small flies, body length 2.20 mm; wing length 1.71 mm.

*Head*: Width 0.78 mm; facial width at narrowest point 0.25 mm; frons dark brown, glabrous; interfrontal seta short, about half length of lateral vertical seta, reclinate, separated by slightly more than distance between outside margins of posterior ocelli; postocellar seta lacking; proclintate and reclinate fronto-orbital setae approximately equal in length, reclinate seta inserted slightly medial to proclinate seta, separated from latter by about 3 times its diameter at base; medial and lateral vertical setae about equal in length. Antenna bicolored, scape and pedicel brown, basal flagellomere pale, mostly whitish yellow with pale brown area immediately around base of arista; scape very thin; antennal bases separated by about diameter of pedicel; pedicel with deep anterior cleft; ventral projection of pedicel short, not extended anteriorly much beyond dorsal margin; medial half of pedicel with 2 black, stout setae of equal length, both oriented anteriad, parallel; lateral half of pedicel with thin seta dorsally, near cleft, and 4–5 fine, smaller setulae in row on anterolateral margin; flagellomere short, width about 2/3 length; arista with lateral trunk bearing 4 branches all on one side; apical branch pair smallest; main ramus of arista with 4 large dorsal branches, plus terminal fork; 4 smaller medial branches; 2 ventral branches; both rami of arista with a common base, but no common ramus. Face bicolored, mostly pale, whitish yellow, but with oral margin and narrow medial extension brown; facial setae in 2 irregular rows; setae along ventral row of larger medial setae in row along sloping line at transition between pale and brown color. 2 other facial setae inserted dorsad of longer row, between large dorsoclinate setae and anteroventral margin of eye (seta inserted closer to eye almost twice length of seta inserted between it and dorsoclinate setae). Proboscis pale; palpus thin, pale.

*Thorax*: Length 0.74 mm; mesonotum and pleuron dark brown; anterior dorsocentral seta about midway between anterior margin of notum and noto-scutellar suture; posterior dorsocentral seta inserted closer to scutellum than to anterior dorsocentral seta; acrostichal setulae minute, barely discernible; katepisternum with 1 larger seta, 3–4 much small setae; anepisternum with row of about 4 fine setulae along posterior margin. Legs: coxae and femora brown; tibiae mostly brown, apical 1/4-1/5 pale; length of basitarsomeres about equal to length of all 4 distal tarsomeres; tarsomeres 1–4 pale; apical tarsomeres brown; midtibia bearing several small setae (length about ½ that of tibia width); 1 ventroapical, 1 preapical lateral, and pair of dorsoapical. Halter pale. Wing: Length 1.71 mm; hyaline; vein R_1_ thickened at merger with C; Sc very short, incomplete; subcostal break present, but not well defined; vein R_2+3_ extended to about apical 1/3 of R_4+5_; veins R_4+5_ and M parallel; apices of veins CuA_1_ and A_1_ tapered, not extended to wing margin cells cup, bm, and dm complete.

*Abdomen*: Relatively flat dorsoventrally; tapered apically. Tergites, epiproct, and hypoproct uniformly brown.

#### Type material.

The holotype female is preserved in Dominican amber (Dominican Republic. specific provenance unknown) and is deposited in the AMNH (type number DR-8-208).

## Supplementary Material

XML Treatment for Periscelididae

XML Treatment for Stenomicrinae

XML Treatment for Planinasus

XML Treatment for Planinasus aenigmaticus

XML Treatment for Planinasus ambiguus

XML Treatment for Planinasus neotropicus

XML Treatment for Planinasus kotrbae

XML Treatment for Planinasus miradorus

XML Treatment for Planinasus shannoni

XML Treatment for Planinasus tobagoensis

XML Treatment for Planinasus venezuelensis

XML Treatment for Planinasus xanthops

XML Treatment for Planinasus argentifacies

XML Treatment for Planinasus insulanus

XML Treatment for Planinasus nigritarsus

XML Treatment for Planinasus atriclypeus

XML Treatment for Planinasus atrifrons

XML Treatment for Planinasus flavicoxalis

XML Treatment for Planinasus mcalpineorum

XML Treatment for Planinasus nigrifacies

XML Treatment for Planinasus obscuripennis

XML Treatment for Planinasus electrus

## References

[B1] Baptista AR Mathis WN (1994) A revision of the New World species of the genus *Cyamops* Melander (Diptera: Periscelididae). Smithsonian Contribution to Zoology 563: iv+1–28.

[B2] Clausen PJ Cook EF (1971) A revision of the Nearctic species of the tribe Parydrini (Diptera: Ephydridae). Memoirs of the American Entomological Society 27: 1-150.

[B3] Cresson ET Jr (1914) Descriptions of new genera and species of the dipterous family Ephydridae. I. Entomological News 25: 241-250.

[B4] Cresson ET Jr (1918) Costa Rican Diptera collected by Philip P. Calvert, Ph.D., 1909–1910. Paper 3. A report on the Ephydridae. Transactions of the American Entomological Society 44: 39-68.

[B5] Cumming JM Sinclair BJ Wood DM (1995) Homology and phylogenetic implications of male genitalia in Diptera- Eremoneura. Entomologica Scandinavica 26 (2): 121-149. doi: 10.1163/187631295X00143

[B6] Curran CH (1934) The Families and Genera of North American Diptera. 512 pp. New York. doi: 10.5962/bhl.title.6825

[B7] Evenhuis NL (1994) Catalogue of fossil flies of the world (Insecta: Diptera). Backhuys Publishers, Leiden, 600 pp.

[B8] Griffiths GCD (1972) The phylogenetic classification of Diptera Cyclorrhapha with special reference to the structure of the male postabdomen. *In* Schimitschek E (Ed) Series Entomologica 8: 340 pp. The Hague: Dr. W. Junk N.V.

[B9] Grimaldi DA (1987) Phylogenetics and taxonomy of *Zygothrica*. Bulletin of the American Museum of Natural History 186: 103-268.

[B10] Grimaldi DA Fenster G (1989) Evolution of extreme sexual dimorphisms: structural and behavioral convergence among broad-headed male Drosophilidae (Diptera). American Museum Novitates 2939: 1-25.

[B11] Grimaldi DA Mathis WN (1993) Fossil Periscelididae (Diptera). Proceedings of the Entomological Society of Washington 95: 383-403.

[B12] Hendel F (1916) Beiträge zur Systematik der Acalyptraten Musciden (Dipt.). Entomologische Mitteilungen 5(9/12): 294–299.

[B13] Hennig W (1969) Neue Gattungen und Arten der Acalyptratae. The Canadian Entomologist 101 (6): 589-633. doi: 10.4039/Ent101589-6

[B14] Hennig W (1971) Neue Untersuchungen über die Familien der Diptera Schizophora. *Stuttgarter beiträge zur Naturkunde* 226: 1-76.

[B15] Malloch JR (1934) A remarkable new genus of the family Periscelidae. *Stylops*, 3: 52-53.

[B16] Mathis WN (1986) Studies of Psilopinae (Diptera: Ephydridae), I: A revision of the shore fly genus *Placopsidella* Kertész. Smithsonian Contributions to Zoology 430: iv+30 pp.

[B17] Mathis WN (1993) A new species and subgenus of *Periscelis* Loew from Australia (Diptera: Periscelididae). Journal of the Australian Entomological Society 32: 13-19. doi: 10.1111/j.1440-6055.1993.tb00535.x

[B18] Mathis WN Papp L (1992) A new genus of Periscelididae (Diptera) from the neotropics. Proceedings of the Biological Society of Washington 105: 366-372.

[B19] Mathis WN Papp L (1998) 3.24. Family Periscelididae. Manual of Palaearctic Diptera 3: 285-294.

[B20] Mathis WN Rung A (2004) Redescription of *Diopsosoma primum* Malloch (Diptera: Periscelididae). Revista Brasileira de Entomologia 48: 303-309. doi: 10.1590/S0085-56262004000300002

[B21] Mathis WN Rung A (2011) A world catalog and conspectus on the family Periscelididae (Diptera: Schizophora). Pp. 341–377. *In* Brake I, Thompson, FC (Eds) Contributions to Systema Dipterorum (Insecta: Diptera). Myia 12, viii + 564 pp.

[B22] McAlpine DK (1978) Description and biology of a new genus of flies related to *Anthoclusia* and representing a new family (Diptera, Schizophora, Neurochaetidae). Annals of the Natal Museum 23 (2): 273-295.

[B23] McAlpine DK (1983) A new subfamily of Aulacigastridae (Diptera: Schizophora), with a discussion of Aulacigastrid classification. Australian Journal of Zoology 31: 55-78. doi: 10.1071/ZO9820055

[B24] McAlpine DK (1997) Gobryidae, a new family of acalyptrate flies (Diptera: Diospsoidea), and a discussion of relationships of the diopsoid families. Records of the Australian Museum 49: 167-194. doi: 10.3853/j.0067-1975.49.1997.1264

[B25] McAlpine JF (1981) Morphology and Termimology--Adults [chapter 2]. *In* McAlpine JF, et al. (Eds) *Manual of Nearctic Diptera*: 1: 9–63. Ottawa: Research Branch Agriculture Canada, Monograph 27, 674 pp.

[B26] Oldenberg L (1914) Beitrag zur Kenntnis der europäischen Drosophiliden (Dipt.). Archiv für Naturgeschichte 80A(2): 1–42.

[B27] Papp L (1984) Family Stenomicridae. *In* Soós Á, Papp L (Eds) Catalogue of Palaearctic Diptera, 10: 61–62. Budapest: Elsevier Science Publishers and Akademiai Kiado.

[B28] Prado AP do (1975) 67. Family Periscelididae. *In* Papavero N (Ed) A Catalogue of the Diptera of the Americas South of the United States 67: 1–3. Sao Paulo: Departamento de Zoologia, Secretaria da Agricultura.

[B29] Rung A Mathis WN (2011) A revision of the genus *Aulacigaster* Macquart (Diptera: Aulacigastridae). Smithsonian Contributions to Zoology 633: 1-132. doi: 10.5479/si.00810282.633

[B30] Sabrosky CW (1983) A synopsis of the world species of *Desmometopa* Loew (Diptera, Milichiidae). Contributions of the American Entomological Institute 19 (8): 1-69.

[B31] Stackelberg AA (1933) Les mouches de la partie europeénne de 1’URSS. Tableaux Analytiques de la Faune de 1’URSS. Leningrad, Akademiia Nauk, 742 pp.

[B32] Stackelberg AA (1970) 70. Cem. Periscelididae [Fam. Periscelididae]. P. 216. *In* Stackelberg AA, Nartshuk EP (Eds) Keys to the insects of the European USSR, 5 (Flies, Fleas). Part 2, 943 pp. Akademiia Nauk SSSR (“Nauka”), Leningrad.

[B33] Stuckenberg BR (1999) Antennal evolution in the Brachycera (Diptera), with a reassessment of terminology relating to the flagellum. Studia Dipterologica 6: 33-48.

[B34] Sturtevant AH (1923) New species and notes on Synonymy and distribution of Muscidae calypteratae (Diptera). Novitates of the American Museum of Natural History 76: 1-12.

[B35] Sturtevant AH (1954) Nearctic flies of the family Periscelidae (Diptera) and certain Anthomyzidae referred to the family. Proceedings of the United States National Museum 103 (3332): 551-561. doi: 10.5479/si.00963801.3332.551

[B36] Winkler IS Rung A Scheffer SJ (2010) Hennig’s orphans revisited: Testing morphological hypotheses in the “Opomyzoidea. ” Molecular Phylogenetics and Evolution 54 (3): 746-762. doi: 10.1016/j.ympev.2009.12.016 20040375

[B37] Woodley NE (1982) Two new species of *Neurochaeta* McAlpine (Diptera: Neurochaetidae), with notes on cladistic relationships within the genus. Memoirs of the Entomological Society of Washington 10: 211-218.

